# A Noncanonical Tryptophan Analogue Reveals an Active Site Hydrogen Bond Controlling Ferryl Reactivity in a Heme Peroxidase

**DOI:** 10.1021/jacsau.1c00145

**Published:** 2021-05-14

**Authors:** Mary Ortmayer, Florence J. Hardy, Matthew G. Quesne, Karl Fisher, Colin Levy, Derren J. Heyes, C. Richard A. Catlow, Sam P. de Visser, Stephen E. J. Rigby, Sam Hay, Anthony P. Green

**Affiliations:** † Department of Chemistry and Manchester Institute of Biotechnology, The University of Manchester, 131 Princess Street, Manchester, M1 7DN, United Kingdom; ‡ Research Complex at Harwell, Rutherford Appleton Laboratory, Harwell Oxford, Didcot, Oxon OX11 0FA, United Kingdom; § Cardiff University, School of Chemistry, Main Building, Park Place, Cardiff CF10 3AT, United Kingdom; ∥ Kathleen Lonsdale Materials Chemistry, Department of Chemistry, University College London, 20 Gordon Street, London, Western Central 1H 0AJ, United Kingdom; ⊥ Department of Chemical Engineering and Analytical Science & Manchester Institute of Biotechnology, The University of Manchester, 131 Princess Street, Manchester, M1 7DN, United Kingdom

**Keywords:** heme enzyme, metal-oxo reactivity, hydrogen bonding, proton-coupled electron transfer, genetic code expansion, tryptophan analogue, cytochrome *c* peroxidase

## Abstract

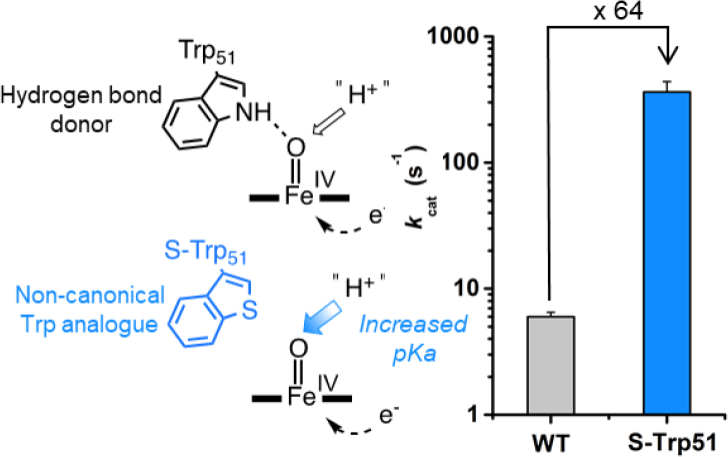

Nature employs high-energy metal-oxo intermediates embedded within enzyme active sites to perform challenging oxidative transformations with remarkable selectivity. Understanding how different local metal-oxo coordination environments control intermediate reactivity and catalytic function is a long-standing objective. However, conducting structure–activity relationships directly in active sites has proven challenging due to the limited range of amino acid substitutions achievable within the constraints of the genetic code. Here, we use an expanded genetic code to examine the impact of hydrogen bonding interactions on ferryl heme structure and reactivity, by replacing the N–H group of the active site Trp51 of cytochrome *c* peroxidase by an S atom. Removal of a single hydrogen bond stabilizes the porphyrin π-cation radical state of C*c*P W191F compound I. In contrast, this modification leads to more basic and reactive neutral ferryl heme states, as found in C*c*P W191F compound II and the wild-type ferryl heme-Trp191 radical pair of compound I. This increased reactivity manifests in a >60-fold activity increase toward phenolic substrates but remarkably has negligible effects on oxidation of the biological redox partner cyt*c*. Our data highlight how Trp51 tunes the lifetimes of key ferryl intermediates and works in synergy with the redox active Trp191 and a well-defined substrate binding site to regulate catalytic function. More broadly, this work shows how noncanonical substitutions can advance our understanding of active site features governing metal-oxo structure and reactivity.

Enzymes are the most proficient catalysts known, and consequently there is great interest in deciphering their sophisticated catalytic mechanisms. Site directed mutagenesis has been a staple technique in biochemistry for several decades as a means of elucidating the role(s) of key residues and molecular interactions.^1^ However, only a limited number of amino acid substitutions are possible as defined by nature’s genetic code. Under these constraints, substitutions designed to probe the importance of specific interactions (e.g., hydrogen bonds, π–π interactions) often lead to significant structural perturbations, making it difficult to parse out specific contributions to catalytic activity and complicating the interpretation of enzyme structure–activity relationships. This challenge is particularly acute when probing the role of the largest canonical amino acid, tryptophan, which has no close structural analogue within the genetic code. The availability of an expanded alphabet of amino acids provides a more surgical means of probing biological mechanisms by allowing substitutions of individual atoms or functional groups within proteins of interest.^2−6^ The power of this approach is exemplified through recent studies, whereby noncanonical cysteine and histidine analogues have been used to examine the role of axial heme ligands in controlling the reactivities of iconic ferryl intermediates compound I and compound II.^7−13^ These high-energy intermediates are the defining feature that drive catalysis across the entire family of heme enzymes, including P450s, peroxidases, nitric oxide synthases, and terminal oxidases.^14,15^ Consequently, there is great interest in understanding how different metal-oxo coordination environments within enzyme active sites control intermediate reactivity and overall catalytic function. Here, we use a noncanonical Trp analogue to examine directly the impact of hydrogen bonding interactions to the ferryl oxygen of compound I and compound II of cytochrome *c* peroxidase. Our data reveal how hydrogen bonding interactions are employed to control the reactivity of high-energy ferryl intermediates in enzyme active sites and thus advance our understanding of metal-oxo reactivity across a wide range of heme and nonheme iron enzymes.

C*c*P employs a heme cofactor to reduce hydrogen peroxide in mitochondria using electrons from its biological redox partner ferrous cytochrome *c* (cyt*c*).^14^ The reaction mechanism is comprised of three steps (Figure 1): (1) reaction of the resting ferric enzyme with hydrogen peroxide to generate compound I (CpdI), containing an oxidized ferryl (Fe(IV)=O) heme coupled to a neighboring Trp191 radical cation;^16^ (2) single electron reduction of CpdI by ferrous cyt*c* to generate compound II (CpdII); and (3) single electron reduction of CpdII by a second equivalent of ferrous cyt*c*. CpdII reduction is coupled with proton transfer to the ferryl oxygen, with the distal pocket His52 as the likely proton donor.^17,18^ In addition to redox active Trp191, C*c*P contains a second active site Trp51 whose N–H group forms a hydrogen bond to the ferryl oxygen of CpdI and CpdII (Figure S1).^19^ This interaction is also present in ascorbate peroxidase (APX), but is absent in many heme peroxidases, including the prototypical peroxidase from horseradish, which contains a Phe residue in place of the Trp51 of C*c*P (Figure S1).^20,21^ Interestingly, Trp51Phe and Trp51Ala substitutions in C*c*P have been shown to substantially increase the rate of nonbiological oxidations of small molecule phenolics and anilines, along with more modest increases in cyt*c* oxidation activity.^22−24^ The analogous Trp41Phe substitution in APX has also been shown to increase activity with non-native phenolic substrates.^25^ However, the molecular origins of this increased reactivity of Trp51/41 variants of C*c*P and APX are not well understood. Some have argued that steric effects dominate, and that the introduction of smaller residues provides more space and flexibility in the distal heme pocket, which could give rise to the observed activity changes.^26^ Elsewhere, the increased reactivity of Trp51 variants of C*c*P has been ascribed to an increase in activation entropy, plausibly due to a more facile release of water from the heme iron.^22^ Others have suggested that hydrogen bonding between Trp51 and the ferryl oxygen has a stabilizing effect on key intermediates.^23^

**Figure 1:**
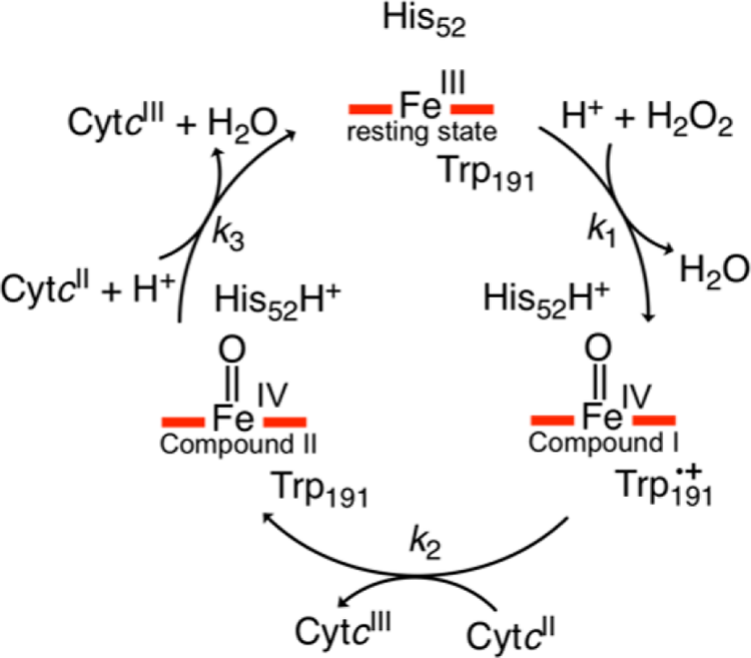
Catalytic mechanism of cytochrome *c* peroxidase.

To resolve these uncertainties, we replaced Trp51 of *Saccharomyces cerevisiae* C*c*P with 3-benzothienyl-l-alanine (S-Trp), a close structural analogue of tryptophan that cannot serve as a hydrogen bond donor, using an engineered pyrrolysyl-tRNA synthetase/pyrrolysyl-tRNA pair (PylRS_S-Trp/tRNA^Pyl^), which selectively incorporates S-Trp in response to the amber UAG stop codon.^27^ Stoichiometric replacement of the distal Trp51 residue with S-Trp was confirmed by MS analysis of the intact protein (Table S3). The X-ray crystal structure of C*c*P S-Trp (1.5 Å resolution, Table S2, Figure S2a) superimposes well with a previously reported C*c*P structure (Figure 2, PDB code: 2CYP, RMS deviation of 0.27 Å).^28^ Difference density (additional electron density) associated with the S atom of S-Trp51 was clearly visible. The geometry and environment of the heme cofactor and key active site residues are well preserved in the modified enzyme, with only minor conformational adjustments to the distal pocket His52, thus confirming Trp51S-Trp to be a highly conservative substitution. As anticipated, the X-ray crystal structure of the C*c*P S-Trp W191F double mutant is highly similar to that of C*c*P S-Trp (1.7 Å resolution, Table S2, Figure S2b), with a secondary structure superposed RMS deviation of 0.17 Å (Figure S2c).

**Figure 2:**
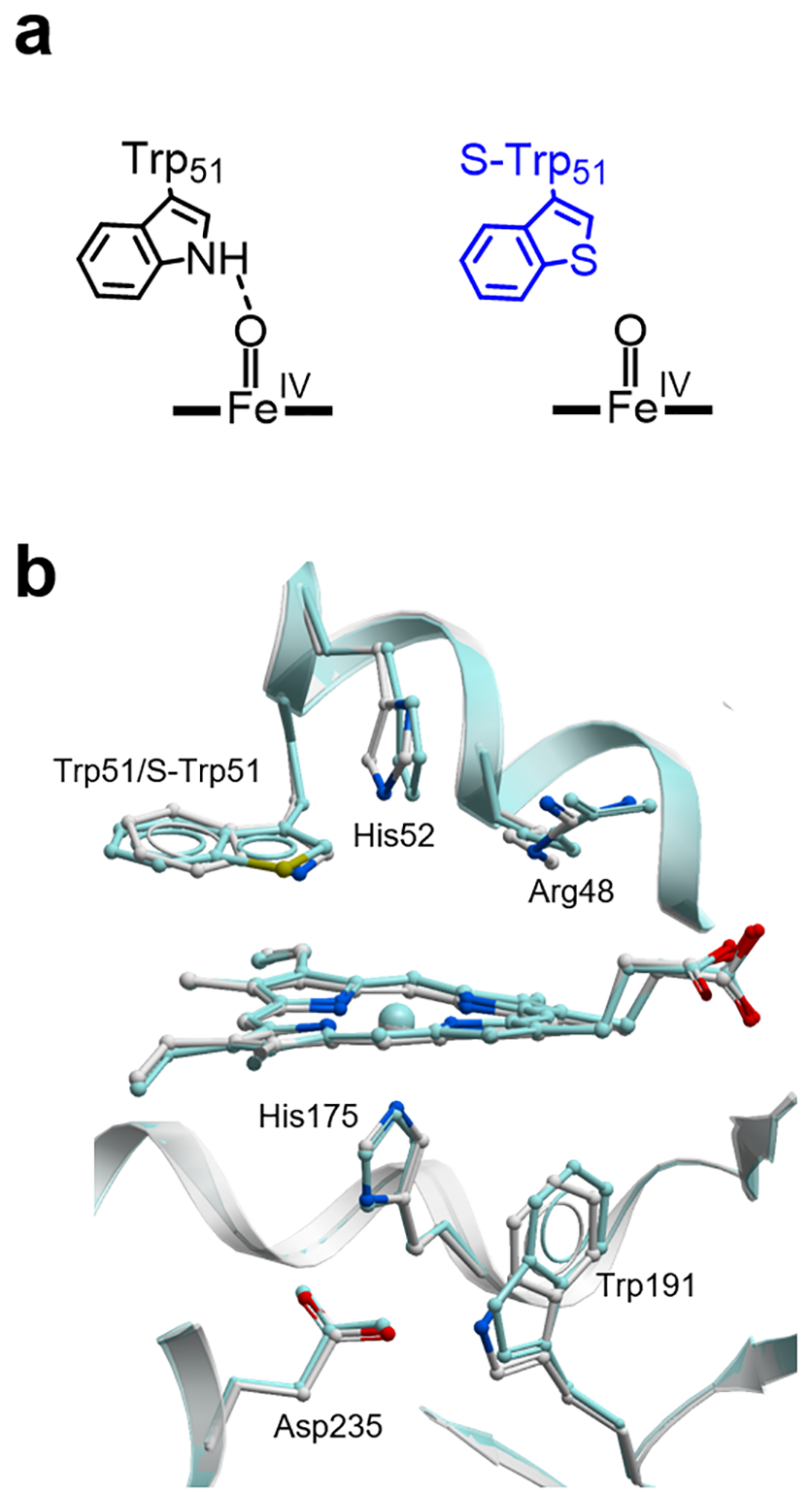
Structural characterization of C*c*P S-Trp. (a) Replacement of Trp51 with S-Trp disrupts a hydrogen bond to the ferryl oxygen of CpdI and CpdII. (b) Overlay of C*c*P (PDB code: 2CYP, gray)^28^ and C*c*P S-Trp (PDB code: 6Y1T, cyan) active sites. The heme cofactor and key residues are shown as atom-colored ball-and-sticks.

Rapid mixing stopped-flow measurements were used to examine the nature of ferryl intermediates generated upon oxidation of C*c*P and C*c*P S-Trp with hydrogen peroxide. Consistent with previous studies, mixing C*c*P with 1.5 equiv of H_2_O_2_ leads to spectral changes consistent with the formation of a neutral ferryl heme (Soret maxima at 420 nm and associated Q bands at 530 and 560 nm, Figure 3a).^29^ Similar spectral changes are observed upon oxidation of C*c*P S-Trp (Figure 3b), albeit with a slight decrease in the extinction coefficient of the Soret band (maxima at 420 nm) and Q-band features that are less well resolved than in the wild-type, suggesting that the CpdI state of C*c*P S-Trp is also comprised of a neutral ferryl heme. Time-dependent spectral changes were fitted to a sequential a → b model to derive rates for CpdI formation and are similar for both C*c*P and C*c*P S-Trp (*k* = 96 ± 3 and 100 ± 2 s^–1^, respectively). To determine the identity of the protein radical in C*c*P S-Trp, CpdI was characterized by electron paramagnetic resonance (EPR). The C*c*P S-Trp CpdI EPR line shape is very similar to that of C*c*P CpdI (Figure 3c), confirming the formation of a coupled ferryl heme-Trp191 radical pair. The small change in the downfield “shoulder” *g* value from C*c*P to C*c*P S-Trp (2.041 to 2.033) likely arises due to the well-documented sensitivity of the magnetic coupling between the Trp191 cation radical and the ferryl heme to small local structural perturbations.^30^ To further characterize the ferryl intermediate upon disruption of the hydrogen bond to Trp51, resonance Raman spectra of C*c*P and C*c*P S-Trp were recorded in both the ferric and ferryl states (Figure 3d). Hydrogen bonding interactions to ferryl intermediates are thought to give rise to an increased Fe–O bond length and an associated reduction in Fe–O stretching frequencies.^31^ The intensity of the Raman feature associated with ferric C*c*P at 757 cm^–1^ diminishes upon oxidation with H_2_O_2_, giving rise to a broad ferryl peak at 753 cm^–1^, in accordance with the literature.^32^ Oxidation of C*c*P S-Trp also diminishes the 757 cm^–1^ feature of the ferric state but instead leads to a new feature at 791 cm^–1^ (Figure 3d), which we assign as the Fe–O stretch. The feature at 791 cm^–1^ is also observed in the C*c*P S-Trp W191F double mutant (Figure S3). Density Functional Theory (DFT) models (*vide infra)* of the C*c*P ferryl state predict that the Trp51S-Trp substitution leads to a ∼0.02 Å shortening of the ferryl bond, with an associated 44 cm^–1^ increase in calculated Fe–O stretching frequency, which correlates well with the 38 cm^–1^ increase observed experimentally (Figure S4).

**Figure 3:**
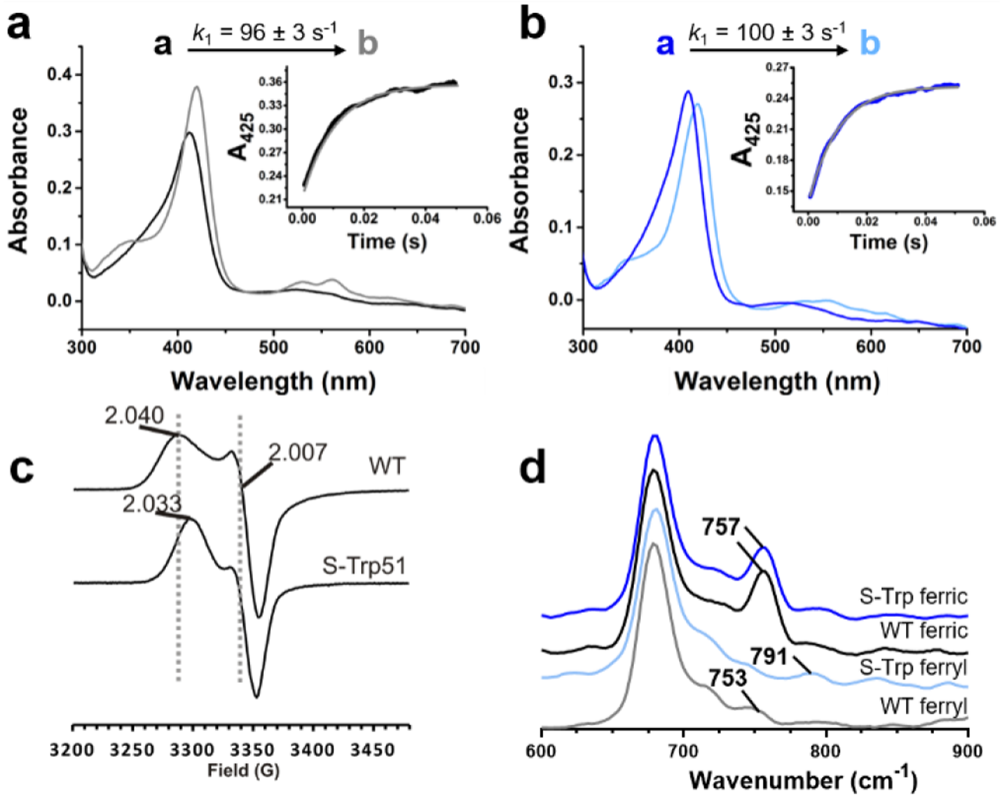
Spectroscopic characterization of the CpdI state of C*c*P and C*c*P S-Trp. (a,b) Oxidation of the ferric states of C*c*P (a, black line) and C*c*P S-Trp (b, dark blue) leads to the generation of a CpdI state with spectral features consistent with a neutral ferryl heme (gray line in C*c*P, pale blue line in C*c*P S-Trp, Soret maxima at 420 nm in both variants). Fitted transients are indicated in *insets*. (c) X-band continuous wave EPR spectra of the CpdI state of C*c*P and C*c*P S-Trp. EPR measurements are at 6 K, and *g* values are marked. (d) Raman spectra of the ferric and ferryl states of C*c*P (black and gray lines, respectively) and C*c*P S-Trp (dark blue and pale blue, respectively).

To investigate the effect of the Trp51S-Trp substitution on ferryl heme stability, stopped flow measurements were repeated over a longer time frame. In wild-type C*c*P, the neutral ferryl heme was stable for >5 min (Figure S5c). In contrast, time-resolved UV/vis spectra reveal that the ferryl heme of C*c*P S-Trp decays to the ferric state with rate of *k*_3_ = ∼ 0.04 s^–1^ (Figure S5a). To gain insights into the origins of this reduced ferryl heme lifetime, we replaced the redox active Trp191 with Phe in C*c*P and C*c*P S-Trp, which allows the CpdI and CpdII states to be differentiated spectroscopically (Figure 4a). Previous studies have demonstrated that oxidation of C*c*P W191F generates a classical CpdI porphyrin π-cation radical state typical of most peroxidases with spectral features distinct to neutral ferryl heme systems.^29,33^ Oxidation of W191F variants of C*c*P and C*c*P S-Trp with H_2_O_2_ (1.5 equiv) leads to the rapid (transient) formation of a CpdI porphyrin π-cation radical at similar rates in both variants (*k*_1_ = 126 ± 1 and 122 ± 1 s^–1^, respectively, Figure 4b,c, Figures S6 and S7), as indicated by a substantial decrease in the Soret intensity (maxima at 406 and 407 nm, respectively). These data provide further evidence that Trp191 is the site of radical formation in both WT and C*c*P S-Trp. The CpdI states of C*c*P W191F and C*c*P S-Trp W191F subsequently decay to a neutral ferryl heme (CpdII) with rates (*k*_2_) of 24.2 ± 0.1 s^–1^ and 6.93 ± 0.01 s^–1^, respectively, indicating that the S-Trp substitution stabilizes the CpdI porphyrin π-cation radical (Figure 4b,c, Figures S6 and S7). In contrast, the S-Trp substitution decreases the lifetime of the CpdII state, which is stable for >5 min in C*c*P W191F (*k*_3_ = ≪0.003 s^–1^) but decays with a rate of *k*_3_ = ∼ 0.07 s^–1^ in C*c*P S-Trp W191F (Figure 4d, Figure S5b,d). This is similar to the observed increased reactivity of the neutral ferryl heme state of C*c*P S-Trp vs wild-type C*c*P.

**Figure 4:**
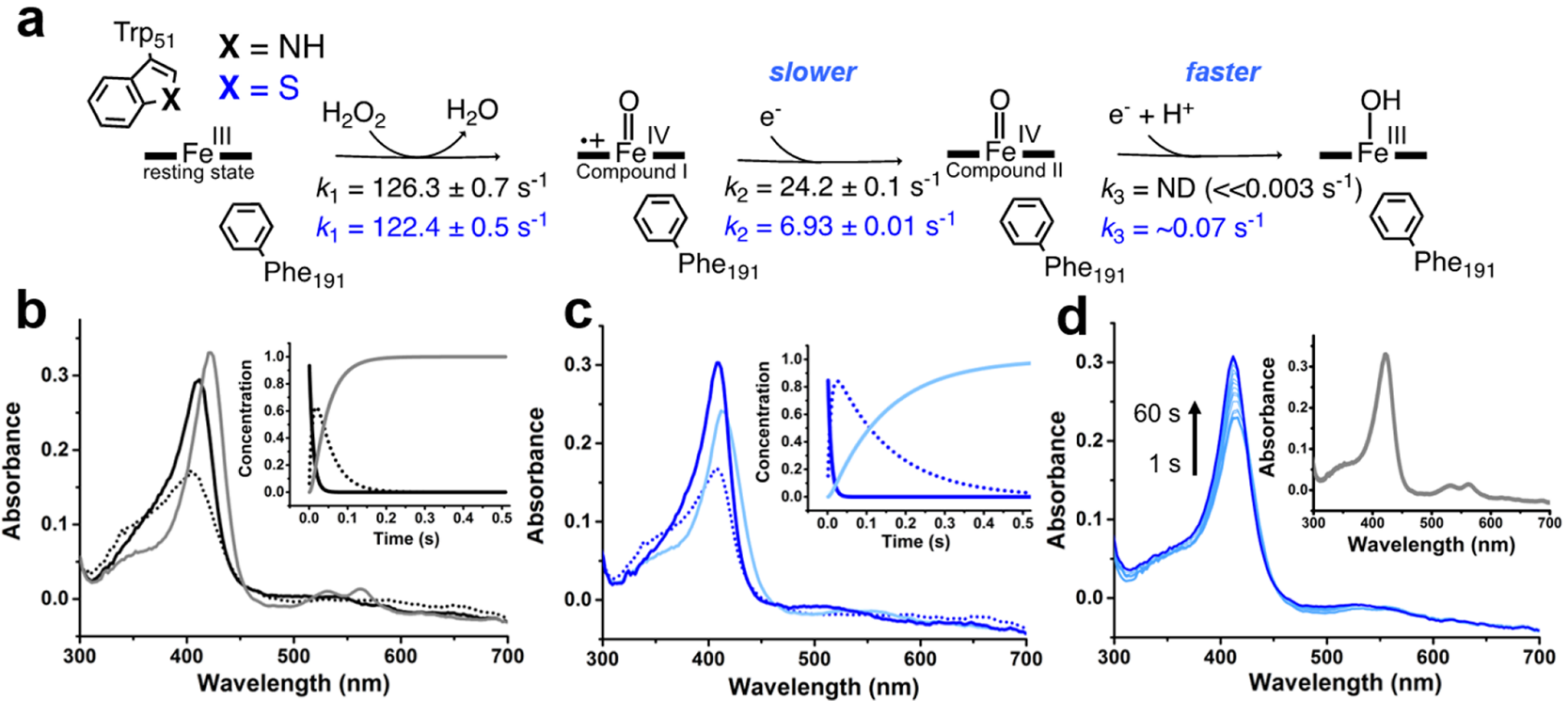
Stopped-flow analysis of intermediate species generated upon oxidation of C*c*P W191F and C*c*P S-Trp W191F. (a) The Trp51S-Trp substitution in C*c*P W191F stabilizes the CpdI state but leads to a faster decay of CpdII. (b,c) Oxidation of the ferric states of C*c*P W191F (a, black line) and C*c*P S-Trp W191F (b, dark blue line) leads to the generation of a transient porphyrin π-cation radical in both variants (dotted line), which decays to a CpdII state (gray line in C*c*P W191F (Soret maximum 423 nm) and pale blue line in C*c*P S-Trp W191F (Soret maximum 414 nm)). Concentration profiles are indicated in insets. (d) The CpdII state of C*c*P S-Trp W191F decays to the ferric enzyme (dark blue line) over 60 s, whereas CpdII of C*c*P W191F is stable for >5 min (inset).

To understand the contrasting impact of the S-Trp substitution on CpdI and CpdII reactivity in C*c*P W191F, active site DFT calculations employing the Gaussian 09 software package were used to explore cluster models (see SI for details), which were generated based on a previously reported C*c*P CpdI structure (PDB code: 5EJX).^17^ The calculations showed that replacement of Trp51 with S-Trp led to a modest 1.3 kcal mol^–1^ reduction in the calculated electron affinity of CpdI, in accordance with the slower rate of CpdI reduction observed experimentally with the C*c*P S-Trp W191F variant (Figure 4b). Our *in silico* results show that reduction of CpdII leads to spontaneous proton transfer from His52 (via an ordered water) to generate a ferric hydroxide state. Despite the increased reactivity of CpdII observed experimentally in C*c*P S-Trp variants, PCET to CpdII is thermodynamically less favorable in C*c*P S-Trp W191F (ΔΔ*G*_PCET_ = 3.6 kcalmol^–1^, Figure S9, Table S4). We considered the possibility that the increased rate of CpdII decay could be attributed to single electron oxidation or sulfoxidation of S-Trp51. However, these off-pathway processes were discounted as (1) *in silico* sulfoxidation of S-Trp by CpdI, and to a greater extent CpdII, is endothermic (Table S12) and (2) prior studies have shown S-Trp to be considerably more difficult to oxidize to the radical state than Trp.^34^ To investigate the origins of the increased CpdII reactivity, we instead elected to deconvolute the PCET process into the component electron and proton transfer steps. We first calculated diabatic electron affinities for CpdII (EA_II_), which show that S-Trp substitution leads to a substantial reduction in electron affinity (Δ*E*A_II_ = 10.0 kcal mol^–1^, Figure S9, Table S4). Similar Δ*E*A_II_ values were calculated for adiabatic CpdII reduction by placing restrictions on the N–H/O–H bonds of Arg48, His52, and the ordered water. In contrast, proton transfer to the reduced CpdII species (Δ*G*_H-transfer_ = Δ*G*_PCET_ – EA_II_) is 6.4 kcalmol^–1^ more favorable in C*c*P S-Trp W191F. Numerous studies have demonstrated how the kinetics of PCET and related H atom transfers to ferryl centers are dominated by the basicity of the ferryl-oxygen,^9,35−37^ and therefore we propose that the increased reactivity of CpdII observed experimentally upon S-Trp substitution can be ascribed to the substantial increase in Δ*G*_H-transfer_.

The combined experimental and computational data indicate that the Trp51 residue suppresses the reactivity and proton affinity of CpdII through hydrogen bonding to the ferryl oxygen. To understand the role of Trp51 in regulating the catalytic function of C*c*P, we determined the kinetic parameters for the oxidation of ferrous cyt*c*, and the nonbiological reductant guaiacol (*ortho*-methoxyphenol), by Trp51 and S-Trp51 variants of C*c*P and C*c*P W191F. The Trp51S-Trp substitution in C*c*P and C*c*P W191F causes dramatic 64-fold and 32-fold increases in the *k*_cat_ of guaiacol oxidation, respectively, with only modest changes in *K*_M_ (Figure 5, Figure S8a,b). This increased activity correlates well with the increased CpdII reactivity observed in the S-Trp containing variants. In contrast, the rate of cyt*c* oxidation is only modestly affected by the Trp51S-Trp substitution (*k*_cat_ = 819 ± 46 s^–1^ and 596 ± 33 s^–1^ for C*c*P and C*c*P S-Trp, respectively, Figure 5 and Figure S8c). As anticipated, replacement of the redox active Trp191 with Phe abolishes cyt*c* oxidation activity in C*c*P and C*c*P S-Trp.^33^

**Figure 5:**
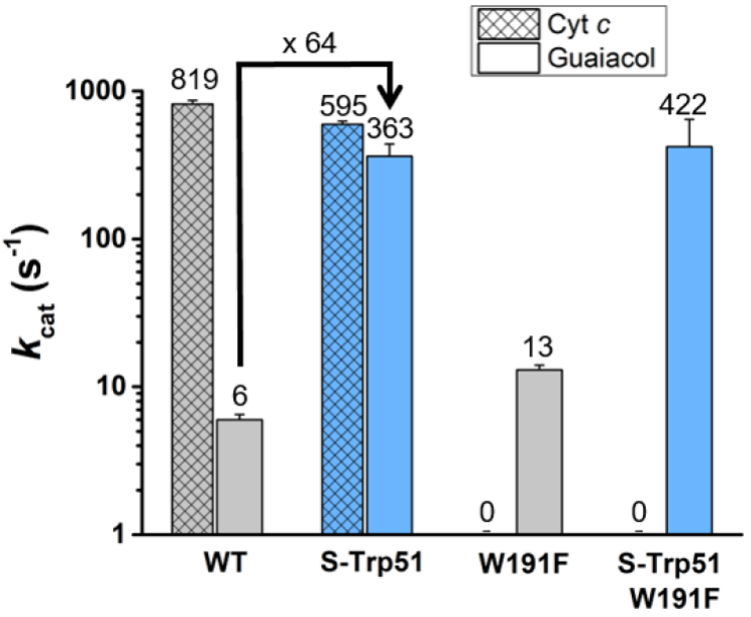
Kinetic characterization of C*c*P, C*c*P S-Trp, and their W191F variants. Bar chart showing the kinetics (*k*_cat_) of cyt*c* and guaiacol oxidation by wild-type C*c*P, C*c*P S-Trp, and their W191F variants.

Taken together, these data demonstrate how Trp51 tunes the lifetimes of key ferryl intermediates and works in synergy with the redox active Trp191 to control the substrate specificity of C*c*P. Specifically, hydrogen-bonding from Trp51 extends the lifetimes of neutral ferryl heme intermediates, such as CpdII and the CpdI ferryl heme-Trp191 radical pair, which is likely important for biological function.^40^ This study builds upon previous work to improve the reactivity of C*c*P toward small molecules by engineering binding sites for non-native substrates.^29,38^ What emerges is a complex picture whereby local ferryl coordination environments, the location and stability of key radical intermediates, and the presence of well-defined substrate binding sites work in synergy to control enzyme activity and selectivity. Nevertheless, the observation that Trp41Phe variants of APX (e.g., APEX2) also show increased activity with nonbiological substrates suggests that hydrogen-bonding to ferryl intermediates may control substrate specificity across multiple peroxidases.^10,25^ Further detailed studies will be needed to fully understand how high cyt*c* oxidation activity is achieved by the wild-type enzyme in spite of the reduced CpdII reactivity as a result of hydrogen bonding between Trp51 and the ferryl oxygen. Nevertheless, our data are consistent with a model where binding of the biological redox partner ferrous cyt*c* induces subtle long-range conformational changes that weaken the Trp51-ferryl oxygen hydrogen bond to trigger efficient proton-coupled electron transfer from cyt*c via* the redox active Trp191. Alternatively, substrate specificity for cyt*c* is achieved through tightly coupled proton and electron delivery to CpdII, which is perfectly tuned through evolution to minimize the barrier to PCET. These mechanistic hypotheses would explain why the Trp51S-Trp mutation has negligible impact on cyt*c* oxidation activity, but causes a large increase in nonbiological oxidations due to the formation of an inherently more reactive CpdII state.

This study illustrates how an expanded genetic code can provide new tools to study complex bioinorganic reaction mechanisms. Genetically encoded cysteine and histidine analogues have been used by our lab and others to probe the influence of proximal heme ligands on CpdI and CpdII reactivity.^9−12^ Here, we have employed a noncanonical Trp analogue to elucidate how an active site hydrogen bond regulates C*c*P function by modulating ferryl heme p*K*_a_ and reactivity. Proton-coupled electron transfers and related H atom transfers to high-energy metal-oxo intermediates are thought to be ubiquitous in biological systems.^17,35,39^ Consequently, we anticipate that the results presented will have wide-ranging implications for our understanding of metal-oxo reactivity in diverse enzyme active sites.

## Supporting Information Available

Materials and methods, calculations methods and data, MS data, UV–vis spectral data, kinetic analysis, structure statistics (PDF)

## Accession Codes

The crystal structures of C*c*P S-Trp and C*c*P S-Trp W191F were deposited in the RCSB Protein Data Bank (PDB) under accession numbers 6Y1T and 6Y2Y, respectively.
