# Supplementary material for: A Noncanonical Tryptophan Analogue Reveals an Active Site Hydrogen Bond Controlling Ferryl Reactivity in a Heme Peroxidase
Source: JACS Au. 2021 May 14;1(7):913–8. doi: 10.1021/jacsau.1c00145 (PMC8317151; doi:10.1021/jacsau.1c00145)
Supplement: Supplementary file 1 — au1c00145_si_001.pdf [file au1c00145_si_001.pdf]

Supporting Information

A non-canonical tryptophan analogue reveals an active site hydrogen bond controlling ferryl reactivity in a heme peroxidase

Mary Ortmayer,[1]‡ Florence J. Hardy,[1]‡ Matthew G. Quesne,[2,3] Karl Fisher,[1] Colin Levy,[1] Derren J. Heyes,[1] C. Richard A. Catlow,[2,3,4] Sam P. de Visser,[5] Stephen E. J. Rigby,[1] Sam Hay,[1] Anthony P. Green[1]*

[1]Department of Chemistry & Manchester Institute of Biotechnology, The University of Manchester, 131 Princess Street, Manchester, M1 7DN, U.K.

[2]Research Complex at Harwell, Rutherford Appleton Laboratory, Harwell Oxford, Didcot, Oxon, OX11 0FA, U.K.

[3]Cardiff University, School of Chemistry, Main Building, Park Place, Cardiff, CF10 3AT, U.K.

[4]Kathleen Lonsdale Materials Chemistry, Department of Chemistry, University College London, 20 Gordon Street, London WC1H 0AJ, U.K.

[5]Department of Chemical Engineering and Analytical Science & Manchester Institute of Biotechnology, The University of Manchester, 131 Princess Street, Manchester, M1 7DN, U.K.

*anthony.green@manchester.ac.uk

Methods

Materials

All materials were obtained from Sigma-Aldrich unless otherwise stated. Oligonucleotides were synthesized by IDT. Ferric cyt*c* from horse heart was obtained from Sigma-Aldrich and used throughout the study.

Construction of pEVOL_PylRS_S-Trp

Two mutations previously described mutations (N346G and C348Q) were introduced into the pBK-PylRS to generate pBKPylRS_S-Trp.[1] Mutations were introduced via assembly PCR. Fragments corresponding to the N and C termini were synthesized using the primer pairs PylRS_for and Maz_bta_dR, Maz_bta_dF and PylRS_rev (Table S1). The products of the two previous PCRs were purified and then combined in equimolar concentrations for final assembly PCR. The full-length fragment was amplified using the primers PylRS_for PylRS_rev (Table S1), digested by *Nde*I and *Pst*I and ligated into a pBK vector digested by the same restriction enzymes, yielding pBKPylRS_S-Trp. To construct pEVOL_PylRS_S-Trp, the same mutated fragment was digested by *Nde*I and *Pst*I and ligated into a pEVOL vector digested by the same restriction enzymes. Next, the PylRS_S-Trp gene was amplified using the primer pair pBK_PylRS_BglII and pBK_PylRS_SalI (Table S2), digested with *Bgl*II and *Sal*I and ligated into the pEVOL vector digested by the same restriction enzymes, yielding pEVOL_PylRS_S-Trp containing two copies of the PylRS(S-Trp) gene. DNA sequence was confirmed as described above using pBAD_for and pBAD_rev.

Construction of pET-11a_*Cc*P, pET-11a_*Cc*P_S-Trp and their variants

The gene encoding cytochrome *c* peroxidase (Ccp1p from the *S. cerevisiae* YJM1444 genome) was PCR amplified from plasmid pLeics03CCP.[2] using Phusion High-Fidelity DNA polymerase (New England BioLabs). Primers contained an N-terminal His_6 tag and complimentary sequences to allow insertion of the PCR product into pET-11a plasmid using In-Fusion® Advantage PCR Cloning via *Nde*I and *Bam*HI restriction sites, yielding pET-11a_*Cc*P. The Trp51S-Trp mutation was introduced into the pET-11a_*Cc*P by replacing the Trp51 codon with a TAG stop codon. Site-directed mutagenesis using the Q5® Site-Directed Mutagenesis Kit (New England BioLabs) of pET-11a_*Cc*P yielded pET-11a_*Cc*P_S-Trp. The W191F mutation was introduced into pET-11a_*Cc*P and pET-11a_*Cc*P_S-Trp using the Q5® Site-Directed Mutagenesis Kit. Correct DNA sequences were confirmed by DNA sequencing using T7 and T7_term primers (Source BioScience).

Protein production and purification

For expression of *Cc*P and its variants, pET-11a_*Cc*P and pET-11a_*Cc*P_W191F were transformed into BL21(DE3) *E. coli* and the cells were plated onto an LB agar (Formedium) plate containing 50 μg/ml ampicillin. A single colony of freshly transformed cells was cultured for 18 h in 10 ml of LB medium containing 50 μg/ml ampicillin. 4 ml of the culture was used to inoculate 400 ml of 2xYT medium (Formedium) supplemented with 1 mM δ-aminolevulinic acid and 50 μg/ml ampicillin. The culture was incubated for ~2 h at 37 °C with shaking at 180 rpm. When the OD_{600} of the culture reached ~0.5, IPTG was added to a final concentration of 100 μM. The induced cultures were incubated for ~20 h at 25 °C, and the cells were subsequently harvested by centrifugation at 7000 g for 10 min.

For expression of *Cc*P S-Trp and its variants, pET-11a_*Cc*P_S-Trp and pET-11a_*Cc*P_S-Trp _W191F were co-transformed into BL21(DE3) *E. coli* with pEVOL_$PylRS_{S\text{-}Trp}$ and the cells were plated onto an LB agar plate containing 50 μg/ml ampicillin and 25 μg/ml chloramphenicol. A single colony of freshly transformed cells was cultured for 18 h in 10 ml of LB medium containing 50 μg/ml ampicillin and 25 μg/ml chloramphenicol. 4 ml of the culture was used to inoculate 400 ml of 2xYT medium supplemented with 1 mM δ-aminolevulinic acid, 10 mM 3-benzothienyl-L-alanine (S-Trp) (Bachem), 50 μg/ml ampicillin and 25 μg/ml chloramphenicol. The culture was incubated for ~2.5 h at 37 °C with shaking at 180 rpm. When the OD_{600} of the culture reached ~0.5, IPTG and arabinose were added to a final concentration of 100 μM and 0.05%, respectively. The induced cultures were incubated for ~20 h at 25 °C, and the cells were subsequently harvested by centrifugation at 7000 g for 10 min.

The pelleted bacterial cells were suspended in phosphate buffer (50 mM KPi, 300 mM NaCl, 10 mM imidazole pH 7.5) supplemented with lysozyme (1 mg/ml), DNase (0.1 U/ml) and a Complete EDTA free protease inhibitor cocktail tablet (Roche) and subjected to sonication (13 mm probe, 15 mins, 20 s on, 40 s off, 40 % amplitude). Cell lysates were centrifuged at 27,000 *g* for 30 min and the supernatants subjected to affinity chromatography using Ni-NTA Agarose (Qiagen). His-tagged *Cc*P variants were eluted by 50 mM KPi, 300 mM NaCl, pH 7.5 buffer containing 300 mM imidazole. The purified protein was desalted using a 10DG desalting column (Bio-Rad) into 50 mM KPi pH 6 buffer. To maximize heme occupancy, variants were reconstituted with hemin chloride. Cell suspensions were mixed with 0.5 mM of hemin chloride (50 mM stock solution in 10 mM NaOH) for 30 mins at RT and filtered through a 0.45 micron membrane. The *Cc*P and *Cc*P S-Trp variants were then subjected to anion exchange chromatography with 50 mM KPi pH 6 buffer using a Resource™ Q column

on an AKTA Fast Protein Liquid Chromatography (FPLC) system (both GE Healthcare). Proteins were eluted by a linear gradient of NaCl in the concentration range 0-500 mM and the eluate was collected in 2 ml fractions. The purest fractions were selected after spectral analysis, pooled and concentrated using Vivaspin 20 centrifugal concentrators (Generon) with a 10 kDa molecular weight cut off membrane. The protein was aliquoted, flash frozen in liquid nitrogen and stored at -80 °C. The concentrations of C*c*P proteins were determined assuming an extinction coefficient of 101 $mM^{-1}cm^{-1}$ at 408 nm.[3] The heme occupancies of purified C*c*P His and C*c*P S-Trp and their variants are comparable, with similar Rz (A_{410}/A_{280}) values of ~1.5.

MS analysis

MS data for C*c*P and C*c*P S-Trp variants were acquired on a 1200series LC (Agilent). The final protein concentrations were adjusted to 10 µM in 50 mM KPi pH 6. 5 µL of sample was injected into 1 ml min^{-1} 5% acetonitrile (0.1% formic acid) and desalted inline. This was eluted over 1 minute by 95% acetonitrile. The resulting multiply charged spectrum was analysed by an QTOF 6510 (Agilent) in ESI positive ion mode, and deconvoluted using Masshunter Software (Agilent). The instrument had previously been tuned and calibrated with reference solution. All data is presented in Extended Data Fig. 1.

Crystallization, refinement and model building

C*c*P S-Trp and C*c*P S-Trp W191F were crystallized at 50 mg ml^{-1} in 50 mM KPi, pH 6 buffer. Initial crystallization conditions for C*c*P S-Trp and C*c*P S-Trp W191F were identified using the Morpheus and JCSG-plus screens (Molecular Dimensions), respectively. Crystals suitable for diffraction experiments were obtained by sitting drop vapor diffusion at 4 °C in 400 nl drops containing equal volumes of protein and crystallization solution. For C*c*P S-Trp this contained 60 mM magnesium chloride hexahydrate, 60 mM calcium chloride dihydrate, 0.1 M imidazole, 0.1 M MES pH 6.5, 20% v/v ethylene glycol and 10% w/v PEG 8000. For C*c*P S-Trp W191F this contained 0.1 M HEPES pH 7.5 and 10% w/v PEG 8000. The crystals were cryoprotected by the addition of 10% PEG 200 to the mother-liquor and flash-cooled in liquid nitrogen. Data were collected on beamline IO4 (wavelength 0.9795 Å and 0.9770 for C*c*P S-Trp and C*c*P S-Trp W191F, respectively) at the Diamond Light Source Facility and reduced and scaled with the X-ray Detector Software suite (XDS37). The C*c*P S-Trp and C*c*P S-Trp W191F crystal structures were determined by molecular replacement using the PHASER program in the CCP4 suite[4] using wild-type C*c*P structure as the starting model (PDB code: 2CYP)[5]. The C*c*P S-Trp and C*c*P S-Trp W191F models were completed by iterative cycles of manual model building and real space refinement using the program Coot and crystallographic refinement using Refmac. The processing and final refinement statistics are presented in Extended Data Table 1. The C*c*P S-Trp and C*c*P S-Trp W191F coordinates and structure factors have been deposited in the Protein Data Bank under accession number 6Y1T and 6Y2Y, respectively.

Calculations

All models started from heavy atom position taken from a crystal structure of the Compound I state of C*c*P (PDB code: 5EJX). Model systems of the two second coordination sphere of CpdI and CpdII were formed of: imidazole groups for His175 and His54, the carboxyl group of Asp235, truncated Arg48 and Phe191 minus the backbone atoms, one crystal water coordinating Fe-O and either a Trp51 or S-Trp51 residue. The terminal carbon atoms of each residue were frozen to simulate the effect

of the protein backbone, with full optimization being allowed in all other degrees of freedom. All energies reported here were obtained using a combination of the hybrid functional UB3LYP and the triplet-ζ basis set 6-311+G*[6],[7] on all atoms except for Fe where a combination of lanl2dz and an effective neon core-potential was applied. This methodology has been used extensively for calculating thermodynamic properties of group transfer reactions.[8]–[10] Analytical frequencies were run on each structure at 1 atm pressure and a temperature of 298.15 K and the resulting free energies form the basis of our thermodynamical analysis. These calculations also confirmed the presence of true local minima structures by the absence of any imaginary frequencies. Corrections for long range non-bonding interactions were given using the Grimme D3 dispersion model,[11] as implemented in the Gaussian09 software package.[12]

Spectroscopic characterization of C*c*P and C*c*P S-Trp variants

UV-vis absorption analysis was carried out on a Cary 50Bio UV-vis spectrophotometer (Varian), using a 1 cm path length quartz cuvette, recording spectra between 250 and 700 nm and typically with C*c*P at 4 μM in 50 mM KPi pH 6 buffer (See Extended Data Fig. 5).

Steady state enzyme kinetic assays

Steady state enzyme assays were carried out on a Cary 50Bio UV-vis spectrophotometer (Varian) in 50 mM KPi pH 6 using a 1 cm path length, 1 ml quartz cuvette. For cyt*c* enzyme assays C*c*P, C*c*P S-Trp, and C*c*P S-Trp W191F variants were diluted to 1.0 μM on the basis of the heme absorption, respectively. Cytc^{II} was prepared by reduction with dithionite and the excess reductant was removed with a 10DG desalting column. The concentration of cytc^{II} was calculated using the extinction coefficient; ε_{550} = 27.7 mM^{-1} cm^{-1}.[3] At each concentration of cyt*c* (0–150 μM), C*c*P and C*c*P S-Trp variants were diluted to 1 nM and H_2O_2 (100 μM) was added to initiate the reaction and the rate of change in absorbance at 550 nm was monitored over 1 min at 25 °C. The difference in absorptivity of cytc^{II} and cytc^{III} was calculated using $\Delta\varepsilon_{550}$= 19.5 mM^{-1} cm^{-1}.[13] Reactions were performed in triplicate (at RT) to produce a mean rate calculated as moles of cytc^{II} oxidised per mol C*c*P per second. These data were plotted against the relevant cytc^{II} concentration and fitted using the Michaelis–Menten hyperbolic function within Origin software. Reported values are corrected for background cyt*c* oxidation in the absence of enzyme. For C*c*P W191F and C*c*P S-Trp W191F (50 nM) no activity was observed above background.

For guaiacol enzyme assays C*c*P, C*c*P S-Trp and C*c*P S-Trp W191F variants were diluted to 1 μM on the basis of the heme absorption, respectively. At each concentration of guaiacol (0–150 mM), C*c*P, C*c*P S-Trp and C*c*P S-Trp W191F variants were diluted to 300, 10, and 10 nM respectively and 2.5 mM H_2O_2 was added to initiate the reaction. Product formation was monitored at 470 nm over 5 min at 25 °C and calculated using $\Delta\varepsilon_{470}$= 22.6 mM^{-1} cm^{-1}.[3] All reactions were performed in triplicate to produce a mean rate calculated as moles of substrate oxidized per mole C*c*P per second. These data were plotted against the relevant substrate concentration and fitted using the Michaelis–Menten hyperbolic with substrate inhibition or Hill functions within Origin software.

Electron paramagnetic resonance (EPR) analysis

Continuous wave EPR spectra were recorded at X-band (~ 9.4 GHz) using a Bruker ELEXSYS E500/ E580 EPR spectrometer (Bruker GmbH). Temperature was maintained using an Oxford Instruments ESR900 helium flow cryostat coupled to an

ITC 503 controller from the same manufacturer. EPR sample tubes were 4 mm Suprasil quartz supplied by Wilmad. CpdI was formed using 200 µM protein with 180 µM H_2O_2 in 50 mM KPi pH 6 buffer. EPR experiments employed 10 µW microwave power (non-saturating), 100 KHz modulation frequency and 1 G (0.1 mT) modulation amplitude to avoid diminution of any partially resolved hyperfine coupling.

Stopped-flow kinetics

Stopped-flow absorbance experiments were performed on an Applied Photophysics SX18 stopped-flow spectrophotometer (Applied Photophysics Ltd) equipped with a xenon arc lamp and a 1 cm path length in 50 mM KPi pH 6 buffer. To follow the spectra evolution of ferric C*c*P, C*c*P W191F, C*c*P S-Trp and C*c*P S-Trp W191F to CpdI intermediates, the drive syringes were loaded with separate solutions of 8 µM ferric protein and 12 µM H_2O_2. Multiple wavelength data were collected at RT using a (PDA) detector and XSCAN software.

Resonance Raman spectroscopy

Resonance Raman spectra were measured using an InVia Raman spectrometer (inVia 286A51, Renishaw Plc., UK) equipped with a 405 nm diode laser and 2400 l/mm gratings. Samples contained 100 µM protein (either wild type or variant) and were loaded (20 µl) into a 100 mm soda glass capillary (G119/02, Thermo Fisher Scientific, USA). The ferryl species was prepared by rapidly mixing (<10 s mixing time) the protein with a 10-fold excess of H_2O_2 prior to the measurement at 4°C. The experimental parameters used for the measurements were 100% laser power (~10 mW), x50 objective lens, 1 s exposure time and 100 spectral accumulations for the resting enzyme or 10 accumulations for the ferryl species. Raman spectra were baseline corrected and normalized for ease of comparison.
These results were compared to *in silico* modelling of analytical frequencies with all degrees of freedom allowed and where second derivative calculations were run at 1 atm of pressure and 395.15 K temperature. A Gaussian broadening protocol was then followed to produce the simulated spectra shown in Figure S4.

Table S1. Primers used in this study

PylRS_for	GAGGAATCCCATATGATGGATAAAA
PylRS_rev	CGTTTGAAACTGCAGTTACAGGTT
Maz_bta_dF	GTTTACCATGCTGggCTTTcagCAAATGGGTTCAG
Maz_bta_dR	CTGAACCCATTTGctgAAAGccCAGCATGGTAAAC
pBK_PylRS_BglII	gatcgatcAGATCTATGGACAAAAAACCGC
pBK_PylRS_SalI	gatcgatcGTCGACTTACAGGTTCGTAGAG

Table S2. Data collection, phasing and refinement statistics for C*c*P S-Trp51 and C*c*P S-Trp51 W191F

	C*c*P S-Trp	C*c*P S-Trp W191F
PDB code	6Y1T	6Y2Y
**Data collection**		
Space group	$P\ 2_1\ 2_1\ 2_1$	$P\ 2_1\ 2_1\ 2_1$
Cell dimensions		
a, b, c (Å)	50.85, 75.19, 106.98	50.97, 74.1, 106.41
α, β, γ (°)	90, 90, 90	90, 90, 90
Resolution (Å)	35.47 - 1.50 (1.55 - 1.50)[a]	43.22 - 1.70 (1.76 - 1.70)
R_{meas}	0.09279 (0.6033)	0.0641 (0.1918)
I/σI	9.07 (1.10)	19.36 (7.24)
Completeness (%)	0.99 (0.92)	1.00 (1.00)
Multiplicity	5.5 (2.8)	5.9 (5.0)
**Refinement**		
Resolution (Å)	35.47 - 1.50 (1.52 - 1.50)	43.22 - 1.70 (1.74 - 1.70)
Number of reflections	65800 (2251)	45042 (2618)
R_{work}/ R_{free}[b]	0.1467/ 0.1766 (0.2656)/ (0.2808)	0.1475/ 0.1801 (0.1473)/ (0.2378)
Number of atoms	2888	2744
B-factors (Å^2)	21.41	20.50
RMS deviations[c]		
Bond lengths (Å)	0.008	0.009
Bond angles (°)	1.01	0.90

[a]Values in parentheses are for highest resolution shell. [b]Rfree was calculated using 5% of the data separate from the rest of the reflections. [c]Root mean square (RMS) deviation from the parameter set for ideal stereochemistry.

Table S3. Mass spectrometry data for C*c*P, C*c*P S-Trp and their W191F variants

Variant	Expected Mass	Observed
C*c*P	34187	34187
C*c*P S-Trp51	34205	34205
C*c*P W191F	34148	34149
C*c*P S-Trp51 W191F	34166	34166

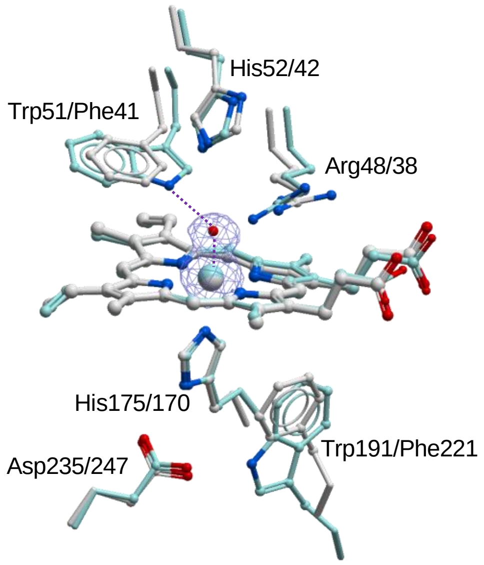

Figure S1. Overlay of CpdI structures of C*c*P and horseradish peroxidase. CpdI structures of C*c*P (cyan [PDB code: 3M23][14]) and horseradish peroxidase (white [PDB code: 1HCH][15]) are illustrated. The ferryl oxygen is shown for C*c*P and the $2F_o - F_c$ electron density map corresponding to the Fe^{IV}=O bond is contoured at 1σ (blue mesh).

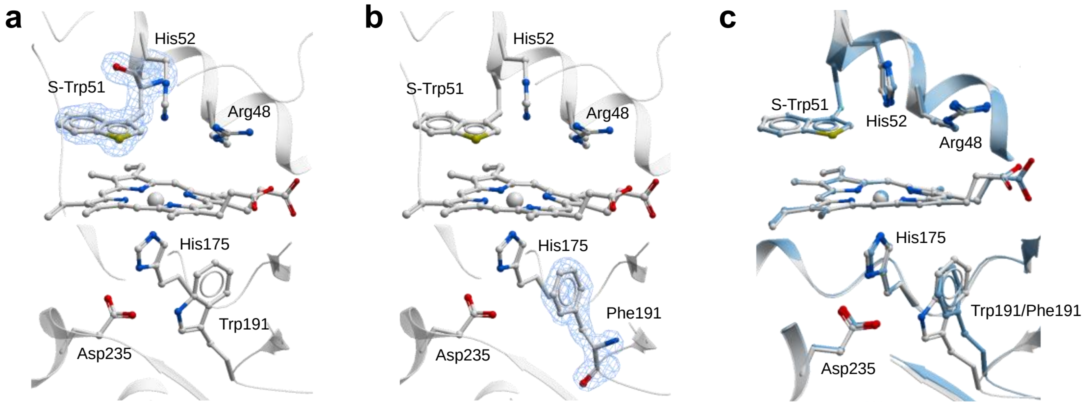

Figure S2. Detailed view and overlay of C*c*P S-Trp and C*c*P S-Trp W191F active sites. C*c*P S-Trp (a) and C*c*P S-Trp W191F (b) active site residues including S-Trp51 are shown as atom colored ball and sticks with white carbons. The $2F_o - F_c$ electron density map corresponding to the S-Trp51 / Phe191 residues is contoured at 1σ (blue mesh). c) Overlay of C*c*P S-Trp (PDB code: 6Y1T, white) and C*c*P S-Trp W191F (PDB code: 6Y2Y, blue) active sites with secondary structure superpose RMS deviation of 0.17 Å.

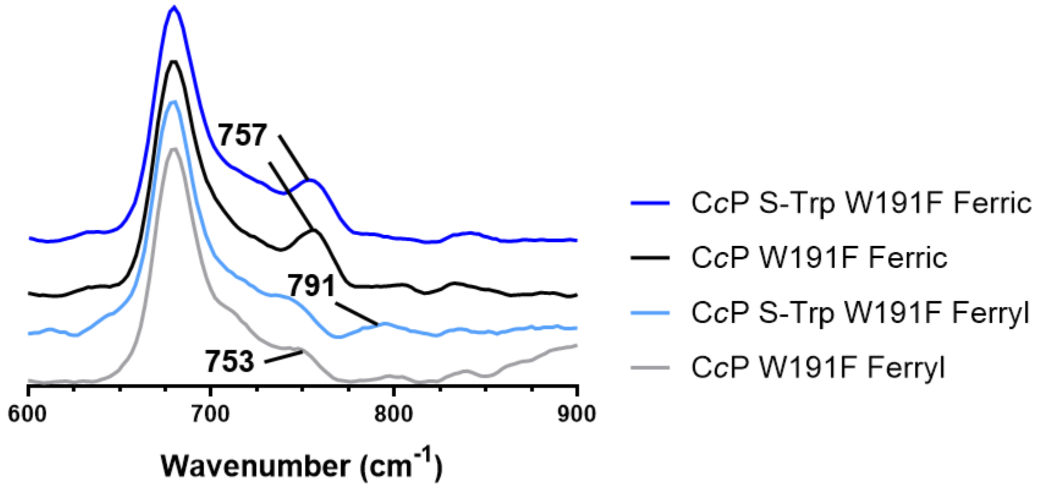

Figure S3. Resonance Raman spectra of C*c*P W191F and C*c*P S-Trp W191F. Resonance Raman spectra of C*c*P W191F in the ferric (black) and ferryl (grey) states, and of C*c*P S-Trp W191F in the resting (dark blue) and ferryl (light blue) states. Ferric state spectra were recorded at pH = 6, [enzyme] = 100 μM. Ferryl states of each variant were recorded after mixing to give a final $[H_2O_2]$ = 1 mM.

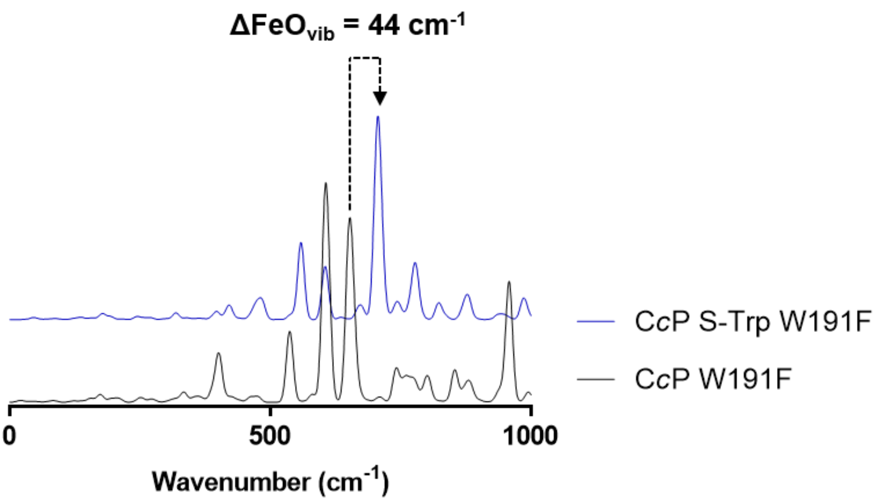

Figure S4. Simulated analytical frequency spectra of the CpdII states of C*c*P W191F and C*c*P S-Trp W191F. Vibrational frequencies for the CpdII states of C*c*P W191F (black) and C*c*P S-Trp W191F (blue) are both shown with the peak responsible for the Fe-O bond stretches annotated, at 659 cm^{-1} and 703 cm^{-1}, respectively. The calculated Fe-O bond lengths are 1.73 Å and 1.71 Å for the CpdII states of C*c*P W191F and C*c*P S-Trp W191F, respectively. An illustration of both models shown in Fig. S10b and S10e.

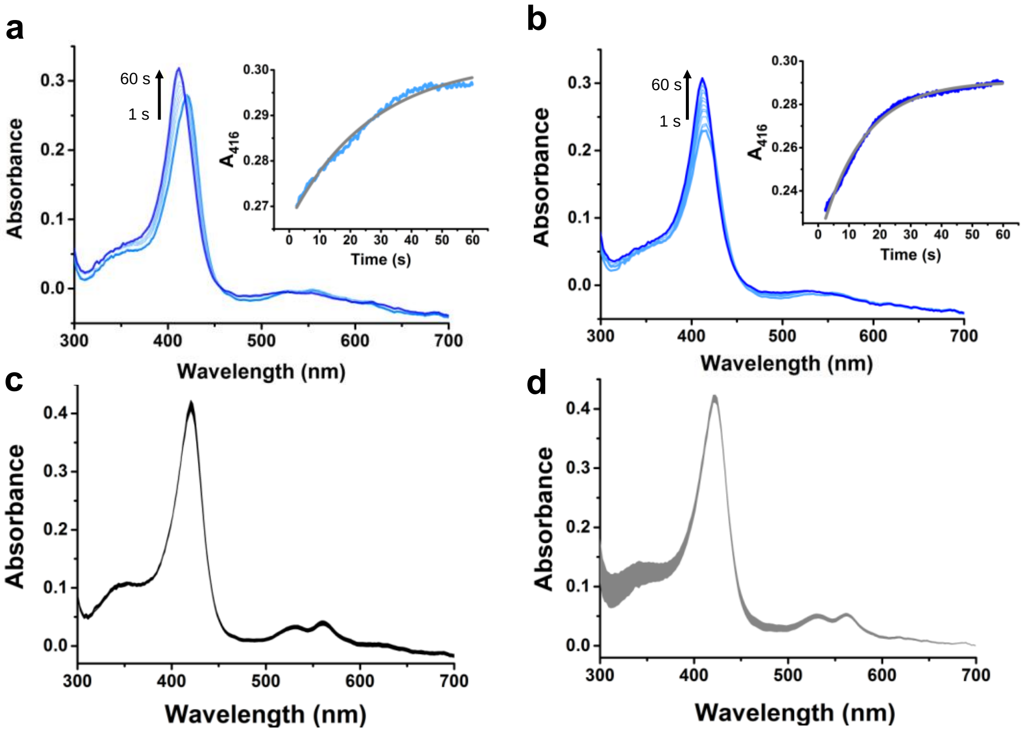

Figure S5. Spectroscopic characterization of ferryl intermediates of C*c*P, C*c*P S-Trp, C*c*P W191F and C*c*P S-Trp W191F. a) Spectra showing decay of ferryl intermediates to ferric resting state (navy blue line) of C*c*P S-Trp and b) C*c*P S-Trp W191F. *Insets* show time-dependent changes fitted to an a → b model to derive observed rate constants of k_3 = ~0.04 s^{-1} and k_3 = ~0.07 s^{-1} for C*c*P S-Trp and C*c*P S-Trp W191F, respectively. c-d) Spectra of ferryl stability over 5 min for C*c*P (c) and

C*c*P W191F (d). (a-d) Post-mixing conditions at pH 6 [enzyme] = 4 μM, [H_2O_2] = 6 μM.

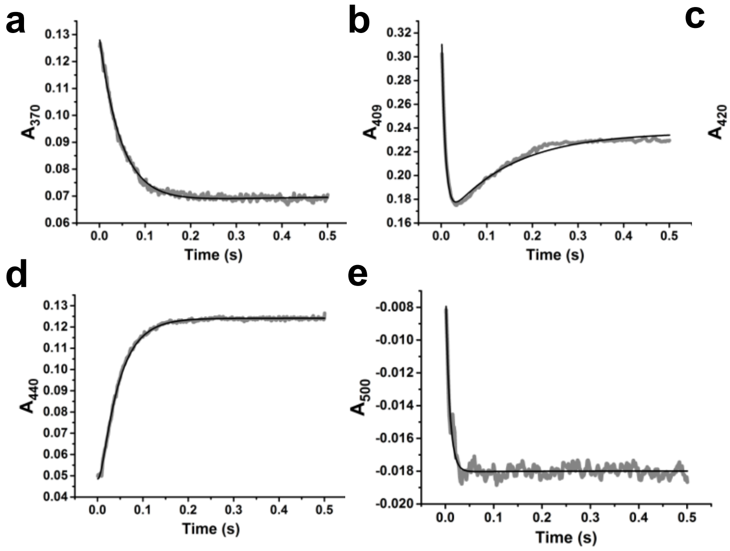

Figure S6. Kinetic transients showing CpdI and CpdII formation in C*c*P W191F Time-dependent changes were fitted to an a ➔ b ➔ c global analysis model to give observed rate constants of k_1 = 126.3 ± 0.7 s^{-1} and k_2 = 24.2 ± 0.1 s^{-1}. Kinetic transients are shown at selected wavelengths with fits derived from global analysis (black lines).

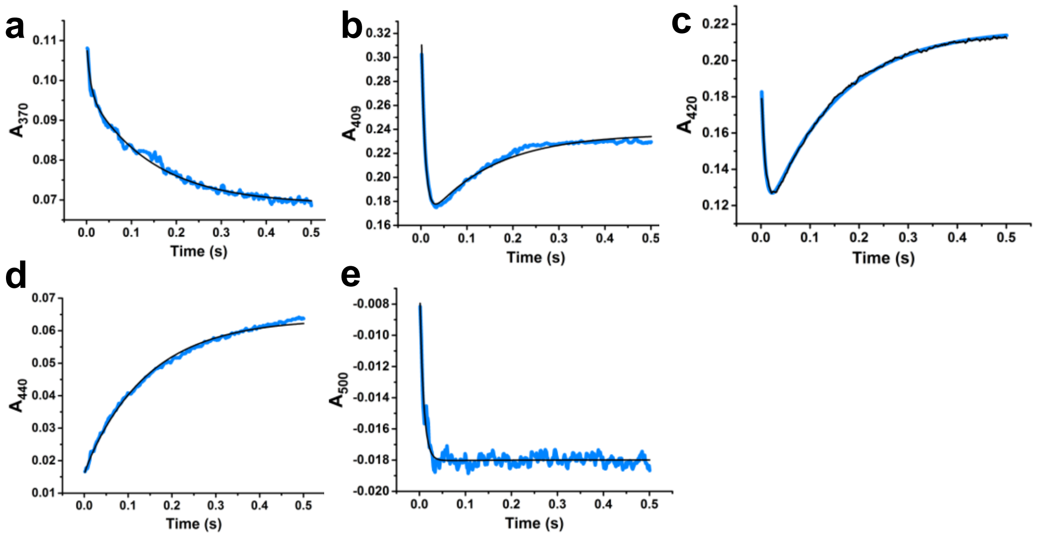

Figure S7. Kinetic transients showing CpdI and CpdII formation in C*c*P S-Trp W191F Time-dependent changes were fitted to an a ➔ b ➔ c global analysis model to give observed rate constants of k_1 = 122.4 ± 0.5 s^{-1} and k_2 = 6.93 ± 0.01 s^{-1} . Kinetic transients are shown at selected wavelengths with fits derived from global analysis (black lines).

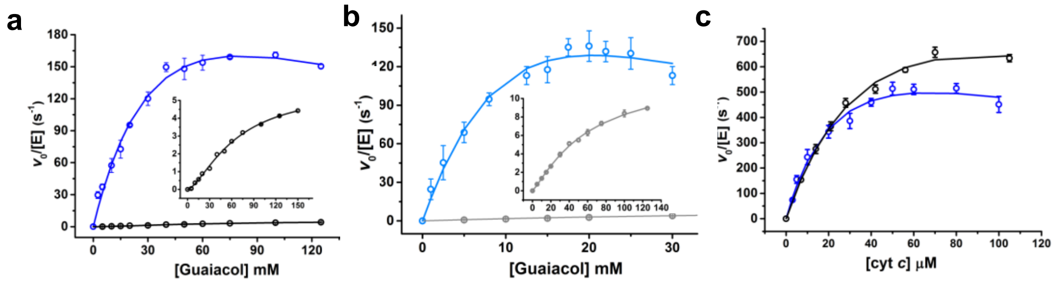

Figure S8. Hill and Michaelis-Menten plots of guaiacol and cyt*c* oxidation by C*c*P, C*c*P S-Trp, C*c*P W191F and C*c*P S-Trp W191F. (a) Michaelis-Menten with substrate inhibition plot of guaiacol oxidation by C*c*P S-Trp51 (blue, k_{cat} = 363 ± 76 s^{-1}, K_M = 52 ± 15 mM, k_i = 128 ± 57) and Hill plot for WT C*c*P guaiacol oxidation (*inset* shows full curve) (black, 5.7 ± 0.3 s^{-1} and K_M = 65.6 ± 6.2 mM *n*=1.5). b) Hill plot for C*c*P W191F guaiacol oxidation (grey line, *inset* shows full curve, k_{cat} = 13 ± 1 s^{-1}, K_M = 60 ± 6 mM, *n*=1.2) and Michaelis-Menten with substrate inhibition plot for C*c*P S-Trp W191F guaiacol oxidation (pale blue line, k_{cat} = 422 ± 222 s^{-1}, K_M = 23 ± 16 mM, k_i = 18 ± 15). (c) Michaelis-Menten plot for C*c*P cyt*c* oxidation (black, k_{cat} = 819 ± 46 s^{-1}, K_M = 25 ± 2 μM) and C*c*P S-Trp cyt*c* oxidation (blue, k_{cat} = 595 ± 33 s^{-1}, K_M = 14 ± 3 μM). Measurements at pH 6, 25°C, error bars are SEM *n*=3.

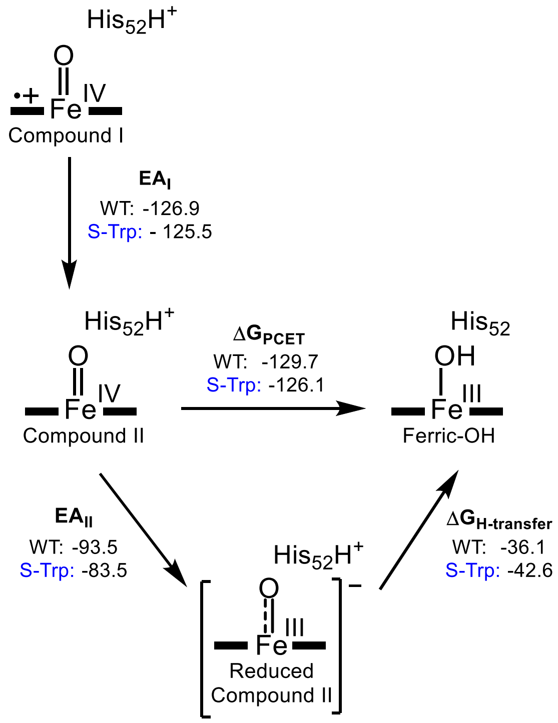

Figure S9. *In Silico* modelling. The *in silico* modelled species and the associated Gibbs free energy changes for each of the catalytic steps, values given in kcalmol^{-1}. EA_I: the free energy change upon adiabatic reduction of CpdI to CpdII. EA_{II}: the free energy change upon diabatic reduction of CpdII to the species reduced CpdII. ΔG_{PCET}: the free energy change upon PCET to CpdII. $\Delta G_{H\text{-transfer}}$: the free energy change upon proton transfer from distal pocket residue His52 to reduced CpdII.

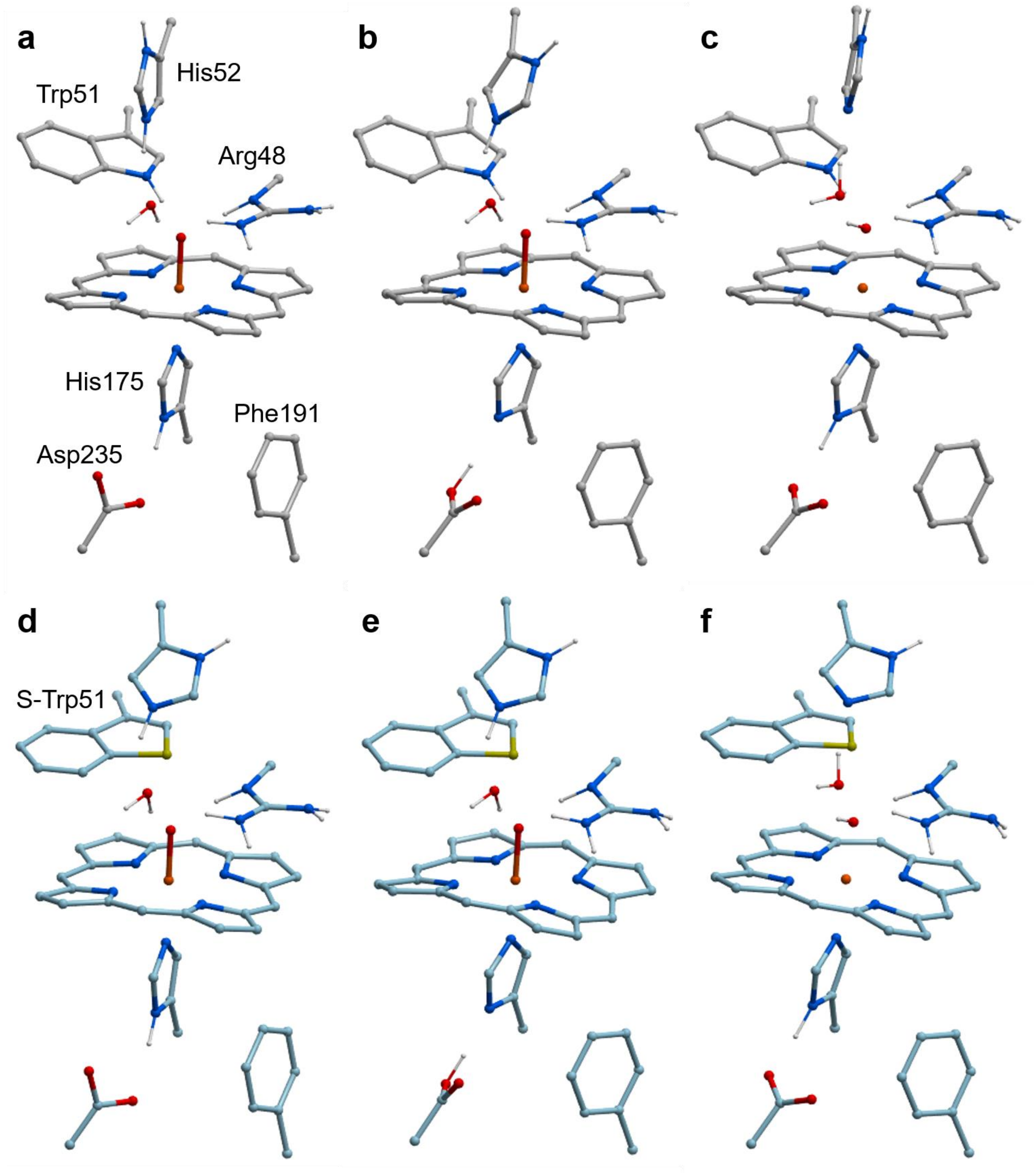

Figure S10. Optimised geometries of *in silico* modelled species. a) C*c*P W191F CpdI b) C*c*P W191F CpdII c) C*c*P W191F Ferric-OH d) C*c*P S-Trp W191F CpdI e) C*c*P S-Trp W191F CpdII f) C*c*P S-Trp W191F Ferric-OH

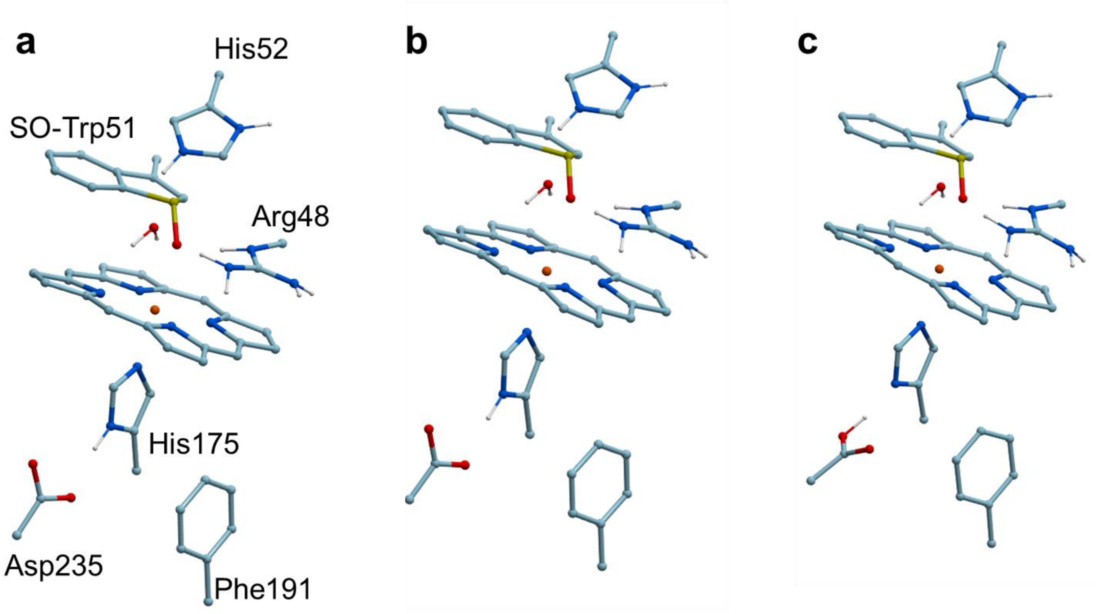

Figure S11. Optimised geometries of *in silico* modelled S-Trp sulfoxidation products a) Doublet spin state derived from CpdI b) Quartet spin state derived from CpdI c) Triplet spin state derived from CpdII.

Table S4. Free energy changes for EA_I, EA_{II}, ΔG_{PCET}, and $\Delta G_{H\text{-}transfer}$. Calculated thermodynamic values in kcalmol^{-1}. The values for EA_{II} are given for both diabatic electron transfer to CpdII and adiabatic electron transfer with restrictions placed on N-H/O-H bonds of Arg48, His52, and the ordered H_2O.

	EA_I	$EA_{II(diabatic)}$	$EA_{II(adiabatic)}$	ΔG_{PCET}	$\Delta G_{H\text{-}transfer}$
**C*c*P W191F**	-126.9	-93.5	-97.8	-129.7	-36.1
**C*c*P S-Trp W191F**	-125.5	-83.5	-87.3	-126.1	-42.6
**ΔΔG**	1.3	10.0	10.4	3.6	-6.4

Table S5. Total energy in Au for *in silico* models of CpdI. Energies for both C*c*P W191F and C*c*P S-Trp W191F are given for both the doublet and quartet spin states.

	E [au] B3LYP/BS2	E_{Zero} [au] B3LYP/BS2	E_{gibbs} [au] B3LYP/BS2	E_{sol} B3LYP/BS2
^{2}C*c*P W191F	-2943.576	0.956	-2942.721	-2943.768
^{2}C*c*P S-Trp W191F	-3286.404	0.941	-3285.564	-3286.595
^{4}C*c*P W191F	-2943.576	0.957	-2942.721	-2943.768
^{4}C*c*P S-Trp W191F	-3286.404	0.941	-3285.565	-3286.595

Table S6. Total energy in Au for *in silico* models of CpdII. Energies for both C*c*P W191F and C*c*P S-Trp W191F are given for both the triplet and quintet spin states.

	E [au] B3LYP/BS2	E_{Zero} [au] B3LYP/BS2	E_{gibbs} [au] B3LYP/BS2	E_{sol} B3LYP/BS2
^{3}C*c*P W191F	-2943.870	0.955	-2943.014	-2943.971
^{3}C*c*P S-Trp W191F	-3286.695	0.940	-3285.851	-3286.799
^{5}C*c*P W191F	-2943.825	0.953	-2942.971	-2943.930
^{5}C*c*P S-Trp W191F	-3286.669	0.938	-3285.831	-3286.772

Table S7. Total energy in Au for *in silico* models of reduced CpdII. Energies for both C*c*P W191F and C*c*P S-Trp W191F are given for both the doublet and quartet spin states. To determine $E_{sol(adiabatic)}$, restrictions were placed on N-H/O-H bonds of Arg48, His52, and the ordered water to prevent barrier-less proton transfer to the FeO centre. $E_{(diabatic)}$ values state the energies of the species after diabatic electron transfer to CpdII.

	**E [au]**	**E_{Zero} [au]**	**E_{gibbs} [au]**	**$E_{sol(adiabatic)}$**	**$E_{(diabatic)}$**
	**B3LYP/BS2**	**B3LYP/BS2**	**B3LYP/BS2**	**B3LYP/BS2**	
^{2}C*c*P W191F	-2944.068	0.952	-2943.211	-2944.128	-2944.120
^{2}C*c*P S-Trp W191F	-3286.878	0.937	-3286.033	-3286.939	-3286.932
^{4}C*c*P W191F	-2944.011	0.948	-2943.158	-2944.068	-2944.076
^{4}C*c*P S-Trp W191F	-3286.822	0.932	-3285.986	-3286.881	-3286.925

Table S8. Total energy in Au for *in silico* models of Ferric-OH. Energies for both C*c*P W191F and C*c*P S-Trp W191F are given for both the doublet and quartet spin states.

	**E [au]**	**E_{Zero} [au]**	**E_{gibbs} [au]**	**E_{sol}**
	**B3LYP/BS2**	**B3LYP/BS2**	**B3LYP/BS2**	**B3LYP/BS2**
^{2}C*c*P W191F	-2944.114	0.954	-2943.256	-2944.179
^{2}C*c*P S-Trp W191F	-3286.938	0.940	-3286.095	-3286.999
^{4}C*c*P W191F	-2944.086	0.952	-2943.232	-2944.151
^{4}C*c*P S-Trp W191F	-3286.911	0.935	-3286.073	-3286.975

Table S9. Grouped Mulliken spin densities for *in silico* models of Compound I, Compound II, reduced Compound II, and Ferric-OH for both C*c*P W191F and C*c*P S-Trp W191F. Spin populations are grouped by residue, heme co-factor, iron cofactor (Fe), oxo group (O), and the ordered water (H_2O).

	**Arg_{48}**	**Trp_{51}**	**His_{175}**	**Phe_{191}**	**Asp_{235}**	**Heme**	**Fe**	**O**	**H_2O**	**His_{52}**
CpdI ^{2}C*c*P W191F	0.01	-0.04	-0.09	-0.02	-0.93	-0.66	2.33	0.40	0.01	0.00
CpdI ^{2}C*c*P S-Trp W191F	0.00	0.00	-0.12	-0.02	-0.97	-0.59	2.16	0.53	0.01	0.00
CpdI ^{4}C*c*P W191F	0.01	0.05	-0.09	0.01	0.93	-0.65	2.33	0.40	0.01	0.00
CpdI ^{4}C*c*P S-Trp W191F	0.00	0.00	-0.11	0.02	0.97	-0.59	2.16	0.53	0.01	0.00
CpdII ^{3}C*c*P W191F	0.01	0.00	0.11	0.01	0.00	-0.71	2.32	0.25	0.01	0.00
CpdII ^{3}C*c*P S-Trp W191F	0.01	0.00	-0.01	0.00	0.00	-0.69	2.28	0.40	0.01	0.00
CpdII ^{5}C*c*P W191F	0.00	-0.01	-0.06	0.00	0.00	-0.45	4.12	0.40	0.00	0.00
CpdII ^{5}C*c*P S-Trp W191F	0.00	-0.01	-0.08	0.00	0.00	-0.61	4.40	0.29	0.01	0.00
Reduced CpdII ^{2}C*c*P W191F	0.00	0.00	0.00	0.00	0.00	-0.46	1.51	-0.04	0.00	0.00
Reduced CpdII ^{2}C*c*P S-Trp W191F	0.00	0.00	0.00	0.00	0.00	-0.43	1.47	-0.03	0.00	0.00
Reduced CpdII ^{4}C*c*P W191F	0.00	0.00	0.09	0.00	0.00	-0.95	3.91	-0.04	0.00	0.00
Reduced CpdII ^{4}C*c*P S-Trp W191F	0.00	-0.01	0.10	0.00	0.00	-0.94	3.89	-0.04	0.00	0.00
Ferric-OH ^{2}C*c*P W191F	0.00	0.00	-0.06	0.00	0.00	-0.48	1.56	-0.02	0.00	0.00
Ferric-OH ^{2}C*c*P S-Trp W191F	0.00	0.00	-0.06	0.00	0.00	-0.46	1.52	0.00	0.00	0.00
Ferric-OH ^{4}C*c*P W191F	0.00	0.00	-0.12	0.00	0.00	-0.60	3.84	-0.11	0.00	0.00
Ferric-OH ^{4}C*c*P S-Trp W191F	0.00	0.00	-0.11	0.00	0.00	-0.58	3.78	-0.09	0.00	0.00

Table S10. Grouped Mulliken charges for *in silico* models of Compound I, Compound II, reduced Compound II, and Ferric-OH for both C*c*P W191F and C*c*P S-Trp W191F. Spin populations are grouped by residue, heme co-factor, iron cofactor (Fe), oxo group (O), and the ordered water (H_2O).

	**Arg_{48}**	**Trp_{51}**	**His_{175}**	**Phe_{191}**	**Asp_{235}**	**Heme**	**Fe**	**O**	**H_2O**	**His_{52}**
CpdI ^{2}C*c*P W191F	1.13	0.26	2.58	0.14	0.02	10.94	-15.40	1.19	0.17	0.97
CpdI ^{2}C*c*P S-Trp W191F	1.01	0.08	3.01	0.15	0.05	11.69	-16.40	1.14	0.13	1.13
CpdI ^{4}C*c*P W191F	1.13	0.26	2.58	0.13	0.02	10.95	-15.40	1.19	0.17	0.97
CpdI ^{4}C*c*P S-Trp W191F	1.01	0.08	3.01	0.15	0.05	11.69	-16.40	1.14	0.13	1.13
CpdII ^{3}C*c*P W191F	1.04	0.10	1.26	0.10	0.01	10.74	-14.80	1.34	0.18	1.04
CpdII ^{3}C*c*P S-Trp W191F	1.00	0.08	1.81	0.11	-0.01	11.82	-16.45	1.42	0.13	1.08
CpdII ^{5}C*c*P W191F	1.09	0.00	1.56	0.08	0.01	9.71	-13.66	1.10	0.07	1.04
CpdII ^{5}C*c*P S-Trp W191F	0.93	0.11	2.09	0.11	0.02	12.30	-17.35	1.54	0.12	1.12
Reduced CpdII ^{2}C*c*P W191F	0.09	0.06	1.63	0.10	-0.01	9.91	-13.33	1.37	0.02	0.16
Reduced CpdII ^{2}C*c*P S-Trp W191F	0.09	0.00	1.40	0.13	-0.03	9.75	-12.89	1.49	-0.06	0.12
Reduced CpdII ^{4}C*c*P W191F	0.02	0.00	2.04	0.07	-0.04	10.00	-13.11	0.98	-0.06	0.09
Reduced CpdII ^{4}C*c*P S-Trp W191F	-0.03	0.00	2.33	0.07	-0.03	10.22	-13.67	0.92	-0.02	0.20
Ferric-OH ^{2}C*c*P W191F	0.99	0.05	2.28	0.08	-0.81	10.33	-14.13	1.08	0.02	0.12
Ferric-OH ^{2}C*c*P S-Trp W191F	0.98	0.05	2.39	0.06	-0.77	10.36	-14.30	1.14	-0.04	0.13
Ferric-OH ^{4}C*c*P W191F	0.94	0.03	2.32	0.06	-0.81	9.90	-13.60	1.04	0.00	0.12
Ferric-OH ^{4}C*c*P S-Trp W191F	0.84	0.02	2.22	0.05	-0.81	9.90	-13.39	1.07	-0.04	0.15

Table S11. Total energy in Au for *in silico* models of S-Trp sulfoxidation products derived from CpdI ([a]) or CpdII ([b]). The energies of products derived from CpdI are given for both the doublet and quartet states. The energies of products derived from CpdII are given for the triplet state.

	**E [au]**	**E_{Zero} [au]**	**E_{gibbs} [au]**
	**B3LYP/BS2**	**B3LYP/BS2**	**B3LYP/BS2**
^{2}C*c*P SO-Trp W191F[a]	-3286.385	0.943	-3285.537
^{4}C*c*P SO-Trp W191F[a]	-3286.382	0.941	-3285.540
^{3}C*c*P SO-Trp W191F[b]	-3286.644	0.940	-3285.800

Table S12. Calculated energy changes in kcalmol^{-1} for S-Trp sulfoxidation by CpdI ([a]) and CpdII ([b]). Energy changes are calculated from the lower energy spin states of CpdI and CpdII (Tables S5 and S6).

	**ΔE [kcal/mol]**	**ΔE_{Zero} [kcal/mol]**	**ΔE_{gibbs} [kcal/mol]**
	**B3LYP/BS2**	**B3LYP/BS2**	**B3LYP/BS2**
^{2}C*c*P S-Trp W191F[a]	11.98	12.88	17.12
^{4}C*c*P S-Trp W191F[a]	13.56	13.75	15.70
^{3}C*c*P S-Trp W191F[b]	31.63	31.91	32.30

References

[1] Englert, M.; Nakamura, A.; Wang, Y. S.; Eiler, D.; Söll, D.; Guo, L. T. Probing the Active Site Tryptophan of Staphylococcus Aureus Thioredoxin with an Analog. *Nucleic Acids Res.* **2015**, *43* (22), 11061–11067.

[2] Gumiero, A.; Metcalfe, C. L.; Pearson, A. R.; Raven, E. L.; Moody, P. C. E. Nature of the Ferryl Heme in Compounds I and II. *J. Biol. Chem.* **2011**, *286* (2), 1260–1268.

[3] Murphy, E. J.; Metcalfe, C. L.; Basran, J.; Moody, P. C. E.; Raven, E. L. Engineering the Substrate Specificity and Reactivity of a Heme Protein: Creation of an Ascorbate Binding Site in Cytochrome c Peroxidase. *Biochemistry* **2008**, *47* (52), 13933–13941.

[4] McCoy, A. J.; Grosse-Kunstleve, R. W.; Adams, P. D.; Winn, M. D.; Storoni, L. C.; Read, R. J. Phaser Crystallographic Software. *J. Appl. Crystallogr.* **2007**, *40* (4), 658–674.

[5] Finzel, B. C.; Poulos, T. L.; Kraut, J. Crystal Structure of Yeast Cytochrome c Peroxidase Refined at 1.7-Å Resolution. *J. Biol. Chem.* **1984**, *259* (21), 13027–13036.

[6] Becke, A. D. Density-Functional Thermochemistry. III. The Role of Exact Exchange. *J. Chem. Phys.* **1993**, *98* (7), 5648–5652.

[7] Lee, C.; Yang, W.; Parr, R. G. Development of the Colle-Salvetti Correlation-Energy Formula into a Functional of the Electron Density. *Phys. Rev. B* **1988**, *37* (2), 785.

[8] Sahu, S.; Quesne, M. G.; Davies, C. G.; Dürr, M.; Ivanović-Burmazović, I.; Siegler, M. A.; Jameson, G. N. L.; De Visser, S. P.; Goldberg, D. P. Direct Observation of a Nonheme Iron(IV)-Oxo Complex That Mediates Aromatic C-F Hydroxylation. *J. Am. Chem. Soc.* **2014**, *136* (39), 13542–13545.

[9] Neu, H. M.; Yang, T.; Baglia, R. A.; Yosca, T. H.; Green, M. T.; Quesne, M. G.; de Visser, S. P.; Goldberg, D. P. Oxygen-Atom Transfer Reactivity of Axially Ligated Mn (V)–Oxo Complexes: Evidence for Enhanced Electrophilic and Nucleophilic Pathways. *J. Am. Chem. Soc.* **2014**, *136* (39), 13845–13852.

[10] Timmins, A.; Quesne, M. G.; Borowski, T.; de Visser, S. P. Group Transfer to an Aliphatic Bond: A Biomimetic Study Inspired by Nonheme Iron Halogenases. *ACS Catal.* **2018**, 8685–8698.

[11] Grimme, S.; Antony, J.; Ehrlich, S.; Krieg, H. A Consistent and Accurate Ab Initio Parametrization of Density Functional Dispersion Correction (DFT-D) for the 94 Elements H-Pu. *J. Chem. Phys.* **2010**, *132*, 154104.

[12] Frisch, M. J.; Trucks, G. W.; Schlegel, H. B.; Scuseria, G. E.; Robb, M. A.; Cheeseman, J. R.; G. Scalmani, V. B.; Mennucci, B.; Petersson, G. A.; Nakatsuji, H.; Caricato, M.; Li, X.; Hratchian, H. P.; Izmaylov, A. F.; Bloino, J.; Zheng, G.; Sonnenberg, J. L.; Hada, M.; Ehara, M.; Toyota, K.; Fukuda, R.; Hasegawa, J.; Ishida, M.; Nakajima, T.; Honda, Y.; Kitao, O.; Nakai, H.; Vreven, T.; Montgomery, J. A.; Peralta, J. E.; Ogliaro, F.; Bearpark, M.; Heyd, J. J.; Brothers, E.; Kudin, K. N.; Staroverov, V. N.; Kobayashi, R.; Normand, J.; Raghavachari, K.; Rendell, A.; Burant, J. C.; Iyengar, S. S.; Tomasi, J.; Cossi, M.; Rega, N.; Millam, J. M.; Klene, M.; Knox, J. E.; Cross, J. B.; Bakken, V.; Adamo, C.; Jaramillo, J.; Gomperts, R.; Stratmann, R. E.; Yazyev, O.; Austin, A. J.; Cammi, R.; Pomelli, C.; Ochterski, J. W.; Martin, R. L.; Morokuma, K.; Zakrzewski, V. G.; Voth, G. A.; Salvador, P.; Dannenberg, J.

J.; Dapprich, S.; Daniels, A. D.; Farkas; Foresman, J. B.; Ortiz, J. V; Cioslowski, J.; Fox, D. J. Gaussian 09, Revis. B.01, Gaussian, Inc., Wallingford CT, 200.
[13] Kang, D. S.; Erman, J. E. The Cytochrome c Peroxidase-Catalyzed Oxidation of Ferrocytochrome c by Hydrogen Peroxide. Steady State Kinetic Mechanism. *J. Biol. Chem.* **1982**, *257* (21), 12775–12779.
[14] Meharenna, Y. T.; Doukov, T.; Li, H.; Soltis, S. M.; Poulos, T. L. Crystallographic and Single-Crystal Spectral Analysis of the Peroxidase Ferryl Intermediate. *Biochemistry* **2010**, *49* (14), 2984–2986.
[15] Berglund, G. I.; Carlsson, G. H.; Smith, A. T.; Szöke, H.; Henriksen, A.; Hajdu, J. The Catalytic Pathway of Horseradish Peroxidase at High Resolution. *Nature* **2002**, *417* (6887), 463–468.

Cartesian Coordinates

^{2}C*c*P W191F CpdI

6	-12.856997000	-0.597005000	-1.739002000
7	-12.197158000	-0.799453000	-0.456322000
6	-12.432937000	-1.818843000	0.360785000
7	-13.446815000	-2.675948000	0.136907000
7	-11.612595000	-2.042908000	1.405334000
1	-13.881888000	-0.223754000	-1.618969000
1	-12.863131000	-1.524297000	-2.320164000
1	-11.717843000	0.025743000	-0.034458000
1	-14.190078000	-2.451995000	-0.508724000
1	-13.481758000	-3.571663000	0.603669000
1	-10.768265000	-1.474007000	1.494479000
1	-12.004296000	-2.402096000	2.267971000
6	-9.070002000	4.293031000	-4.655004000
6	-9.025906000	3.565963000	-3.347654000
6	-10.066468000	2.924875000	-2.712980000
6	-7.889821000	3.461969000	-2.461269000
7	-9.644232000	2.402904000	-1.498247000
6	-8.319463000	2.752445000	-1.304926000
6	-6.559435000	3.914906000	-2.531069000
6	-7.463810000	2.492887000	-0.229311000
6	-5.703656000	3.658982000	-1.462978000
6	-6.152993000	2.957871000	-0.322823000
1	-8.847958000	5.359615000	-4.521206000
1	-10.057125000	4.214012000	-5.122281000
1	-11.096283000	2.815205000	-3.025369000
1	-10.254574000	2.081050000	-0.742381000
1	-6.206032000	4.461712000	-3.401005000
1	-7.812459000	1.973996000	0.657705000
1	-4.675967000	4.007678000	-1.501296000
1	-5.465197000	2.784084000	0.500296000
6	-14.935000000	6.433999000	3.954998000
6	-14.000912000	5.292055000	3.760723000
7	-13.362354000	4.660322000	4.812859000
6	-13.588183000	4.623237000	2.637627000
6	-12.608457000	3.652763000	4.332561000
7	-12.723495000	3.604715000	3.007356000
1	-14.436967000	7.281778000	4.440091000
1	-15.789298000	6.139436000	4.573828000
1	-13.480549000	4.895341000	5.800524000
1	-13.854410000	4.801091000	1.609332000
1	-12.001937000	3.003680000	4.943575000
6	-19.304001000	5.819000000	6.472000000
6	-18.277630000	4.925542000	5.811278000
6	-17.238999000	4.346196000	6.559299000
6	-18.351521000	4.621523000	4.443020000
6	-16.317874000	3.479180000	5.966052000
6	-17.427657000	3.759695000	3.843469000

6	-16.407792000	3.181188000	4.603837000
1	-19.758466000	6.508594000	5.753451000
1	-18.859788000	6.409493000	7.280332000
1	-19.142949000	5.063207000	3.841989000
1	-15.526609000	3.042852000	6.570155000
1	-17.501741000	3.545490000	2.779945000
1	-15.683503000	2.518255000	4.137271000
6	-12.598999000	6.281000000	9.587003000
6	-12.716708000	5.456554000	8.353417000
8	-13.708643000	5.475415000	7.569656000
8	-11.816119000	4.657829000	7.944929000
1	-11.868064000	5.846695000	10.274537000
1	-13.576284000	6.365378000	10.071724000
6	-14.603557000	0.428403000	2.074305000
6	-10.320478000	0.621444000	4.347367000
6	-9.219667000	4.599480000	1.800015000
6	-13.136167000	3.908517000	-0.967123000
6	-13.560716000	0.177121000	2.955705000
6	-13.581422000	-0.844657000	3.973518000
6	-12.356665000	-0.824435000	4.582936000
6	-11.594763000	0.227840000	3.955132000
6	-9.645473000	1.725373000	3.845677000
6	-8.372523000	2.202832000	4.334596000
6	-8.088263000	3.347916000	3.648196000
6	-9.173516000	3.565250000	2.722947000
6	-10.192387000	4.750950000	0.827032000
6	-10.158387000	5.760944000	-0.204602000
6	-11.240893000	5.548856000	-1.003075000
6	-11.952586000	4.416938000	-0.455151000
6	-13.870786000	2.882009000	-0.387785000
6	-15.180137000	2.463130000	-0.827438000
6	-15.614085000	1.520541000	0.060625000
6	-14.561132000	1.340357000	1.031233000
7	-12.344958000	0.819551000	2.966077000
7	-10.110396000	2.569231000	2.861072000
7	-11.296614000	3.948879000	0.657994000
7	-13.510375000	2.178975000	0.737537000
26	-11.768289000	2.327036000	1.741199000
1	-9.846218000	0.063352000	5.148668000
1	-8.398800000	5.308263000	1.800482000
1	-13.547734000	4.385820000	-1.850672000
1	-15.513548000	-0.150324000	2.197453000
8	-10.972081000	1.297389000	0.689340000
8	-9.112850000	-0.398280000	1.248917000
1	-8.646936000	-0.208695000	2.080071000
1	-9.748442000	0.376051000	1.117367000
1	-20.113302000	5.221113000	6.910830000
1	-12.266400000	7.288402000	9.308500000
1	-15.317502000	6.771933000	2.988433000
1	-12.288433000	0.153529000	-2.291140000

1	-8.331383000	3.902507000	-5.366646000
1	-14.434434000	-1.473070000	4.197725000
1	-12.005842000	-1.430358000	5.409020000
1	-16.561704000	0.996761000	0.076213000
1	-15.700749000	2.869552000	-1.685211000
1	-11.546752000	6.097315000	-1.884291000
1	-9.387587000	6.514575000	-0.297842000
1	-7.226884000	3.995714000	3.746652000
1	-7.798621000	1.730174000	5.121628000
1	-17.156070000	4.572092000	7.619756000
6	-7.771002000	-0.935025000	-4.902995000
6	-7.702456000	-1.075059000	-3.419679000
7	-6.902743000	-2.018588000	-2.779043000
6	-8.339984000	-0.399566000	-2.413691000
6	-7.049497000	-1.922868000	-1.447597000
7	-7.920050000	-0.944067000	-1.212219000
1	-8.162743000	-1.843801000	-5.374316000
1	-6.784297000	-0.725053000	-5.331348000
1	-6.286374000	-2.675879000	-3.243805000
1	-9.020606000	0.438152000	-2.444947000
1	-6.551179000	-2.530496000	-0.707817000
1	-8.262285000	-0.667455000	-0.268947000
1	-8.431186000	-0.103479000	-5.160634000

^{2}C*c*P S-Trp W191F CpdI

6	-12.856997000	-0.596993000	-1.738994000
7	-12.256141000	-0.818997000	-0.429228000
6	-12.689197000	-1.723504000	0.446883000
7	-13.828520000	-2.413245000	0.230156000
7	-11.930875000	-2.032673000	1.512946000
1	-13.694667000	0.107964000	-1.685719000
1	-13.202905000	-1.546143000	-2.158930000
1	-11.703584000	-0.037235000	-0.012879000
1	-14.576756000	-1.994987000	-0.305541000
1	-14.079925000	-3.175355000	0.845583000
1	-11.010478000	-1.596193000	1.598636000
1	-12.387301000	-2.277594000	2.385760000
6	-9.070012000	4.293027000	-4.654989000
6	-9.251636000	3.376506000	-3.488062000
6	-10.296432000	2.518829000	-3.329847000
6	-8.337529000	3.320844000	-2.364411000
16	-10.273899000	1.649515000	-1.813329000
6	-8.768849000	2.423368000	-1.355278000
6	-7.135911000	4.028082000	-2.177409000
6	-8.029731000	2.230937000	-0.184219000
6	-6.390605000	3.826327000	-1.020105000
6	-6.836775000	2.932458000	-0.029056000
1	-9.059326000	5.342537000	-4.333929000
1	-9.874381000	4.173989000	-5.386631000
1	-11.127656000	2.377488000	-4.010379000

1	-6.792932000	4.725548000	-2.936300000
1	-8.393450000	1.582715000	0.602227000
1	-5.458724000	4.365235000	-0.877470000
1	-6.254302000	2.798629000	0.878046000
6	-14.935018000	6.434001000	3.955003000
6	-14.007953000	5.276114000	3.799900000
7	-13.438837000	4.630852000	4.883013000
6	-13.543344000	4.598961000	2.699565000
6	-12.679784000	3.607676000	4.441391000
7	-12.720595000	3.562147000	3.113190000
1	-14.444733000	7.276361000	4.457354000
1	-15.817043000	6.151423000	4.539707000
1	-13.611194000	4.856315000	5.862638000
1	-13.745089000	4.782371000	1.657081000
1	-12.124104000	2.944112000	5.084509000
6	-19.303996000	5.819006000	6.471997000
6	-18.294081000	4.918566000	5.796815000
6	-17.278801000	4.291962000	6.540302000
6	-18.359662000	4.654913000	4.419794000
6	-16.372309000	3.420274000	5.932835000
6	-17.449418000	3.788563000	3.806406000
6	-16.452107000	3.164689000	4.561217000
1	-19.762555000	6.513092000	5.760589000
1	-18.844339000	6.404746000	7.275330000
1	-19.133042000	5.132559000	3.822928000
1	-15.599926000	2.946141000	6.532860000
1	-17.515000000	3.607217000	2.736289000
1	-15.737259000	2.499813000	4.083744000
6	-12.598997000	6.281001000	9.587008000
6	-12.785045000	5.399704000	8.404389000
8	-13.823039000	5.366383000	7.681895000
8	-11.908923000	4.590292000	7.967268000
1	-11.819225000	5.885999000	10.243926000
1	-13.543611000	6.378174000	10.130583000
6	-14.732076000	0.544247000	2.066583000
6	-10.578904000	0.385386000	4.567185000
6	-9.115423000	4.364567000	2.213953000
6	-12.795368000	3.862716000	-0.891879000
6	-13.771706000	0.217288000	3.015363000
6	-13.926026000	-0.812448000	4.016156000
6	-12.732212000	-0.903705000	4.679671000
6	-11.858893000	0.094760000	4.107493000
6	-9.809289000	1.457816000	4.134539000
6	-8.550013000	1.856183000	4.721627000
6	-8.170327000	3.009882000	4.098663000
6	-9.178495000	3.305902000	3.108479000
6	-9.990488000	4.565206000	1.158843000
6	-9.827348000	5.586967000	0.149766000
6	-10.832522000	5.413845000	-0.753248000
6	-11.628847000	4.301817000	-0.285932000

6	-13.636119000	2.888463000	-0.370646000
6	-14.940874000	2.566379000	-0.897096000
6	-15.509421000	1.674563000	-0.031315000
6	-14.542441000	1.427061000	1.012089000
7	-12.512432000	0.758760000	3.098095000
7	-10.166081000	2.352307000	3.151692000
7	-11.091388000	3.797032000	0.871945000
7	-13.409550000	2.171665000	0.780521000
26	-11.741336000	2.208970000	1.911966000
1	-10.184709000	-0.221704000	5.376180000
1	-8.278656000	5.048812000	2.306260000
1	-13.103661000	4.358593000	-1.806471000
1	-15.694823000	0.047270000	2.137363000
8	-10.944398000	1.101154000	0.968154000
8	-9.223991000	-0.756637000	1.378014000
1	-8.721225000	-0.724003000	2.207985000
1	-9.790599000	0.079687000	1.353358000
1	-20.111798000	5.226945000	6.921466000
1	-12.298277000	7.277610000	9.241240000
1	-15.274366000	6.775237000	2.973564000
1	-12.094793000	-0.183490000	-2.398713000
1	-8.118016000	4.108384000	-5.168852000
1	-14.838176000	-1.368728000	4.194251000
1	-12.473189000	-1.547917000	5.510725000
1	-16.497008000	1.232076000	-0.074586000
1	-15.370074000	3.002780000	-1.790187000
1	-11.038756000	5.981598000	-1.651093000
1	-9.029965000	6.318196000	0.140979000
1	-7.291428000	3.614859000	4.281098000
1	-8.051627000	1.331244000	5.526749000
1	-17.204354000	4.484371000	7.607985000
6	-7.770980000	-0.935041000	-4.903024000
6	-8.142368000	-1.152013000	-3.472533000
7	-9.110679000	-2.078559000	-3.089427000
6	-7.724864000	-0.567384000	-2.305130000
6	-9.275809000	-2.055989000	-1.758208000
7	-8.437661000	-1.146681000	-1.269661000
1	-8.631448000	-0.584718000	-5.484294000
1	-7.391217000	-1.854419000	-5.362845000
1	-9.596588000	-2.704382000	-3.721158000
1	-7.015708000	0.226327000	-2.131543000
1	-9.959562000	-2.660336000	-1.184038000
1	-8.442326000	-0.888802000	-0.266547000
1	-6.988320000	-0.176066000	-4.970804000

^{4}C*c*P W191F CpdI

6	-12.857000000	-0.597000000	-1.739000000
7	-12.197200000	-0.799500000	-0.456300000
6	-12.432900000	-1.818800000	0.360800000
7	-13.446800000	-2.675900000	0.136900000

7	-11.612600000	-2.042900000	1.405300000
1	-13.881900000	-0.223800000	-1.619000000
1	-12.863100000	-1.524300000	-2.320200000
1	-11.717800000	0.025700000	-0.034500000
1	-14.190100000	-2.452000000	-0.508700000
1	-13.481800000	-3.571700000	0.603700000
1	-10.768300000	-1.474000000	1.494500000
1	-12.004300000	-2.402100000	2.268000000
6	-9.070000000	4.293000000	-4.655000000
6	-9.025900000	3.566000000	-3.347700000
6	-10.066500000	2.924900000	-2.713000000
6	-7.889800000	3.462000000	-2.461300000
7	-9.644200000	2.402900000	-1.498200000
6	-8.319500000	2.752400000	-1.304900000
6	-6.559400000	3.914900000	-2.531100000
6	-7.463800000	2.492900000	-0.229300000
6	-5.703700000	3.659000000	-1.463000000
6	-6.153000000	2.957900000	-0.322800000
1	-8.848000000	5.359600000	-4.521200000
1	-10.057100000	4.214000000	-5.122300000
1	-11.096300000	2.815200000	-3.025400000
1	-10.254600000	2.081000000	-0.742400000
1	-6.206000000	4.461700000	-3.401000000
1	-7.812500000	1.974000000	0.657700000
1	-4.676000000	4.007700000	-1.501300000
1	-5.465200000	2.784100000	0.500300000
6	-14.935000000	6.434000000	3.955000000
6	-14.000900000	5.292100000	3.760700000
7	-13.362400000	4.660300000	4.812900000
6	-13.588200000	4.623200000	2.637600000
6	-12.608500000	3.652800000	4.332600000
7	-12.723500000	3.604700000	3.007400000
1	-14.437000000	7.281800000	4.440100000
1	-15.789300000	6.139400000	4.573800000
1	-13.480500000	4.895300000	5.800500000
1	-13.854400000	4.801100000	1.609300000
1	-12.001900000	3.003700000	4.943600000
6	-19.304000000	5.819000000	6.472000000
6	-18.277600000	4.925500000	5.811300000
6	-17.239000000	4.346200000	6.559300000
6	-18.351500000	4.621500000	4.443000000
6	-16.317900000	3.479200000	5.966100000
6	-17.427700000	3.759700000	3.843500000
6	-16.407800000	3.181200000	4.603800000
1	-19.758500000	6.508600000	5.753500000
1	-18.859800000	6.409500000	7.280300000
1	-19.143000000	5.063200000	3.842000000
1	-15.526600000	3.042900000	6.570200000
1	-17.501700000	3.545500000	2.779900000
1	-15.683500000	2.518300000	4.137300000

6	-12.599000000	6.281000000	9.587000000
6	-12.716700000	5.456600000	8.353400000
8	-13.708600000	5.475400000	7.569700000
8	-11.816100000	4.657800000	7.944900000
1	-11.868100000	5.846700000	10.274500000
1	-13.576300000	6.365400000	10.071700000
6	-14.603600000	0.428400000	2.074300000
6	-10.320500000	0.621400000	4.347400000
6	-9.219700000	4.599500000	1.800000000
6	-13.136200000	3.908500000	-0.967100000
6	-13.560700000	0.177100000	2.955700000
6	-13.581400000	-0.844700000	3.973500000
6	-12.356700000	-0.824400000	4.582900000
6	-11.594800000	0.227800000	3.955100000
6	-9.645500000	1.725400000	3.845700000
6	-8.372500000	2.202800000	4.334600000
6	-8.088300000	3.347900000	3.648200000
6	-9.173500000	3.565200000	2.722900000
6	-10.192400000	4.750900000	0.827000000
6	-10.158400000	5.760900000	-0.204600000
6	-11.240900000	5.548900000	-1.003100000
6	-11.952600000	4.416900000	-0.455200000
6	-13.870800000	2.882000000	-0.387800000
6	-15.180100000	2.463100000	-0.827400000
6	-15.614100000	1.520500000	0.060600000
6	-14.561100000	1.340400000	1.031200000
7	-12.345000000	0.819600000	2.966100000
7	-10.110400000	2.569200000	2.861100000
7	-11.296600000	3.948900000	0.658000000
7	-13.510400000	2.179000000	0.737500000
26	-11.768300000	2.327000000	1.741200000
1	-9.846200000	0.063400000	5.148700000
1	-8.398800000	5.308300000	1.800500000
1	-13.547700000	4.385800000	-1.850700000
1	-15.513500000	-0.150300000	2.197500000
8	-10.972100000	1.297400000	0.689300000
8	-9.112900000	-0.398300000	1.248900000
1	-8.646900000	-0.208700000	2.080100000
1	-9.748400000	0.376100000	1.117400000
1	-20.113300000	5.221100000	6.910800000
1	-12.266400000	7.288400000	9.308500000
1	-15.317500000	6.771900000	2.988400000
1	-12.288400000	0.153500000	-2.291100000
1	-8.331400000	3.902500000	-5.366600000
1	-14.434400000	-1.473100000	4.197700000
1	-12.005800000	-1.430400000	5.409000000
1	-16.561700000	0.996800000	0.076200000
1	-15.700800000	2.869600000	-1.685200000
1	-11.546800000	6.097300000	-1.884300000
1	-9.387600000	6.514600000	-0.297800000

1	-7.226900000	3.995700000	3.746700000
1	-7.798600000	1.730200000	5.121600000
1	-17.156100000	4.572100000	7.619800000
6	-7.771000000	-0.935000000	-4.903000000
6	-7.702500000	-1.075100000	-3.419700000
7	-6.902700000	-2.018600000	-2.779000000
6	-8.340000000	-0.399600000	-2.413700000
6	-7.049500000	-1.922900000	-1.447600000
7	-7.920100000	-0.944100000	-1.212200000
1	-8.162700000	-1.843800000	-5.374300000
1	-6.784300000	-0.725100000	-5.331300000
1	-6.286400000	-2.675900000	-3.243800000
1	-9.020600000	0.438200000	-2.444900000
1	-6.551200000	-2.530500000	-0.707800000
1	-8.262300000	-0.667500000	-0.268900000
1	-8.431200000	-0.103500000	-5.160600000

^{4}C*c*P S-Trp W191F CpdI

6	-12.856997000	-0.596993000	-1.738994000
7	-12.256141000	-0.818997000	-0.429228000
6	-12.689197000	-1.723504000	0.446883000
7	-13.828520000	-2.413245000	0.230156000
7	-11.930875000	-2.032673000	1.512946000
1	-13.694667000	0.107964000	-1.685719000
1	-13.202905000	-1.546143000	-2.158930000
1	-11.703584000	-0.037235000	-0.012879000
1	-14.576756000	-1.994987000	-0.305541000
1	-14.079925000	-3.175355000	0.845583000
1	-11.010478000	-1.596193000	1.598636000
1	-12.387301000	-2.277594000	2.385760000
6	-9.070012000	4.293027000	-4.654989000
6	-9.251636000	3.376506000	-3.488062000
6	-10.296432000	2.518829000	-3.329847000
6	-8.337529000	3.320844000	-2.364411000
16	-10.273899000	1.649515000	-1.813329000
6	-8.768849000	2.423368000	-1.355278000
6	-7.135911000	4.028082000	-2.177409000
6	-8.029731000	2.230937000	-0.184219000
6	-6.390605000	3.826327000	-1.020105000
6	-6.836775000	2.932458000	-0.029056000
1	-9.059326000	5.342537000	-4.333929000
1	-9.874381000	4.173989000	-5.386631000
1	-11.127656000	2.377488000	-4.010379000
1	-6.792932000	4.725548000	-2.936300000
1	-8.393450000	1.582715000	0.602227000
1	-5.458724000	4.365235000	-0.877470000
1	-6.254302000	2.798629000	0.878046000
6	-14.935018000	6.434001000	3.955003000
6	-14.007953000	5.276114000	3.799900000
7	-13.438837000	4.630852000	4.883013000

6	-13.543344000	4.598961000	2.699565000
6	-12.679784000	3.607676000	4.441391000
7	-12.720595000	3.562147000	3.113190000
1	-14.444733000	7.276361000	4.457354000
1	-15.817043000	6.151423000	4.539707000
1	-13.611194000	4.856315000	5.862638000
1	-13.745089000	4.782371000	1.657081000
1	-12.124104000	2.944112000	5.084509000
6	-19.303996000	5.819006000	6.471997000
6	-18.294081000	4.918566000	5.796815000
6	-17.278801000	4.291962000	6.540302000
6	-18.359662000	4.654913000	4.419794000
6	-16.372309000	3.420274000	5.932835000
6	-17.449418000	3.788563000	3.806406000
6	-16.452107000	3.164689000	4.561217000
1	-19.762555000	6.513092000	5.760589000
1	-18.844339000	6.404746000	7.275330000
1	-19.133042000	5.132559000	3.822928000
1	-15.599926000	2.946141000	6.532860000
1	-17.515000000	3.607217000	2.736289000
1	-15.737259000	2.499813000	4.083744000
6	-12.598997000	6.281001000	9.587008000
6	-12.785045000	5.399704000	8.404389000
8	-13.823039000	5.366383000	7.681895000
8	-11.908923000	4.590292000	7.967268000
1	-11.819225000	5.885999000	10.243926000
1	-13.543611000	6.378174000	10.130583000
6	-14.732076000	0.544247000	2.066583000
6	-10.578904000	0.385386000	4.567185000
6	-9.115423000	4.364567000	2.213953000
6	-12.795368000	3.862716000	-0.891879000
6	-13.771706000	0.217288000	3.015363000
6	-13.926026000	-0.812448000	4.016156000
6	-12.732212000	-0.903705000	4.679671000
6	-11.858893000	0.094760000	4.107493000
6	-9.809289000	1.457816000	4.134539000
6	-8.550013000	1.856183000	4.721627000
6	-8.170327000	3.009882000	4.098663000
6	-9.178495000	3.305902000	3.108479000
6	-9.990488000	4.565206000	1.158843000
6	-9.827348000	5.586967000	0.149766000
6	-10.832522000	5.413845000	-0.753248000
6	-11.628847000	4.301817000	-0.285932000
6	-13.636119000	2.888463000	-0.370646000
6	-14.940874000	2.566379000	-0.897096000
6	-15.509421000	1.674563000	-0.031315000
6	-14.542441000	1.427061000	1.012089000
7	-12.512432000	0.758760000	3.098095000
7	-10.166081000	2.352307000	3.151692000
7	-11.091388000	3.797032000	0.871945000

7	-13.409550000	2.171665000	0.780521000
26	-11.741336000	2.208970000	1.911966000
1	-10.184709000	-0.221704000	5.376180000
1	-8.278656000	5.048812000	2.306260000
1	-13.103661000	4.358593000	-1.806471000
1	-15.694823000	0.047270000	2.137363000
8	-10.944398000	1.101154000	0.968154000
8	-9.223991000	-0.756637000	1.378014000
1	-8.721225000	-0.724003000	2.207985000
1	-9.790599000	0.079687000	1.353358000
1	-20.111798000	5.226945000	6.921466000
1	-12.298277000	7.277610000	9.241240000
1	-15.274366000	6.775237000	2.973564000
1	-12.094793000	-0.183490000	-2.398713000
1	-8.118016000	4.108384000	-5.168852000
1	-14.838176000	-1.368728000	4.194251000
1	-12.473189000	-1.547917000	5.510725000
1	-16.497008000	1.232076000	-0.074586000
1	-15.370074000	3.002780000	-1.790187000
1	-11.038756000	5.981598000	-1.651093000
1	-9.029965000	6.318196000	0.140979000
1	-7.291428000	3.614859000	4.281098000
1	-8.051627000	1.331244000	5.526749000
1	-17.204354000	4.484371000	7.607985000
6	-7.770980000	-0.935041000	-4.903024000
6	-8.142368000	-1.152013000	-3.472533000
7	-9.110679000	-2.078559000	-3.089427000
6	-7.724864000	-0.567384000	-2.305130000
6	-9.275809000	-2.055989000	-1.758208000
7	-8.437661000	-1.146681000	-1.269661000
1	-8.631448000	-0.584718000	-5.484294000
1	-7.391217000	-1.854419000	-5.362845000
1	-9.596588000	-2.704382000	-3.721158000
1	-7.015708000	0.226327000	-2.131543000
1	-9.959562000	-2.660336000	-1.184038000
1	-8.442326000	-0.888802000	-0.266547000
1	-6.988320000	-0.176066000	-4.970804000

^{3}C*c*P W191F CpdII

6	-12.856963000	-0.596935000	-1.739033000
7	-12.158197000	-0.688812000	-0.461703000
6	-12.393242000	-1.633589000	0.435727000
7	-13.416368000	-2.509118000	0.276895000
7	-11.545067000	-1.807869000	1.471125000
1	-13.876323000	-0.207305000	-1.621759000
1	-12.890393000	-1.575122000	-2.229428000
1	-11.689823000	0.194426000	-0.073812000
1	-14.252433000	-2.201096000	-0.201716000
1	-13.539545000	-3.247597000	0.956626000

1	-10.723688000	-1.197368000	1.521998000
1	-11.934646000	-2.064920000	2.372854000
6	-9.070004000	4.292955000	-4.654956000
6	-9.009613000	3.631356000	-3.311050000
6	-10.035807000	2.999443000	-2.642384000
6	-7.877440000	3.604247000	-2.415801000
7	-9.615489000	2.572799000	-1.396492000
6	-8.303479000	2.952013000	-1.219199000
6	-6.549615000	4.065725000	-2.505524000
6	-7.441188000	2.755222000	-0.131860000
6	-5.691958000	3.869449000	-1.427509000
6	-6.135231000	3.221922000	-0.251546000
1	-8.900396000	5.374758000	-4.574012000
1	-10.048385000	4.145010000	-5.124985000
1	-11.061257000	2.845856000	-2.951098000
1	-10.219551000	2.193555000	-0.651393000
1	-6.200620000	4.571688000	-3.402099000
1	-7.786083000	2.279796000	0.780022000
1	-4.666518000	4.223223000	-1.484091000
1	-5.445905000	3.093703000	0.578533000
6	-14.935044000	6.433965000	3.955004000
6	-13.953088000	5.332099000	3.776938000
7	-13.141531000	4.880251000	4.813239000
6	-13.699474000	4.570803000	2.657032000
6	-12.441863000	3.878517000	4.308899000
7	-12.738552000	3.640631000	2.988056000
1	-14.448465000	7.339212000	4.339302000
1	-15.712675000	6.149896000	4.673465000
1	-12.982398000	5.611498000	6.401829000
1	-14.136808000	4.623704000	1.673148000
1	-11.713313000	3.304221000	4.861296000
6	-19.303983000	5.818997000	6.471994000
6	-18.177459000	4.930553000	5.986279000
6	-17.062411000	4.673202000	6.802057000
6	-18.215626000	4.335764000	4.717340000
6	-16.021004000	3.848013000	6.371063000
6	-17.176288000	3.507174000	4.278250000
6	-16.078052000	3.259797000	5.102993000
1	-20.012286000	6.040850000	5.666966000
1	-18.921874000	6.771121000	6.859109000
1	-19.064135000	4.528062000	4.063891000
1	-15.155062000	3.689272000	7.007934000
1	-17.217462000	3.071596000	3.282142000
1	-15.255389000	2.642925000	4.752069000
6	-12.598979000	6.281018000	9.586985000
6	-12.847617000	5.446583000	8.342289000
8	-12.837148000	6.182231000	7.226732000
8	-13.033137000	4.238002000	8.355600000
1	-12.636754000	5.644801000	10.472359000
1	-13.351558000	7.073420000	9.662213000

6	-14.430559000	0.322025000	2.501519000
6	-9.965113000	1.016548000	4.264278000
6	-9.577661000	4.968388000	1.485747000
6	-13.547315000	3.622273000	-0.938760000
6	-13.286183000	0.221785000	3.281546000
6	-13.107122000	-0.730344000	4.352721000
6	-11.824263000	-0.581796000	4.804934000
6	-11.229601000	0.483634000	4.032416000
6	-9.462378000	2.156605000	3.650116000
6	-8.229050000	2.816144000	4.022178000
6	-8.153439000	3.959121000	3.282101000
6	-9.322372000	3.989890000	2.434186000
6	-10.627003000	4.941993000	0.580643000
6	-10.799288000	5.890439000	-0.496562000
6	-11.886890000	5.485475000	-1.208841000
6	-12.399193000	4.300222000	-0.555857000
6	-14.130839000	2.583724000	-0.221172000
6	-15.428374000	2.017449000	-0.506425000
6	-15.703847000	1.131915000	0.497967000
6	-14.562796000	1.133659000	1.383049000
7	-12.131396000	0.943890000	3.106078000
7	-10.097718000	2.882395000	2.671571000
7	-11.613801000	3.991614000	0.524725000
7	-13.620983000	2.019529000	0.921083000
26	-11.838684000	2.429260000	1.764379000
1	-9.365193000	0.561587000	5.046400000
1	-8.866831000	5.783669000	1.404835000
1	-14.075863000	3.985583000	-1.814562000
1	-15.275741000	-0.304768000	2.769162000
8	-11.023545000	1.385520000	0.651625000
8	-9.119658000	-0.121654000	1.245028000
1	-8.652838000	0.126946000	2.059189000
1	-9.841357000	0.609728000	1.098618000
1	-19.862746000	5.339305000	7.285877000
1	-11.619960000	6.767898000	9.517919000
1	-15.420800000	6.677780000	3.004862000
1	-12.301979000	0.098618000	-2.371705000
1	-8.307920000	3.902505000	-5.342506000
1	-13.877846000	-1.395236000	4.722800000
1	-11.334205000	-1.101352000	5.618638000
1	-16.600221000	0.543134000	0.649145000
1	-16.053912000	2.301026000	-1.343489000
1	-12.322306000	5.938448000	-2.090162000
1	-10.150966000	6.737793000	-0.676805000
1	-7.383113000	4.719076000	3.291394000
1	-7.542794000	2.456671000	4.778452000
1	-17.005100000	5.133189000	7.786562000
6	-7.771026000	-0.934999000	-4.902995000
6	-8.043967000	-0.984174000	-3.438938000
7	-8.465617000	-2.141183000	-2.785487000

6	-7.969622000	-0.022219000	-2.467021000
6	-8.639304000	-1.889809000	-1.476551000
7	-8.339213000	-0.611595000	-1.273149000
1	-8.658360000	-1.206455000	-5.486874000
1	-6.954949000	-1.610915000	-5.184250000
1	-8.603885000	-3.043292000	-3.224263000
1	-7.697447000	1.020603000	-2.528416000
1	-8.965778000	-2.589146000	-0.722649000
1	-8.438464000	-0.181899000	-0.325996000
1	-7.480848000	0.079808000	-5.185171000

^{3}C*c*P S-Trp W191F CpdII

6	-12.856994000	-0.596998000	-1.738999000
7	-12.298662000	-0.751958000	-0.400626000
6	-12.809019000	-1.563307000	0.522962000
7	-14.002769000	-2.177602000	0.333214000
7	-12.077772000	-1.860607000	1.610181000
1	-13.648314000	0.161050000	-1.763020000
1	-13.259721000	-1.552278000	-2.090139000
1	-11.752326000	0.060674000	0.021939000
1	-14.729683000	-1.651262000	-0.137166000
1	-14.328337000	-2.818024000	1.045975000
1	-11.137420000	-1.463362000	1.671535000
1	-12.547430000	-2.004083000	2.498654000
6	-9.070007000	4.293018000	-4.654990000
6	-9.280207000	3.388125000	-3.486301000
6	-10.359621000	2.576634000	-3.318206000
6	-8.379373000	3.321520000	-2.355258000
16	-10.394980000	1.754150000	-1.778886000
6	-8.860474000	2.469994000	-1.328062000
6	-7.149961000	3.981280000	-2.172433000
6	-8.144112000	2.279275000	-0.142487000
6	-6.425590000	3.776120000	-1.002327000
6	-6.922600000	2.930348000	0.007085000
1	-9.054800000	5.344251000	-4.338759000
1	-9.866188000	4.178702000	-5.396835000
1	-11.196550000	2.466992000	-3.997957000
1	-6.770700000	4.646121000	-2.943583000
1	-8.554229000	1.681334000	0.659736000
1	-5.474551000	4.281152000	-0.860347000
1	-6.364664000	2.804161000	0.930356000
6	-14.935011000	6.434010000	3.955005000
6	-13.947816000	5.321053000	3.818602000
7	-13.172987000	4.887667000	4.885638000
6	-13.670560000	4.523650000	2.730688000
6	-12.471313000	3.856510000	4.432867000
7	-12.730602000	3.584478000	3.119637000
1	-14.458239000	7.341401000	4.345530000
1	-15.737172000	6.157700000	4.648754000
1	-13.070366000	5.625873000	6.406029000

1	-14.077026000	4.549906000	1.732255000
1	-11.770553000	3.291500000	5.029925000
6	-19.304000000	5.819000000	6.472000000
6	-18.181206000	4.916972000	6.002902000
6	-17.066499000	4.673349000	6.823367000
6	-18.216266000	4.306000000	4.741968000
6	-16.020951000	3.849577000	6.401373000
6	-17.171446000	3.478773000	4.312512000
6	-16.072012000	3.247889000	5.139645000
1	-20.061184000	5.951036000	5.692106000
1	-18.927107000	6.811519000	6.747847000
1	-19.065010000	4.487050000	4.085543000
1	-15.153148000	3.701284000	7.038000000
1	-17.207291000	3.034608000	3.319941000
1	-15.242113000	2.638297000	4.794520000
6	-12.598999000	6.281001000	9.587003000
6	-12.769040000	5.447443000	8.328528000
8	-12.989594000	6.198012000	7.249684000
8	-12.705458000	4.225025000	8.307307000
1	-12.467908000	5.627650000	10.451004000
1	-13.473379000	6.924639000	9.731757000
6	-14.566250000	0.378784000	2.813186000
6	-10.035153000	0.829000000	4.474587000
6	-9.517487000	4.726611000	1.643520000
6	-13.471253000	3.400367000	-0.815411000
6	-13.424315000	0.240220000	3.592154000
6	-13.286377000	-0.694775000	4.686514000
6	-11.982670000	-0.634456000	5.096370000
6	-11.334496000	0.368335000	4.282082000
6	-9.492765000	1.944284000	3.847020000
6	-8.236581000	2.565459000	4.210028000
6	-8.132226000	3.706705000	3.469703000
6	-9.302067000	3.768097000	2.623415000
6	-10.538632000	4.685998000	0.707195000
6	-10.653290000	5.588769000	-0.417825000
6	-11.734677000	5.183596000	-1.140266000
6	-12.301589000	4.047811000	-0.444544000
6	-14.110884000	2.427392000	-0.055306000
6	-15.440226000	1.924212000	-0.314314000
6	-15.776514000	1.128325000	0.746633000
6	-14.640767000	1.116961000	1.638925000
7	-12.226638000	0.870259000	3.369977000
7	-10.110052000	2.690254000	2.874738000
7	-11.548237000	3.760886000	0.662108000
7	-13.639820000	1.900876000	1.120816000
26	-11.843545000	2.263461000	1.960601000
1	-9.444045000	0.352558000	5.250679000
1	-8.787176000	5.523720000	1.553654000
1	-13.971217000	3.743897000	-1.715747000
1	-15.453607000	-0.166105000	3.121162000

8	-11.054537000	1.125119000	0.965850000
8	-9.328411000	-0.657696000	1.324817000
1	-8.822617000	-0.572556000	2.149051000
1	-9.951299000	0.168537000	1.285149000
1	-19.802717000	5.404602000	7.357252000
1	-11.727265000	6.936367000	9.480705000
1	-15.388096000	6.670318000	2.986509000
1	-12.056363000	-0.280604000	-2.408686000
1	-8.112912000	4.097564000	-5.156217000
1	-14.092886000	-1.290783000	5.095628000
1	-11.509126000	-1.171304000	5.908512000
1	-16.713682000	0.618439000	0.934686000
1	-16.047684000	2.196523000	-1.168556000
1	-12.137125000	5.610947000	-2.049578000
1	-9.978665000	6.411795000	-0.614520000
1	-7.343037000	4.447374000	3.481493000
1	-7.558890000	2.188815000	4.965690000
1	-17.011691000	5.146760000	7.801606000
6	-7.770988000	-0.935031000	-4.903019000
6	-8.209455000	-1.078630000	-3.478650000
7	-9.249144000	-1.930929000	-3.108072000
6	-7.789505000	-0.499976000	-2.308013000
6	-9.448010000	-1.871170000	-1.781528000
7	-8.565109000	-1.014201000	-1.283236000
1	-8.587519000	-0.561147000	-5.531113000
1	-7.424213000	-1.890599000	-5.312365000
1	-9.769861000	-2.523084000	-3.743281000
1	-7.036987000	0.250978000	-2.125973000
1	-10.199742000	-2.398534000	-1.216694000
1	-8.587212000	-0.750666000	-0.272966000
1	-6.946650000	-0.220836000	-4.964676000

^{5}C*c*P W191F CpdII

6	-12.856982000	-0.597004000	-1.738977000
7	-12.052583000	-0.606807000	-0.526548000
6	-12.193049000	-1.494838000	0.448381000
7	-13.211289000	-2.390327000	0.426190000
7	-11.264688000	-1.570990000	1.416898000
1	-13.875793000	-0.234984000	-1.555014000
1	-12.894544000	-1.599595000	-2.177718000
1	-11.567777000	0.272705000	-0.220313000
1	-14.097909000	-2.098625000	0.035007000
1	-13.263223000	-3.081130000	1.162773000
1	-10.446341000	-0.950073000	1.372240000
1	-11.565460000	-1.811213000	2.354487000
6	-9.069998000	4.292997000	-4.654984000
6	-8.941350000	3.706380000	-3.280029000
6	-9.890999000	2.986247000	-2.584706000
6	-7.803257000	3.804829000	-2.400903000

7	-9.406422000	2.596015000	-1.351382000
6	-8.141332000	3.107845000	-1.197785000
6	-6.529783000	4.395596000	-2.509671000
6	-7.242508000	2.973949000	-0.132117000
6	-5.634124000	4.275511000	-1.448892000
6	-5.984941000	3.566536000	-0.272194000
1	-8.958352000	5.384811000	-4.637949000
1	-10.050121000	4.068099000	-5.088005000
1	-10.906451000	2.743254000	-2.868479000
1	-9.975762000	2.191480000	-0.582269000
1	-6.249289000	4.942831000	-3.406077000
1	-7.521684000	2.437495000	0.768457000
1	-4.655338000	4.742653000	-1.513693000
1	-5.275660000	3.507723000	0.548590000
6	-14.934999000	6.434010000	3.955120000
6	-13.904243000	5.376183000	3.752972000
7	-13.043124000	4.969687000	4.765941000
6	-13.642443000	4.627795000	2.629200000
6	-12.301466000	4.002778000	4.242305000
7	-12.619651000	3.747982000	2.937698000
1	-14.478545000	7.371774000	4.296022000
1	-15.667866000	6.129865000	4.710008000
1	-12.890284000	5.650333000	6.371961000
1	-14.110160000	4.649114000	1.658219000
1	-11.529153000	3.472468000	4.777996000
6	-19.304007000	5.819011000	6.471976000
6	-18.173985000	4.938700000	5.979143000
6	-17.054525000	4.683368000	6.788504000
6	-18.216206000	4.346608000	4.708800000
6	-16.010521000	3.864656000	6.349183000
6	-17.175449000	3.524473000	4.261725000
6	-16.070812000	3.281217000	5.079642000
1	-19.955983000	6.129382000	5.648415000
1	-18.921404000	6.722396000	6.961080000
1	-19.068404000	4.538083000	4.059636000
1	-15.139335000	3.710939000	6.979687000
1	-17.218737000	3.092088000	3.264361000
1	-15.243894000	2.674987000	4.720367000
6	-12.599013000	6.280989000	9.586894000
6	-12.854284000	5.465419000	8.327598000
8	-12.732716000	6.195571000	7.216761000
8	-13.136248000	4.275261000	8.334773000
1	-12.710586000	5.645073000	10.466378000
1	-13.304687000	7.117354000	9.637703000
6	-13.877523000	0.185533000	2.976429000
6	-9.406421000	1.724997000	4.132870000
6	-9.856605000	5.290103000	0.855822000
6	-13.941771000	3.242809000	-0.811333000
6	-12.646056000	0.307868000	3.627540000
6	-12.207890000	-0.482575000	4.763503000

6	-10.930029000	-0.088648000	5.057703000
6	-10.585501000	0.963158000	4.118476000
6	-9.118024000	2.854482000	3.352445000
6	-7.978688000	3.740522000	3.527651000
6	-8.133215000	4.766337000	2.639681000
6	-9.354813000	4.507987000	1.897990000
6	-10.994915000	5.042798000	0.080287000
6	-11.431950000	5.834932000	-1.054482000
6	-12.557406000	5.239298000	-1.549009000
6	-12.827890000	4.083741000	-0.711654000
6	-14.307582000	2.216396000	0.069687000
6	-15.556445000	1.478582000	0.040584000
6	-15.555555000	0.648056000	1.129774000
6	-14.298539000	0.859655000	1.823303000
7	-11.633569000	1.154464000	3.264792000
7	-9.911485000	3.347006000	2.357581000
7	-11.860154000	4.000429000	0.249827000
7	-13.577022000	1.802749000	1.148553000
26	-11.699544000	2.516663000	1.694729000
1	-8.683682000	1.475178000	4.905055000
1	-9.281145000	6.174124000	0.597481000
1	-14.639329000	3.459315000	-1.615872000
1	-14.583831000	-0.518921000	3.407784000
8	-10.910339000	1.494632000	0.617929000
8	-8.671985000	-0.233909000	1.591198000
1	-8.890003000	0.347596000	2.345697000
1	-19.925812000	5.290671000	7.206647000
1	-11.590390000	6.706816000	9.556848000
1	-15.471361000	6.631859000	3.021346000
1	-12.382710000	0.081978000	-2.450995000
1	-8.304651000	3.903295000	-5.339554000
1	-12.814854000	-1.211858000	5.286033000
1	-10.295982000	-0.436959000	5.863445000
1	-16.342170000	-0.020665000	1.458069000
1	-16.342277000	1.613612000	-0.692583000
1	-13.166807000	5.555818000	-2.386349000
1	-10.935454000	6.727071000	-1.414358000
1	-7.488358000	5.622694000	2.489428000
1	-7.191391000	3.602684000	4.258267000
1	-16.992858000	5.142182000	7.773349000
6	-7.771001000	-0.935003000	-4.903027000
6	-6.906851000	-0.162415000	-3.967414000
7	-5.523115000	-0.066767000	-4.105965000
6	-7.215033000	0.588313000	-2.866044000
6	-5.014322000	0.711442000	-3.136117000
7	-6.031539000	1.107460000	-2.378917000
1	-7.516138000	-2.001261000	-4.902969000
1	-7.683949000	-0.561486000	-5.929947000
1	-4.974305000	-0.512073000	-4.831883000
1	-8.163479000	0.822312000	-2.406657000

1	-3.976667000	0.976219000	-3.004080000
1	-5.967760000	1.759236000	-1.584743000
1	-8.815397000	-0.836877000	-4.597917000
1	-8.059191000	-0.893505000	1.953018000

[5]C*c*P S-Trp W191F CpdII

6	-12.856993000	-0.596990000	-1.739002000
7	-12.269737000	-0.778337000	-0.415167000
6	-12.723852000	-1.649031000	0.483176000
7	-13.869520000	-2.340504000	0.266089000
7	-11.980049000	-1.937375000	1.564798000
1	-13.686864000	0.119909000	-1.716289000
1	-13.213254000	-1.556689000	-2.125511000
1	-11.744659000	0.033249000	0.011651000
1	-14.626261000	-1.863378000	-0.207698000
1	-14.155204000	-3.017119000	0.962290000
1	-11.069137000	-1.480947000	1.649346000
1	-12.454196000	-2.119531000	2.445959000
6	-9.070005000	4.293014000	-4.654984000
6	-9.295507000	3.406766000	-3.474408000
6	-10.395658000	2.627696000	-3.287031000
6	-8.382824000	3.321884000	-2.353851000
16	-10.435108000	1.815988000	-1.741931000
6	-8.878387000	2.496932000	-1.312312000
6	-7.130183000	3.942519000	-2.193826000
6	-8.157705000	2.303874000	-0.129803000
6	-6.399076000	3.728558000	-1.029520000
6	-6.913836000	2.916105000	-0.001797000
1	-9.011515000	5.345798000	-4.349471000
1	-9.880593000	4.200674000	-5.384143000
1	-11.242901000	2.535221000	-3.956451000
1	-6.739302000	4.586325000	-2.976883000
1	-8.580709000	1.735539000	0.686750000
1	-5.430346000	4.203619000	-0.904942000
1	-6.350533000	2.786101000	0.917916000
6	-14.935031000	6.434004000	3.954989000
6	-13.975822000	5.296531000	3.827034000
7	-13.279280000	4.794745000	4.919428000
6	-13.653832000	4.541119000	2.723185000
6	-12.573971000	3.768132000	4.469179000
7	-12.765949000	3.558530000	3.128964000
1	-14.452511000	7.308614000	4.408101000
1	-15.781700000	6.156728000	4.593463000
1	-13.298428000	5.473915000	6.488985000
1	-13.998463000	4.621038000	1.705943000
1	-11.916335000	3.171096000	5.082160000
6	-19.303994000	5.819000000	6.471997000
6	-18.258551000	4.901067000	5.874836000
6	-17.144711000	4.499081000	6.630687000
6	-18.365965000	4.430474000	4.558942000

6	-16.173469000	3.653836000	6.092422000
6	-17.392390000	3.585091000	4.013223000
6	-16.292783000	3.193114000	4.777564000
1	-20.059288000	6.098917000	5.730340000
1	-18.849964000	6.740478000	6.856325000
1	-19.215724000	4.735400000	3.951742000
1	-15.305539000	3.382287000	6.685284000
1	-17.485341000	3.249726000	2.982582000
1	-15.517733000	2.565921000	4.346431000
6	-12.598987000	6.281005000	9.587022000
6	-12.565940000	5.481658000	8.295399000
8	-13.369169000	5.995476000	7.361751000
8	-11.879331000	4.483241000	8.126615000
1	-11.979018000	5.793918000	10.341293000
1	-13.629255000	6.371215000	9.947468000
6	-14.786360000	0.450646000	2.149810000
6	-10.613401000	0.514990000	4.665337000
6	-9.236900000	4.442789000	2.147935000
6	-13.062868000	3.914055000	-0.803617000
6	-13.816822000	0.163493000	3.117791000
6	-13.924405000	-0.857807000	4.142203000
6	-12.730376000	-0.874967000	4.817577000
6	-11.893495000	0.150706000	4.225278000
6	-9.837523000	1.583372000	4.202426000
6	-8.580772000	2.037163000	4.770292000
6	-8.220109000	3.167007000	4.089745000
6	-9.240513000	3.406465000	3.086951000
6	-10.147606000	4.652979000	1.110272000
6	-10.052753000	5.696554000	0.105175000
6	-11.110475000	5.532418000	-0.742116000
6	-11.870485000	4.394355000	-0.256770000
6	-13.867914000	2.887332000	-0.295957000
6	-15.176608000	2.515448000	-0.796322000
6	-15.679891000	1.570644000	0.058544000
6	-14.675832000	1.345357000	1.080154000
7	-12.581957000	0.743785000	3.202696000
7	-10.190706000	2.428349000	3.185972000
7	-11.248444000	3.887168000	0.847435000
7	-13.595147000	2.144170000	0.821093000
26	-11.850117000	2.254750000	1.962509000
1	-10.213101000	-0.046333000	5.504833000
1	-8.409210000	5.142954000	2.208710000
1	-13.433348000	4.428950000	-1.685479000
1	-15.727150000	-0.086915000	2.235742000
8	-11.030985000	1.133371000	0.981252000
8	-9.270864000	-0.632345000	1.364512000
1	-8.764088000	-0.559828000	2.189429000
1	-9.893770000	0.181267000	1.339355000
1	-19.821007000	5.337860000	7.311857000
1	-12.228888000	7.295554000	9.401383000

1	-15.326718000	6.722793000	2.974198000
1	-12.083788000	-0.211609000	-2.404307000
1	-8.128025000	4.057837000	-5.167783000
1	-14.809962000	-1.449418000	4.341572000
1	-12.455965000	-1.487114000	5.667831000
1	-16.651993000	1.093328000	0.024925000
1	-15.657953000	2.952774000	-1.662397000
1	-11.370453000	6.125925000	-1.609400000
1	-9.272990000	6.446177000	0.064408000
1	-7.354187000	3.795461000	4.255278000
1	-8.073027000	1.572545000	5.606124000
1	-17.029153000	4.866048000	7.648203000
6	-7.770989000	-0.935033000	-4.903022000
6	-8.188457000	-1.058409000	-3.470277000
7	-9.179217000	-1.951856000	-3.063123000
6	-7.785040000	-0.426319000	-2.321691000
6	-9.365087000	-1.866362000	-1.736200000
7	-8.521992000	-0.950914000	-1.273686000
1	-8.613011000	-0.630842000	-5.535022000
1	-7.371355000	-1.881334000	-5.284929000
1	-9.676097000	-2.587432000	-3.675121000
1	-7.069058000	0.365785000	-2.168909000
1	-10.077670000	-2.420545000	-1.146932000
1	-8.532923000	-0.670595000	-0.270953000
1	-6.990929000	-0.175828000	-4.995116000

^{2}C*c*P W191F Reduced CpdII

6	-12.856970000	-0.596935000	-1.739039000
7	-12.179769000	-0.820402000	-0.473691000
6	-12.674224000	-1.641889000	0.399412000
7	-13.902326000	-2.263054000	0.265746000
7	-11.917948000	-2.034163000	1.481034000
1	-13.723321000	0.074102000	-1.633902000
1	-13.204257000	-1.534978000	-2.198810000
1	-11.444409000	0.410907000	0.196894000
1	-14.634672000	-1.701768000	-0.150401000
1	-14.215577000	-2.824294000	1.047378000
1	-10.997113000	-1.600521000	1.539746000
1	-12.391844000	-2.066249000	2.378432000
6	-9.070003000	4.292939000	-4.654964000
6	-8.973328000	3.539208000	-3.365628000
6	-9.977009000	2.838415000	-2.733254000
6	-7.824451000	3.459268000	-2.499027000
7	-9.524129000	2.321567000	-1.538154000
6	-8.215063000	2.696272000	-1.358142000
6	-6.511374000	3.961379000	-2.563878000
6	-7.336962000	2.431935000	-0.301021000
6	-5.634374000	3.699775000	-1.514274000
6	-6.045076000	2.942483000	-0.392921000
1	-8.886996000	5.366713000	-4.508234000

1	-10.065328000	4.185136000	-5.101396000
1	-11.003860000	2.685299000	-3.037608000
1	-10.096788000	1.886043000	-0.813482000
1	-6.185666000	4.547070000	-3.420469000
1	-7.658318000	1.859575000	0.563691000
1	-4.617962000	4.082752000	-1.553147000
1	-5.339760000	2.757843000	0.413387000
6	-14.935037000	6.433957000	3.954997000
6	-13.924718000	5.346964000	3.799720000
7	-13.143229000	4.892811000	4.857245000
6	-13.629761000	4.598687000	2.686798000
6	-12.416823000	3.894779000	4.366999000
7	-12.669945000	3.666865000	3.046354000
1	-14.478983000	7.350696000	4.350485000
1	-15.729804000	6.134898000	4.647756000
1	-12.981436000	5.581073000	6.386090000
1	-14.030182000	4.651915000	1.687566000
1	-11.701015000	3.322226000	4.937547000
6	-19.303984000	5.818997000	6.471993000
6	-18.195118000	4.916347000	5.970871000
6	-17.074967000	4.642899000	6.773787000
6	-18.247423000	4.338651000	4.695180000
6	-16.038862000	3.822705000	6.321459000
6	-17.212884000	3.514870000	4.234489000
6	-16.107353000	3.254532000	5.044730000
1	-20.086013000	5.946062000	5.715591000
1	-18.920552000	6.814440000	6.729585000
1	-19.100562000	4.543481000	4.051062000
1	-15.166380000	3.659723000	6.947771000
1	-17.260772000	3.096117000	3.231729000
1	-15.287135000	2.646393000	4.674674000
6	-12.598977000	6.281020000	9.586994000
6	-12.934548000	5.451169000	8.356013000
8	-12.793966000	6.142067000	7.227783000
8	-13.287071000	4.280342000	8.411121000
1	-12.768288000	5.690718000	10.489081000
1	-13.215351000	7.186424000	9.608588000
6	-14.691884000	0.579669000	2.238422000
6	-10.384883000	0.631320000	4.464597000
6	-9.131039000	4.456386000	1.773170000
6	-13.098295000	3.870113000	-0.935611000
6	-13.659354000	0.320170000	3.131126000
6	-13.725411000	-0.663342000	4.186789000
6	-12.493562000	-0.688971000	4.780960000
6	-11.685897000	0.298415000	4.105612000
6	-9.656179000	1.676965000	3.914882000
6	-8.344619000	2.092260000	4.360132000
6	-8.012668000	3.195326000	3.628031000
6	-9.112433000	3.444362000	2.723769000
6	-10.118192000	4.642252000	0.817543000

6	-10.041990000	5.621204000	-0.244946000
6	-11.142102000	5.440053000	-1.026059000
6	-11.899592000	4.358128000	-0.435674000
6	-13.865857000	2.880868000	-0.331055000
6	-15.189446000	2.492973000	-0.761309000
6	-15.658319000	1.603709000	0.163760000
6	-14.611075000	1.430105000	1.144503000
7	-12.410933000	0.891888000	3.101032000
7	-10.097203000	2.508382000	2.913121000
7	-11.258246000	3.891306000	0.683179000
7	-13.527572000	2.207363000	0.816661000
26	-11.843838000	2.415828000	1.909887000
1	-9.928337000	0.069697000	5.273864000
1	-8.271285000	5.116931000	1.732891000
1	-13.492735000	4.333385000	-1.834948000
1	-15.627861000	0.050812000	2.393056000
8	-10.895759000	1.153780000	0.656566000
8	-9.095089000	-0.685273000	1.400740000
1	-8.362010000	-0.542072000	2.017127000
1	-10.170930000	0.652879000	1.106912000
1	-19.773869000	5.409659000	7.375478000
1	-11.551788000	6.600868000	9.542358000
1	-15.396159000	6.668332000	2.989631000
1	-12.153984000	-0.116333000	-2.426987000
1	-8.336167000	3.943575000	-5.394603000
1	-14.608249000	-1.236792000	4.441843000
1	-12.162769000	-1.288520000	5.619654000
1	-16.623681000	1.113346000	0.197899000
1	-15.691320000	2.880108000	-1.639238000
1	-11.423211000	5.967234000	-1.928403000
1	-9.227991000	6.322104000	-0.375897000
1	-7.111972000	3.793676000	3.679269000
1	-7.778088000	1.608534000	5.146059000
1	-17.005570000	5.089483000	7.764045000
6	-7.771029000	-0.934978000	-4.902982000
6	-8.206520000	-1.103235000	-3.483169000
7	-9.262934000	-1.929843000	-3.119637000
6	-7.760893000	-0.571676000	-2.295952000
6	-9.410027000	-1.869082000	-1.766287000
7	-8.514512000	-1.055807000	-1.239469000
1	-8.578341000	-0.538224000	-5.532078000
1	-7.431473000	-1.881878000	-5.342681000
1	-9.827981000	-2.479362000	-3.752098000
1	-6.969225000	0.146718000	-2.139176000
1	-10.184307000	-2.392461000	-1.226028000
1	-8.709716000	-0.802418000	0.477416000
1	-6.939248000	-0.226555000	-4.949362000

^{2}C*c*P S-Trp W191F Reduced CpdII

6	-12.856979000	-0.596965000	-1.738957000

7	-12.216682000	-0.904512000	-0.473125000
6	-12.871614000	-1.550676000	0.440865000
7	-14.214221000	-1.877716000	0.361863000
7	-12.200029000	-2.044750000	1.540331000
1	-13.385258000	0.365677000	-1.701834000
1	-13.574939000	-1.370936000	-2.051762000
1	-11.408275000	0.339882000	0.370347000
1	-14.812981000	-1.150885000	-0.013438000
1	-14.607762000	-2.320453000	1.182741000
1	-11.203875000	-1.827805000	1.552937000
1	-12.644516000	-1.895258000	2.440404000
6	-9.070010000	4.292997000	-4.654966000
6	-9.230588000	3.315246000	-3.536192000
6	-10.255850000	2.425165000	-3.424929000
6	-8.327053000	3.229355000	-2.410779000
16	-10.214011000	1.468842000	-1.970270000
6	-8.734249000	2.250231000	-1.468152000
6	-7.157293000	3.970305000	-2.158339000
6	-7.992113000	1.992595000	-0.311842000
6	-6.420257000	3.718862000	-1.005622000
6	-6.834547000	2.731194000	-0.090896000
1	-9.107394000	5.327726000	-4.287081000
1	-9.861844000	4.175699000	-5.401878000
1	-11.079380000	2.300860000	-4.118466000
1	-6.832294000	4.730632000	-2.864119000
1	-8.323717000	1.248958000	0.401221000
1	-5.515433000	4.287744000	-0.808424000
1	-6.256064000	2.554366000	0.811401000
6	-14.934996000	6.434015000	3.955035000
6	-13.912923000	5.351182000	3.831285000
7	-13.104860000	4.973219000	4.897054000
6	-13.626148000	4.537649000	2.761482000
6	-12.369467000	3.958482000	4.457555000
7	-12.642297000	3.646102000	3.160689000
1	-14.480552000	7.375416000	4.289274000
1	-15.706694000	6.161232000	4.683802000
1	-12.985224000	5.670805000	6.368707000
1	-14.044224000	4.519126000	1.767830000
1	-11.639469000	3.430899000	5.053190000
6	-19.303988000	5.818997000	6.472007000
6	-18.170595000	4.921368000	6.018388000
6	-17.055992000	4.700676000	6.844941000
6	-18.192065000	4.300117000	4.762282000
6	-15.994839000	3.890917000	6.433316000
6	-17.132353000	3.486673000	4.342370000
6	-16.032180000	3.280667000	5.175095000
1	-20.106140000	5.854040000	5.726890000
1	-18.955074000	6.846441000	6.637525000
1	-19.041300000	4.462819000	4.101135000
1	-15.126220000	3.763215000	7.073391000

1	-17.154791000	3.034144000	3.353525000
1	-15.192729000	2.681276000	4.836610000
6	-12.599025000	6.280971000	9.586902000
6	-12.867010000	5.472856000	8.327953000
8	-12.847260000	6.224707000	7.233694000
8	-13.070954000	4.264868000	8.336640000
1	-12.674248000	5.638077000	10.465715000
1	-13.315079000	7.106648000	9.662858000
6	-14.426378000	0.380630000	3.168013000
6	-9.851733000	1.040047000	4.622341000
6	-9.442526000	4.581402000	1.347101000
6	-13.582580000	3.154474000	-0.713600000
6	-13.237527000	0.288417000	3.881611000
6	-13.038230000	-0.566466000	5.029966000
6	-11.730185000	-0.432544000	5.399936000
6	-11.139725000	0.530870000	4.500560000
6	-9.345964000	2.094976000	3.872908000
6	-8.068491000	2.734321000	4.101652000
6	-7.984518000	3.769508000	3.217204000
6	-9.196158000	3.746988000	2.428573000
6	-10.521869000	4.475919000	0.482049000
6	-10.665820000	5.246121000	-0.734759000
6	-11.804898000	4.812567000	-1.342679000
6	-12.370726000	3.791836000	-0.486987000
6	-14.186394000	2.268615000	0.171646000
6	-15.540013000	1.778535000	0.049744000
6	-15.799778000	1.055217000	1.181050000
6	-14.592491000	1.071955000	1.974615000
7	-12.067953000	0.939715000	3.574207000
7	-10.009045000	2.727011000	2.850952000
7	-11.569338000	3.602205000	0.608474000
7	-13.625426000	1.810538000	1.338264000
26	-11.830073000	2.303927000	2.114368000
1	-9.224703000	0.636540000	5.411539000
1	-8.688356000	5.324838000	1.110906000
1	-14.132339000	3.423130000	-1.610692000
1	-15.284831000	-0.159866000	3.556296000
8	-10.881449000	0.957272000	0.968832000
8	-9.212462000	-1.150712000	1.412494000
1	-8.494250000	-1.343045000	2.032700000
1	-10.239467000	0.366783000	1.419107000
1	-19.737422000	5.468229000	7.417123000
1	-11.597533000	6.723126000	9.535148000
1	-15.425125000	6.611290000	2.991744000
1	-12.086493000	-0.512456000	-2.511973000
1	-8.105332000	4.171080000	-5.165908000
1	-13.808889000	-1.173618000	5.488509000
1	-11.212890000	-0.904756000	6.225401000
1	-16.718808000	0.557943000	1.467214000
1	-16.204960000	1.999268000	-0.776124000

1	-12.236017000	5.139302000	-2.280160000
1	-9.961439000	5.994938000	-1.072744000
1	-7.180276000	4.482318000	3.086954000
1	-7.353979000	2.432123000	4.857031000
1	-17.012542000	5.181001000	7.820870000
6	-7.771002000	-0.935017000	-4.903022000
6	-8.220629000	-1.232967000	-3.506563000
7	-9.422946000	-1.871382000	-3.232349000
6	-7.680729000	-0.978051000	-2.267088000
6	-9.564334000	-1.966472000	-1.880884000
7	-8.523333000	-1.436030000	-1.266872000
1	-8.457518000	-0.241660000	-5.405870000
1	-7.694418000	-1.844528000	-5.512614000
1	-10.093720000	-2.187430000	-3.918789000
1	-6.756869000	-0.467566000	-2.035485000
1	-10.443874000	-2.364872000	-1.397370000
1	-8.836395000	-1.254105000	0.487831000
1	-6.784519000	-0.463352000	-4.879818000

^{4}C*c*P W191F Reduced CpdII

6	-12.857001000	-0.596908000	-1.739055000
7	-12.139504000	-0.968520000	-0.535429000
6	-12.692164000	-1.734569000	0.349231000
7	-13.986444000	-2.216857000	0.259658000
7	-11.947346000	-2.216610000	1.405688000
1	-13.629699000	0.162581000	-1.545588000
1	-13.340193000	-1.458095000	-2.227552000
1	-11.172564000	0.174799000	0.085491000
1	-14.662153000	-1.579787000	-0.142188000
1	-14.338945000	-2.720240000	1.063737000
1	-10.994725000	-1.856347000	1.455004000
1	-12.407541000	-2.214007000	2.310505000
6	-9.070016000	4.292874000	-4.654976000
6	-8.865778000	3.456592000	-3.429759000
6	-9.797219000	2.658416000	-2.799967000
6	-7.671384000	3.381636000	-2.627016000
7	-9.257209000	2.083754000	-1.669493000
6	-7.961972000	2.518582000	-1.528183000
6	-6.397757000	3.974440000	-2.707139000
6	-7.020742000	2.235741000	-0.531877000
6	-5.460361000	3.700425000	-1.714345000
6	-5.769768000	2.837643000	-0.637352000
1	-8.929384000	5.361947000	-4.440913000
1	-10.082521000	4.165783000	-5.055389000
1	-10.830089000	2.472732000	-3.063956000
1	-9.776036000	1.586764000	-0.937313000
1	-6.148480000	4.639642000	-3.530703000
1	-7.267312000	1.581143000	0.299139000
1	-4.473571000	4.153063000	-1.765511000
1	-5.016713000	2.642154000	0.121939000

6	-14.935015000	6.433939000	3.954996000
6	-13.875096000	5.392581000	3.830538000
7	-13.053965000	5.026731000	4.885202000
6	-13.556338000	4.609114000	2.744492000
6	-12.281078000	4.046775000	4.413550000
7	-12.540871000	3.747130000	3.114946000
1	-14.516181000	7.399112000	4.269138000
1	-15.688782000	6.145822000	4.696195000
1	-12.946762000	5.691255000	6.351449000
1	-13.982937000	4.597368000	1.752778000
1	-11.525055000	3.538033000	4.996276000
6	-19.303977000	5.818995000	6.471994000
6	-18.175588000	4.923987000	6.000423000
6	-17.049863000	4.699449000	6.810772000
6	-18.210635000	4.313348000	4.739573000
6	-15.989625000	3.899026000	6.378404000
6	-17.152197000	3.509339000	4.299079000
6	-16.039571000	3.300728000	5.114675000
1	-20.136838000	5.817493000	5.760472000
1	-18.964885000	6.856627000	6.587975000
1	-19.069101000	4.477478000	4.090808000
1	-15.112497000	3.772461000	7.007020000
1	-17.188280000	3.065407000	3.306603000
1	-15.199341000	2.711695000	4.758626000
6	-12.598977000	6.281016000	9.586980000
6	-12.880853000	5.486040000	8.321681000
8	-12.803981000	6.234433000	7.229767000
8	-13.141805000	4.288227000	8.329396000
1	-12.723610000	5.643421000	10.464131000
1	-13.274572000	7.141277000	9.648612000
6	-14.674189000	0.753718000	1.863429000
6	-10.615743000	0.353401000	4.494817000
6	-8.635063000	3.969805000	1.941773000
6	-12.516624000	4.053918000	-0.955601000
6	-13.754391000	0.361714000	2.827954000
6	-14.013755000	-0.622510000	3.849673000
6	-12.858083000	-0.761489000	4.569703000
6	-11.898743000	0.152886000	4.000237000
6	-9.719315000	1.304774000	4.027636000
6	-8.404756000	1.535321000	4.575658000
6	-7.855725000	2.568605000	3.868666000
6	-8.830401000	2.966849000	2.883340000
6	-9.533316000	4.317748000	0.946016000
6	-9.268600000	5.302398000	-0.078313000
6	-10.356537000	5.321935000	-0.896739000
6	-11.291000000	4.348489000	-0.377421000
6	-13.426478000	3.121159000	-0.472121000
6	-14.749534000	2.913844000	-1.007103000
6	-15.370637000	2.009600000	-0.190222000
6	-14.421287000	1.647640000	0.832927000

7	-12.461334000	0.814751000	2.935088000
7	-9.955214000	2.186233000	2.998291000
7	-10.770022000	3.753893000	0.744340000
7	-13.239706000	2.323180000	0.631440000
26	-11.645468000	2.347558000	1.889941000
1	-10.305264000	-0.255385000	5.338195000
1	-7.685800000	4.494336000	1.959512000
1	-12.797916000	4.615625000	-1.840815000
1	-15.668236000	0.319882000	1.917687000
8	-10.573491000	0.883049000	0.501657000
8	-8.978795000	-1.115833000	1.410251000
1	-8.254739000	-1.079234000	2.052162000
1	-9.906938000	0.353361000	0.996103000
1	-19.691324000	5.494385000	7.445809000
1	-11.575986000	6.673144000	9.555735000
1	-15.442828000	6.578315000	2.995116000
1	-12.144780000	-0.159545000	-2.446649000
1	-8.362888000	4.034382000	-5.455254000
1	-14.965743000	-1.115520000	4.003872000
1	-12.673159000	-1.392917000	5.429604000
1	-16.381646000	1.626906000	-0.257419000
1	-15.149364000	3.420004000	-1.876704000
1	-10.518382000	5.912811000	-1.788375000
1	-8.349690000	5.867181000	-0.162622000
1	-6.884347000	3.029654000	3.994286000
1	-7.977059000	0.984543000	5.404024000
1	-16.995398000	5.170598000	7.790573000
6	-7.771013000	-0.934915000	-4.902939000
6	-8.168914000	-1.220454000	-3.491803000
7	-9.308316000	-1.946930000	-3.169601000
6	-7.616467000	-0.891261000	-2.276943000
6	-9.397417000	-2.028650000	-1.812619000
7	-8.386631000	-1.398872000	-1.243524000
1	-8.539558000	-0.356617000	-5.431863000
1	-7.584922000	-1.855446000	-5.471502000
1	-9.961771000	-2.345215000	-3.829579000
1	-6.735175000	-0.296456000	-2.084718000
1	-10.217892000	-2.505494000	-1.298497000
1	-8.575445000	-1.242083000	0.498031000
1	-6.851502000	-0.343082000	-4.912195000

^{4}C*c*P S-Trp W191F Reduced CpdII

6	-12.856997000	-0.596983000	-1.739005000
7	-12.201201000	-1.042363000	-0.524778000
6	-12.855696000	-1.742622000	0.343721000
7	-14.203763000	-2.059538000	0.246172000
7	-12.191478000	-2.328687000	1.403444000
1	-13.334113000	0.383136000	-1.604942000
1	-13.619415000	-1.304284000	-2.102137000
1	-11.133831000	0.055657000	0.291716000
1	-14.793045000	-1.314668000	-0.103172000

1	-14.602663000	-2.527790000	1.050249000
1	-11.193154000	-2.122100000	1.440797000
1	-12.645260000	-2.243639000	2.307493000
6	-9.070004000	4.293012000	-4.654980000
6	-9.187703000	3.272845000	-3.565220000
6	-10.238013000	2.417103000	-3.409477000
6	-8.199004000	3.091629000	-2.524321000
16	-10.120699000	1.386341000	-2.009182000
6	-8.581672000	2.096816000	-1.587157000
6	-6.971603000	3.756911000	-2.346609000
6	-7.777699000	1.774761000	-0.489561000
6	-6.166235000	3.431892000	-1.261188000
6	-6.570486000	2.448443000	-0.336620000
1	-9.006338000	5.310429000	-4.245316000
1	-9.932813000	4.256301000	-5.327821000
1	-11.117042000	2.355626000	-4.040759000
1	-6.660299000	4.522180000	-3.053156000
1	-8.104553000	1.029783000	0.224963000
1	-5.217804000	3.943544000	-1.120142000
1	-5.936263000	2.217718000	0.515293000
6	-14.935062000	6.434000000	3.954989000
6	-13.883520000	5.379182000	3.861070000
7	-13.082990000	5.035087000	4.936021000
6	-13.557620000	4.557810000	2.804191000
6	-12.315747000	4.030101000	4.509249000
7	-12.558378000	3.694538000	3.217554000
1	-14.512508000	7.395389000	4.275014000
1	-15.708289000	6.156244000	4.680300000
1	-13.001356000	5.715899000	6.352289000
1	-13.968993000	4.519202000	1.806740000
1	-11.579877000	3.530144000	5.123605000
6	-19.303968000	5.818998000	6.471983000
6	-18.169124000	4.919738000	6.024284000
6	-17.043129000	4.722264000	6.841247000
6	-18.197898000	4.279304000	4.778111000
6	-15.976746000	3.919436000	6.428879000
6	-17.132989000	3.472940000	4.358049000
6	-16.020060000	3.291532000	5.179519000
1	-20.144332000	5.778066000	5.770461000
1	-18.977222000	6.864807000	6.541526000
1	-19.056784000	4.421725000	4.124734000
1	-15.099377000	3.810485000	7.060465000
1	-17.164098000	3.004899000	3.376562000
1	-15.175579000	2.699517000	4.839148000
6	-12.598977000	6.281004000	9.587026000
6	-12.856499000	5.490906000	8.315201000
8	-12.884791000	6.264160000	7.241126000
8	-13.010837000	4.273781000	8.304819000
1	-12.648821000	5.621400000	10.455514000
1	-13.334247000	7.087283000	9.684813000

6	-14.791831000	1.020202000	1.524899000
6	-11.275226000	0.076020000	4.730610000
6	-8.553099000	3.550379000	2.722114000
6	-11.851702000	4.109970000	-0.781005000
6	-14.088147000	0.492490000	2.599812000
6	-14.615131000	-0.480663000	3.525277000
6	-13.609813000	-0.777712000	4.404619000
6	-12.475229000	0.033093000	4.030752000
6	-10.222454000	0.939685000	4.460321000
6	-9.013832000	1.030513000	5.243308000
6	-8.257453000	2.025693000	4.691828000
6	-8.996188000	2.537883000	3.563237000
6	-9.225984000	4.011713000	1.600364000
6	-8.714438000	5.020763000	0.701090000
6	-9.629432000	5.158411000	-0.298295000
6	-10.711625000	4.246170000	-0.005741000
6	-12.924236000	3.277512000	-0.486083000
6	-14.160756000	3.246703000	-1.224941000
6	-15.004180000	2.403105000	-0.554355000
6	-14.275685000	1.899571000	0.582710000
7	-12.785110000	0.782767000	2.922784000
7	-10.187066000	1.863731000	3.442323000
7	-10.441164000	3.559423000	1.151534000
7	-13.006191000	2.430764000	0.593605000
26	-11.635471000	2.223154000	2.073461000
1	-11.173964000	-0.581540000	5.588242000
1	-7.591097000	4.000332000	2.945783000
1	-11.923172000	4.721136000	-1.674465000
1	-15.827606000	0.714238000	1.408421000
8	-10.546445000	0.639587000	0.855657000
8	-9.149655000	-1.667553000	1.379137000
1	-8.460339000	-2.026200000	1.956737000
1	-9.992364000	0.005531000	1.353659000
1	-19.676505000	5.529735000	7.462519000
1	-11.609263000	6.749288000	9.534930000
1	-15.419837000	6.579932000	2.983339000
1	-12.103451000	-0.482877000	-2.525042000
1	-8.165917000	4.136954000	-5.258926000
1	-15.629373000	-0.860438000	3.510599000
1	-13.636019000	-1.447705000	5.254867000
1	-16.028617000	2.146278000	-0.794120000
1	-14.357210000	3.821383000	-2.121305000
1	-9.588639000	5.802899000	-1.166328000
1	-7.764259000	5.523985000	0.818715000
1	-7.290580000	2.392084000	5.012733000
1	-8.794824000	0.421019000	6.110852000
1	-16.993807000	5.216944000	7.809669000
6	-7.770991000	-0.935032000	-4.903013000
6	-8.226131000	-1.339007000	-3.537719000
7	-9.460849000	-1.929741000	-3.307671000

6	-7.664357000	-1.224595000	-2.287860000
6	-9.600667000	-2.131542000	-1.968005000
7	-8.526509000	-1.717257000	-1.322084000
1	-8.415132000	-0.151862000	-5.323795000
1	-7.759954000	-1.781988000	-5.601128000
1	-10.156399000	-2.136998000	-4.010624000
1	-6.710153000	-0.791024000	-2.025010000
1	-10.498727000	-2.520936000	-1.512608000
1	-8.792112000	-1.703882000	0.442696000
1	-6.755572000	-0.532249000	-4.849112000

[2]C*c*P W191F Ferric-OH

6	-12.857005000	-0.597012000	-1.738980000
7	-12.248169000	-0.722823000	-0.419547000
6	-12.662020000	-1.595566000	0.482625000
7	-13.796496000	-2.316479000	0.285222000
7	-11.908609000	-1.861020000	1.568700000
1	-13.831185000	-0.093840000	-1.696316000
1	-12.970677000	-1.581455000	-2.204434000
1	-11.643391000	0.085657000	-0.042326000
1	-14.562561000	-1.876774000	-0.208052000
1	-14.057765000	-3.004879000	0.978069000
1	-11.007444000	-1.388417000	1.666456000
1	-12.381505000	-2.037446000	2.448376000
6	-9.069995000	4.293002000	-4.655011000
6	-9.001638000	3.671299000	-3.291323000
6	-9.967256000	2.915062000	-2.656715000
6	-7.915098000	3.784184000	-2.353496000
7	-9.542803000	2.523932000	-1.401302000
6	-8.297059000	3.059922000	-1.179652000
6	-6.652661000	4.405923000	-2.392807000
6	-7.440394000	2.922571000	-0.078979000
6	-5.800692000	4.279997000	-1.297144000
6	-6.188099000	3.537834000	-0.152657000
1	-9.010631000	5.387696000	-4.599050000
1	-10.008277000	4.038488000	-5.159112000
1	-10.956418000	2.644009000	-3.001569000
1	-10.123022000	2.071002000	-0.655663000
1	-6.345794000	4.977983000	-3.264697000
1	-7.747696000	2.376607000	0.806958000
1	-4.829619000	4.767077000	-1.308864000
1	-5.513701000	3.472811000	0.696665000
6	-14.934975000	6.433999000	3.955042000
6	-13.937233000	5.340513000	3.798277000
7	-13.166440000	4.886715000	4.861834000
6	-13.628436000	4.597001000	2.685692000
6	-12.426684000	3.898161000	4.376888000
7	-12.663520000	3.671991000	3.050158000
1	-14.465490000	7.351395000	4.332279000

1	-15.719450000	6.151741000	4.665935000
1	-13.182129000	5.535955000	6.439198000
1	-14.022148000	4.648901000	1.683157000
1	-11.713091000	3.338870000	4.962980000
6	-19.304020000	5.819009000	6.471998000
6	-18.220687000	4.916262000	5.920145000
6	-17.097145000	4.588393000	6.697040000
6	-18.301910000	4.387344000	4.624634000
6	-16.089349000	3.761717000	6.197577000
6	-17.292961000	3.559322000	4.118334000
6	-16.182455000	3.243916000	4.902135000
1	-20.043569000	6.069210000	5.704155000
1	-18.882685000	6.756753000	6.853856000
1	-19.158320000	4.634957000	4.000824000
1	-15.212686000	3.552334000	6.802911000
1	-17.364633000	3.181156000	3.100923000
1	-15.377184000	2.637182000	4.498171000
6	-12.599011000	6.280993000	9.586959000
6	-12.498175000	5.518298000	8.274343000
8	-13.278925000	6.036285000	7.326141000
8	-11.780558000	4.540705000	8.109410000
1	-11.968936000	5.806628000	10.340989000
1	-13.639844000	6.303090000	9.927866000
6	-14.425015000	0.417443000	2.564619000
6	-10.023069000	1.047994000	4.497787000
6	-9.460397000	4.901011000	1.619199000
6	-13.364670000	3.600530000	-0.928496000
6	-13.317162000	0.310855000	3.394121000
6	-13.231047000	-0.582110000	4.529672000
6	-11.968868000	-0.454099000	5.038408000
6	-11.297512000	0.543038000	4.233884000
6	-9.460396000	2.149627000	3.855024000
6	-8.241221000	2.811143000	4.273665000
6	-8.118486000	3.927170000	3.500507000
6	-9.243510000	3.933789000	2.589181000
6	-10.478052000	4.883549000	0.672013000
6	-10.592485000	5.827344000	-0.419753000
6	-11.661885000	5.434885000	-1.167523000
6	-12.216004000	4.265150000	-0.518909000
6	-13.991398000	2.583782000	-0.213150000
6	-15.296706000	2.044146000	-0.523423000
6	-15.619333000	1.189962000	0.494171000
6	-14.498662000	1.189804000	1.409033000
7	-12.123989000	0.972310000	3.228166000
7	-10.032482000	2.831669000	2.812052000
7	-11.472928000	3.948162000	0.588721000
7	-13.521213000	2.034177000	0.952391000
26	-11.786855000	2.453401000	1.893667000
1	-9.489653000	0.640284000	5.351641000
1	-8.744597000	5.715019000	1.562692000

1	-13.861423000	3.963009000	-1.823707000
1	-15.301903000	-0.166873000	2.828754000
8	-10.858138000	1.250115000	0.621084000
8	-9.228245000	-0.737529000	2.071353000
1	-9.310788000	-0.262460000	2.925246000
1	-10.115824000	0.825910000	1.081419000
1	-19.835034000	5.339393000	7.304256000
1	-12.283487000	7.319427000	9.436797000
1	-15.412346000	6.661649000	2.996050000
1	-12.192593000	0.012962000	-2.354873000
1	-8.244616000	3.959346000	-5.298356000
1	-14.045202000	-1.193766000	4.899220000
1	-11.541369000	-0.940151000	5.906277000
1	-16.536682000	0.631995000	0.638847000
1	-15.895279000	2.324592000	-1.381277000
1	-12.060052000	5.891005000	-2.064925000
1	-9.927301000	6.665652000	-0.581630000
1	-7.353729000	4.692482000	3.539448000
1	-7.606978000	2.481109000	5.086630000
1	-17.001564000	5.001288000	7.699023000
6	-7.770995000	-0.934990000	-4.903008000
6	-6.927184000	-0.143102000	-3.965222000
7	-5.543157000	-0.030010000	-4.083606000
6	-7.262836000	0.614828000	-2.877300000
6	-5.059668000	0.764184000	-3.113570000
7	-6.094093000	1.154340000	-2.375970000
1	-7.496872000	-1.996377000	-4.897438000
1	-7.684462000	-0.563761000	-5.930839000
1	-4.977047000	-0.474572000	-4.796677000
1	-8.224020000	0.838567000	-2.439577000
1	-4.027472000	1.042613000	-2.967322000
1	-6.048883000	1.808555000	-1.583764000
1	-8.818790000	-0.854370000	-4.604509000
1	-8.479180000	-1.344930000	2.170444000

^{2}C*c*P S-Trp W191F Ferric-OH

6	-12.857044000	-0.597012000	-1.739071000
7	-12.280083000	-0.818921000	-0.418663000
6	-12.867235000	-1.542940000	0.533450000
7	-14.128509000	-2.017094000	0.385188000
7	-12.151280000	-1.886326000	1.617968000
1	-13.496177000	0.292318000	-1.754437000
1	-13.442109000	-1.469250000	-2.046725000
1	-11.661726000	-0.050741000	0.006993000
1	-14.801184000	-1.396096000	-0.052556000
1	-14.507390000	-2.579238000	1.137117000
1	-11.172241000	-1.596616000	1.652372000
1	-12.618067000	-1.949044000	2.517427000
6	-9.070025000	4.293030000	-4.654954000
6	-9.199198000	3.296092000	-3.556047000

6	-10.189731000	2.369659000	-3.475769000
6	-8.296565000	3.233153000	-2.425149000
16	-10.102679000	1.382605000	-2.040132000
6	-8.657549000	2.225225000	-1.498590000
6	-7.173309000	4.034208000	-2.153718000
6	-7.926186000	2.008437000	-0.326229000
6	-6.440844000	3.820012000	-0.990461000
6	-6.816518000	2.813764000	-0.082485000
1	-9.145225000	5.317494000	-4.267527000
1	-9.852391000	4.163117000	-5.408516000
1	-11.008678000	2.226651000	-4.169911000
1	-6.885007000	4.817708000	-2.848721000
1	-8.224479000	1.258875000	0.397379000
1	-5.575053000	4.439614000	-0.776383000
1	-6.250684000	2.672606000	0.833512000
6	-14.934970000	6.434026000	3.954946000
6	-13.955788000	5.310242000	3.838151000
7	-13.229836000	4.862831000	4.936834000
6	-13.634660000	4.521699000	2.758629000
6	-12.512416000	3.833813000	4.513635000
7	-12.716021000	3.572277000	3.186273000
1	-14.459584000	7.330879000	4.370296000
1	-15.761302000	6.159767000	4.620475000
1	-13.251780000	5.557646000	6.457765000
1	-13.992453000	4.552865000	1.741629000
1	-11.835872000	3.269205000	5.138446000
6	-19.304003000	5.818955000	6.472012000
6	-18.216620000	4.905966000	5.947067000
6	-17.099045000	4.595888000	6.739748000
6	-18.289039000	4.348236000	4.663154000
6	-16.088418000	3.757403000	6.267290000
6	-17.276791000	3.508346000	4.183589000
6	-16.173215000	3.210234000	4.983456000
1	-20.035624000	6.057107000	5.692835000
1	-18.885493000	6.762410000	6.842596000
1	-19.140976000	4.581986000	4.027994000
1	-15.217284000	3.559052000	6.884133000
1	-17.340256000	3.107096000	3.174381000
1	-15.366688000	2.592097000	4.601092000
6	-12.598975000	6.281025000	9.587021000
6	-12.585530000	5.494516000	8.288676000
8	-13.309207000	6.081088000	7.336422000
8	-11.976567000	4.443780000	8.132806000
1	-12.047493000	5.739476000	10.357319000
1	-13.630681000	6.451593000	9.912835000
6	-14.575434000	0.350205000	2.939553000
6	-10.063824000	0.802066000	4.650977000
6	-9.406933000	4.527722000	1.629325000
6	-13.406803000	3.238105000	-0.775951000
6	-13.435091000	0.202120000	3.721539000

6	-13.315247000	-0.720532000	4.829729000
6	-12.018814000	-0.654359000	5.259684000
6	-11.358567000	0.336725000	4.440465000
6	-9.498827000	1.891641000	3.998707000
6	-8.220610000	2.484413000	4.334290000
6	-8.070233000	3.572312000	3.526224000
6	-9.240189000	3.627260000	2.674841000
6	-10.425066000	4.483583000	0.686573000
6	-10.495897000	5.325057000	-0.486955000
6	-11.591437000	4.926206000	-1.194739000
6	-12.208751000	3.856771000	-0.442233000
6	-14.083489000	2.327748000	0.030987000
6	-15.424846000	1.848976000	-0.217790000
6	-15.780295000	1.091222000	0.865047000
6	-14.643554000	1.079751000	1.757963000
7	-12.231388000	0.823908000	3.498749000
7	-10.095781000	2.604497000	2.990404000
7	-11.472989000	3.594968000	0.688133000
7	-13.623443000	1.829105000	1.225402000
26	-11.879416000	2.252588000	2.139914000
1	-9.489589000	0.341858000	5.449360000
1	-8.639793000	5.283904000	1.497325000
1	-13.896586000	3.556913000	-1.691332000
1	-15.470712000	-0.178389000	3.254443000
8	-10.895224000	0.936244000	0.953018000
8	-9.250346000	-0.904151000	1.292433000
1	-8.749950000	-0.869739000	2.122951000
1	-9.847134000	-0.038961000	1.254679000
1	-19.843795000	5.353768000	7.306765000
1	-12.142324000	7.264123000	9.426255000
1	-15.354815000	6.686042000	2.975524000
1	-12.045548000	-0.450559000	-2.454108000
1	-8.098774000	4.213934000	-5.160202000
1	-14.127100000	-1.313451000	5.232959000
1	-11.557737000	-1.182708000	6.084472000
1	-16.729170000	0.607767000	1.065289000
1	-16.025185000	2.108349000	-1.081240000
1	-11.970440000	5.322404000	-2.128105000
1	-9.786777000	6.107028000	-0.725663000
1	-7.252066000	4.280621000	3.495809000
1	-7.557820000	2.125434000	5.111414000
1	-17.011522000	5.031188000	7.732865000
6	-7.770983000	-0.935024000	-4.902954000
6	-8.190370000	-1.236767000	-3.497194000
7	-9.352148000	-1.949898000	-3.209163000
6	-7.677359000	-0.887590000	-2.273951000
6	-9.542946000	-2.013912000	-1.882208000
7	-8.529118000	-1.382201000	-1.300046000
1	-8.496302000	-0.271419000	-5.387625000
1	-7.675097000	-1.848517000	-5.500272000

1	-9.966846000	-2.362846000	-3.899773000
1	-6.810821000	-0.296729000	-2.023340000
1	-10.378742000	-2.464897000	-1.371300000
1	-8.535248000	-1.193258000	-0.268478000
1	-6.802811000	-0.428945000	-4.899989000
1	-10.569797000	1.612122000	0.338970000

^{4}C*c*P W191F Ferric-OH

6	-12.857001000	-0.597013000	-1.738979000
7	-12.272376000	-0.753134000	-0.413349000
6	-12.770306000	-1.558817000	0.510790000
7	-13.970775000	-2.167352000	0.325054000
7	-12.043814000	-1.859635000	1.603400000
1	-13.777464000	-0.001847000	-1.714734000
1	-13.061329000	-1.576751000	-2.182430000
1	-11.653354000	0.033743000	-0.044867000
1	-14.695889000	-1.625931000	-0.131636000
1	-14.295333000	-2.802108000	1.042512000
1	-11.098025000	-1.476247000	1.681117000
1	-12.527134000	-1.974719000	2.487599000
6	-9.069996000	4.293001000	-4.655010000
6	-9.011204000	3.676668000	-3.289645000
6	-9.986855000	2.928879000	-2.661578000
6	-7.932851000	3.790640000	-2.342176000
7	-9.575753000	2.545308000	-1.400181000
6	-8.331322000	3.078262000	-1.166458000
6	-6.665061000	4.401711000	-2.373274000
6	-7.490329000	2.947007000	-0.052849000
6	-5.826441000	4.279318000	-1.266701000
6	-6.233270000	3.553918000	-0.118741000
1	-9.019458000	5.388432000	-4.602526000
1	-10.001668000	4.029674000	-5.166856000
1	-10.974518000	2.660241000	-3.012073000
1	-10.160165000	2.091723000	-0.664669000
1	-6.343629000	4.962699000	-3.247147000
1	-7.810585000	2.416326000	0.837681000
1	-4.851356000	4.758543000	-1.272499000
1	-5.571544000	3.494975000	0.740810000
6	-14.934982000	6.433999000	3.955112000
6	-13.945449000	5.340733000	3.820571000
7	-13.065463000	4.999036000	4.802008000
6	-13.713964000	4.489777000	2.702172000
6	-12.341433000	3.991351000	4.301249000
7	-12.703386000	3.657875000	3.019759000
1	-14.443083000	7.365834000	4.260391000
1	-15.673549000	6.189215000	4.729147000
1	-12.888600000	5.741226000	6.383687000
1	-14.212451000	4.460141000	1.745198000
1	-11.546741000	3.490177000	4.832767000
6	-19.304010000	5.819012000	6.471976000

6	-18.162561000	4.964442000	5.957574000
6	-17.038932000	4.702572000	6.762689000
6	-18.203822000	4.386337000	4.679527000
6	-16.001588000	3.878202000	6.320379000
6	-17.168439000	3.560269000	4.227514000
6	-16.067038000	3.299097000	5.047770000
1	-19.936588000	6.176518000	5.652855000
1	-18.933516000	6.690236000	7.023922000
1	-19.056357000	4.582963000	4.032861000
1	-15.135065000	3.712441000	6.954506000
1	-17.217550000	3.126968000	3.231020000
1	-15.260376000	2.662605000	4.695208000
6	-12.599016000	6.280992000	9.586911000
6	-12.868683000	5.513243000	8.310070000
8	-12.731803000	6.279452000	7.221606000
8	-13.172217000	4.330194000	8.269707000
1	-12.754976000	5.632472000	10.450145000
1	-13.260035000	7.152320000	9.647592000
6	-14.472918000	0.462557000	2.687556000
6	-9.928802000	0.943016000	4.320484000
6	-9.637121000	4.964778000	1.568818000
6	-13.408841000	3.416358000	-1.038094000
6	-13.352167000	0.361735000	3.476533000
6	-13.217809000	-0.513211000	4.618688000
6	-11.914699000	-0.451755000	5.023439000
6	-11.248851000	0.510531000	4.165188000
6	-9.405753000	2.097508000	3.690864000
6	-8.202825000	2.774186000	4.068393000
6	-8.171385000	3.956970000	3.347930000
6	-9.328888000	3.965943000	2.520251000
6	-10.639877000	4.914731000	0.634098000
6	-10.798916000	5.837626000	-0.469082000
6	-11.807619000	5.359439000	-1.249239000
6	-12.310146000	4.158205000	-0.604753000
6	-14.042288000	2.426198000	-0.264647000
6	-15.337106000	1.874881000	-0.530547000
6	-15.670532000	1.096461000	0.565450000
6	-14.566021000	1.159239000	1.466350000
7	-12.124344000	0.976431000	3.223012000
7	-10.063331000	2.815992000	2.723687000
7	-11.593943000	3.906877000	0.526634000
7	-13.573063000	1.958628000	0.942058000
26	-11.817538000	2.391171000	1.830360000
1	-9.353782000	0.516115000	5.137087000
1	-8.961637000	5.814209000	1.525639000
1	-13.896939000	3.724981000	-1.957363000
1	-15.355919000	-0.084767000	3.007555000
8	-10.883184000	1.237090000	0.604569000
8	-9.317366000	-0.841835000	1.970474000
1	-9.371247000	-0.361100000	2.831462000

1	-10.139768000	0.821085000	1.072197000
1	-19.941199000	5.245950000	7.158253000
1	-11.569038000	6.654636000	9.583396000
1	-15.466890000	6.603334000	3.014947000
1	-12.131045000	-0.069113000	-2.361443000
1	-8.236316000	3.964060000	-5.290166000
1	-14.027093000	-1.087740000	5.053372000
1	-11.447518000	-0.959479000	5.857818000
1	-16.593884000	0.559159000	0.748008000
1	-15.940619000	2.097082000	-1.401806000
1	-12.201823000	5.775217000	-2.167856000
1	-10.187473000	6.717316000	-0.626005000
1	-7.425314000	4.740818000	3.381228000
1	-7.504088000	2.429194000	4.820020000
1	-16.976874000	5.150756000	7.752190000
6	-7.770995000	-0.934991000	-4.903009000
6	-6.952399000	-0.154778000	-3.932531000
7	-5.563179000	-0.062588000	-3.999251000
6	-7.316162000	0.608159000	-2.856834000
6	-5.103873000	0.724486000	-3.011718000
7	-6.158678000	1.130299000	-2.312689000
1	-7.523365000	-2.002562000	-4.875982000
1	-7.627277000	-0.575089000	-5.928420000
1	-4.978015000	-0.515477000	-4.691277000
1	-8.288542000	0.847008000	-2.453195000
1	-4.073880000	0.988156000	-2.827749000
1	-6.133591000	1.784678000	-1.519195000
1	-8.829360000	-0.826450000	-4.654933000
1	-8.553898000	-1.435323000	2.038236000

^{4}C*c*P S-Trp W191F Ferric-OH

6	-12.857044000	-0.597012000	-1.739071000
7	-12.280083000	-0.818921000	-0.418663000
6	-12.867235000	-1.542940000	0.533450000
7	-14.128509000	-2.017094000	0.385188000
7	-12.151280000	-1.886326000	1.617968000
1	-13.496177000	0.292318000	-1.754437000
1	-13.442109000	-1.469250000	-2.046725000
1	-11.661726000	-0.050741000	0.006993000
1	-14.801184000	-1.396096000	-0.052556000
1	-14.507390000	-2.579238000	1.137117000
1	-11.172241000	-1.596616000	1.652372000
1	-12.618067000	-1.949044000	2.517427000
6	-9.070025000	4.293030000	-4.654954000
6	-9.199198000	3.296092000	-3.556047000
6	-10.189731000	2.369659000	-3.475769000
6	-8.296565000	3.233153000	-2.425149000
16	-10.102679000	1.382605000	-2.040132000
6	-8.657549000	2.225225000	-1.498590000
6	-7.173309000	4.034208000	-2.153718000

6	-7.926186000	2.008437000	-0.326229000
6	-6.440844000	3.820012000	-0.990461000
6	-6.816518000	2.813764000	-0.082485000
1	-9.145225000	5.317494000	-4.267527000
1	-9.852391000	4.163117000	-5.408516000
1	-11.008678000	2.226651000	-4.169911000
1	-6.885007000	4.817708000	-2.848721000
1	-8.224479000	1.258875000	0.397379000
1	-5.575053000	4.439614000	-0.776383000
1	-6.250684000	2.672606000	0.833512000
6	-14.934970000	6.434026000	3.954946000
6	-13.955788000	5.310242000	3.838151000
7	-13.229836000	4.862831000	4.936834000
6	-13.634660000	4.521699000	2.758629000
6	-12.512416000	3.833813000	4.513635000
7	-12.716021000	3.572277000	3.186273000
1	-14.459584000	7.330879000	4.370296000
1	-15.761302000	6.159767000	4.620475000
1	-13.251780000	5.557646000	6.457765000
1	-13.992453000	4.552865000	1.741629000
1	-11.835872000	3.269205000	5.138446000
6	-19.304003000	5.818955000	6.472012000
6	-18.216620000	4.905966000	5.947067000
6	-17.099045000	4.595888000	6.739748000
6	-18.289039000	4.348236000	4.663154000
6	-16.088418000	3.757403000	6.267290000
6	-17.276791000	3.508346000	4.183589000
6	-16.173215000	3.210234000	4.983456000
1	-20.035624000	6.057107000	5.692835000
1	-18.885493000	6.762410000	6.842596000
1	-19.140976000	4.581986000	4.027994000
1	-15.217284000	3.559052000	6.884133000
1	-17.340256000	3.107096000	3.174381000
1	-15.366688000	2.592097000	4.601092000
6	-12.598975000	6.281025000	9.587021000
6	-12.585530000	5.494516000	8.288676000
8	-13.309207000	6.081088000	7.336422000
8	-11.976567000	4.443780000	8.132806000
1	-12.047493000	5.739476000	10.357319000
1	-13.630681000	6.451593000	9.912835000
6	-14.575434000	0.350205000	2.939553000
6	-10.063824000	0.802066000	4.650977000
6	-9.406933000	4.527722000	1.629325000
6	-13.406803000	3.238105000	-0.775951000
6	-13.435091000	0.202120000	3.721539000
6	-13.315247000	-0.720532000	4.829729000
6	-12.018814000	-0.654359000	5.259684000
6	-11.358567000	0.336725000	4.440465000
6	-9.498827000	1.891641000	3.998707000
6	-8.220610000	2.484413000	4.334290000

6	-8.070233000	3.572312000	3.526224000
6	-9.240189000	3.627260000	2.674841000
6	-10.425066000	4.483583000	0.686573000
6	-10.495897000	5.325057000	-0.486955000
6	-11.591437000	4.926206000	-1.194739000
6	-12.208751000	3.856771000	-0.442233000
6	-14.083489000	2.327748000	0.030987000
6	-15.424846000	1.848976000	-0.217790000
6	-15.780295000	1.091222000	0.865047000
6	-14.643554000	1.079751000	1.757963000
7	-12.231388000	0.823908000	3.498749000
7	-10.095781000	2.604497000	2.990404000
7	-11.472989000	3.594968000	0.688133000
7	-13.623443000	1.829105000	1.225402000
26	-11.879416000	2.252588000	2.139914000
1	-9.489589000	0.341858000	5.449360000
1	-8.639793000	5.283904000	1.497325000
1	-13.896586000	3.556913000	-1.691332000
1	-15.470712000	-0.178389000	3.254443000
8	-10.895224000	0.936244000	0.953018000
8	-9.250346000	-0.904151000	1.292433000
1	-8.749950000	-0.869739000	2.122951000
1	-9.847134000	-0.038961000	1.254679000
1	-19.843795000	5.353768000	7.306765000
1	-12.142324000	7.264123000	9.426255000
1	-15.354815000	6.686042000	2.975524000
1	-12.045548000	-0.450559000	-2.454108000
1	-8.098774000	4.213934000	-5.160202000
1	-14.127100000	-1.313451000	5.232959000
1	-11.557737000	-1.182708000	6.084472000
1	-16.729170000	0.607767000	1.065289000
1	-16.025185000	2.108349000	-1.081240000
1	-11.970440000	5.322404000	-2.128105000
1	-9.786777000	6.107028000	-0.725663000
1	-7.252066000	4.280621000	3.495809000
1	-7.557820000	2.125434000	5.111414000
1	-17.011522000	5.031188000	7.732865000
6	-7.770983000	-0.935024000	-4.902954000
6	-8.190370000	-1.236767000	-3.497194000
7	-9.352148000	-1.949898000	-3.209163000
6	-7.677359000	-0.887590000	-2.273951000
6	-9.542946000	-2.013912000	-1.882208000
7	-8.529118000	-1.382201000	-1.300046000
1	-8.496302000	-0.271419000	-5.387625000
1	-7.675097000	-1.848517000	-5.500272000
1	-9.966846000	-2.362846000	-3.899773000
1	-6.810821000	-0.296729000	-2.023340000
1	-10.378742000	-2.464897000	-1.371300000
1	-8.535248000	-1.193258000	-0.268478000
1	-6.802811000	-0.428945000	-4.899989000

1	-10.569797000	1.612122000	0.338970000

[2]C*c*P SO-Trp W191F

6	-12.856784000	-0.596598000	-1.739238000
7	-12.030964000	-0.718999000	-0.542939000
6	-12.324744000	-1.517650000	0.485526000
7	-13.463943000	-2.235053000	0.489660000
7	-11.417401000	-1.711862000	1.459273000
1	-13.753139000	0.008737000	-1.556449000
1	-13.141931000	-1.586026000	-2.108958000
1	-11.430405000	0.097294000	-0.347294000
1	-14.281717000	-1.878055000	0.013265000
1	-13.632962000	-2.897092000	1.235970000
1	-10.548330000	-1.170841000	1.413928000
1	-11.766355000	-1.832278000	2.407326000
6	-9.070440000	4.292546000	-4.654532000
6	-9.088927000	3.515051000	-3.372599000
6	-10.103273000	2.725542000	-2.946383000
6	-7.923992000	3.487367000	-2.468907000
16	-9.570085000	1.625087000	-1.652325000
6	-8.083798000	2.586199000	-1.402980000
6	-6.745048000	4.230672000	-2.547621000
6	-7.139146000	2.416648000	-0.403719000
6	-5.780061000	4.082488000	-1.543306000
6	-5.978010000	3.197639000	-0.477612000
1	-8.880939000	5.353818000	-4.456839000
1	-10.018502000	4.207936000	-5.190541000
1	-11.061113000	2.548525000	-3.417368000
1	-6.581266000	4.924300000	-3.366445000
1	-7.303831000	1.745878000	0.432363000
1	-4.868494000	4.670410000	-1.588404000
1	-5.234232000	3.120374000	0.308923000
6	-14.934754000	6.434517000	3.955105000
6	-13.838351000	5.505158000	3.543485000
7	-12.925653000	5.009937000	4.457266000
6	-13.457719000	4.961437000	2.335623000
6	-12.046851000	4.208220000	3.812725000
7	-12.343625000	4.155184000	2.518186000
1	-14.531102000	7.400176000	4.284287000
1	-15.524587000	6.013497000	4.773506000
1	-12.940282000	5.201351000	5.458514000
1	-13.905678000	5.087315000	1.362232000
1	-11.237363000	3.688696000	4.298750000
6	-19.303967000	5.818638000	6.471905000
6	-18.208956000	4.962561000	5.870780000
6	-17.127632000	4.521177000	6.652371000
6	-18.253153000	4.562024000	4.527037000
6	-16.132791000	3.699634000	6.114658000
6	-17.257955000	3.743485000	3.982489000
6	-16.193315000	3.306303000	4.774507000

1	-19.884995000	6.327244000	5.696290000
1	-18.893227000	6.579348000	7.144898000
1	-19.076548000	4.895607000	3.899783000
1	-15.303690000	3.380078000	6.740440000
1	-17.309744000	3.457410000	2.934687000
1	-15.409346000	2.686313000	4.346923000
6	-12.599071000	6.280943000	9.586763000
6	-12.427941000	5.661890000	8.241440000
8	-13.295165000	5.679250000	7.322781000
8	-11.371410000	5.062928000	7.867117000
1	-11.979109000	5.767752000	10.327220000
1	-13.652286000	6.249613000	9.880225000
6	-14.092965000	0.922856000	1.865537000
6	-9.646436000	1.474514000	3.728070000
6	-8.912621000	5.203443000	0.715398000
6	-13.077898000	4.206123000	-1.548675000
6	-12.969792000	0.780337000	2.674910000
6	-12.900051000	-0.071983000	3.842867000
6	-11.628977000	0.043169000	4.340218000
6	-10.936126000	0.988833000	3.489985000
6	-9.043394000	2.534708000	3.050066000
6	-7.752666000	3.103330000	3.394189000
6	-7.568584000	4.176466000	2.573727000
6	-8.733879000	4.254850000	1.715474000
6	-9.985870000	5.261446000	-0.168248000
6	-10.102725000	6.206103000	-1.262172000
6	-11.266420000	5.915096000	-1.910254000
6	-11.865416000	4.796626000	-1.209316000
6	-13.706178000	3.178173000	-0.846913000
6	-15.037718000	2.681817000	-1.130119000
6	-15.340986000	1.788514000	-0.141902000
6	-14.184268000	1.722455000	0.728967000
7	-11.759582000	1.404934000	2.471096000
7	-9.612633000	3.241631000	2.014915000
7	-11.067176000	4.416462000	-0.155236000
7	-13.198375000	2.563832000	0.272096000
26	-11.438346000	2.964404000	1.182362000
1	-9.108019000	1.058949000	4.576095000
1	-8.130322000	5.946890000	0.594054000
1	-13.608810000	4.619171000	-2.401987000
1	-14.981316000	0.364787000	2.150568000
8	-10.431105000	1.535107000	-0.352840000
8	-8.928477000	-0.174876000	1.158664000
1	-8.728074000	0.103351000	2.074184000
1	-9.441775000	0.588120000	0.792703000
1	-19.999933000	5.205489000	7.058716000
1	-12.284344000	7.330354000	9.535311000
1	-15.607450000	6.617996000	3.113483000
1	-12.263390000	-0.094352000	-2.505981000
1	-8.265560000	3.931202000	-5.306340000

1	-13.727969000	-0.642453000	4.247672000
1	-11.212267000	-0.417674000	5.227612000
1	-16.265723000	1.244182000	0.008349000
1	-15.662984000	3.011443000	-1.950609000
1	-11.700634000	6.413738000	-2.768002000
1	-9.389523000	6.991330000	-1.480127000
1	-6.734414000	4.866384000	2.548717000
1	-7.105196000	2.741550000	4.183409000
1	-17.067414000	4.823085000	7.695656000
6	-7.770984000	-0.935045000	-4.903002000
6	-7.996961000	-1.188643000	-3.450445000
7	-9.047574000	-1.977875000	-2.985745000
6	-7.368950000	-0.728781000	-2.322988000
6	-9.067433000	-1.988194000	-1.645246000
7	-8.048810000	-1.239072000	-1.229192000
1	-8.627293000	-0.415448000	-5.348317000
1	-7.608552000	-1.867630000	-5.455033000
1	-9.683418000	-2.503516000	-3.575156000
1	-6.523787000	-0.067888000	-2.211434000
1	-9.778354000	-2.499952000	-1.015998000
1	-7.957164000	-0.945448000	-0.244940000
1	-6.886072000	-0.308786000	-5.038002000

^{4}C*c*P SO-Trp W191F

6	-12.856814000	-0.596627000	-1.739290000
7	-11.995694000	-0.769623000	-0.574404000
6	-12.279936000	-1.569862000	0.453516000
7	-13.438736000	-2.254671000	0.485040000
7	-11.351484000	-1.799040000	1.400464000
1	-13.728413000	0.029591000	-1.514812000
1	-13.183389000	-1.568401000	-2.121947000
1	-11.320744000	-0.005195000	-0.414661000
1	-14.257310000	-1.877730000	0.026109000
1	-13.608933000	-2.914212000	1.233240000
1	-10.471295000	-1.276230000	1.349035000
1	-11.679574000	-1.948179000	2.351356000
6	-9.070426000	4.292544000	-4.654405000
6	-9.017109000	3.432694000	-3.430237000
6	-9.992904000	2.586394000	-3.026067000
6	-7.826474000	3.390232000	-2.560642000
16	-9.408296000	1.444502000	-1.791934000
6	-7.932091000	2.424134000	-1.546839000
6	-6.677233000	4.179912000	-2.617972000
6	-6.964156000	2.235625000	-0.573683000
6	-5.687891000	4.011302000	-1.640433000
6	-5.832921000	3.061808000	-0.623112000
1	-8.905123000	5.343837000	-4.392055000
1	-10.034525000	4.212888000	-5.161854000
1	-10.956094000	2.408900000	-3.486406000
1	-6.553984000	4.923888000	-3.398896000

1	-7.087160000	1.511156000	0.224900000
1	-4.798339000	4.632982000	-1.669166000
1	-5.069193000	2.966339000	0.142200000
6	-14.934943000	6.434510000	3.955010000
6	-13.819314000	5.494812000	3.653072000
7	-12.986200000	4.990476000	4.634706000
6	-13.361166000	4.944286000	2.479178000
6	-12.078178000	4.175154000	4.053940000
7	-12.277243000	4.122309000	2.739392000
1	-14.557236000	7.389954000	4.339499000
1	-15.616794000	6.018566000	4.701432000
1	-13.063028000	5.200833000	5.639000000
1	-13.738679000	5.077996000	1.476274000
1	-11.309172000	3.650146000	4.600644000
6	-19.303770000	5.818643000	6.471911000
6	-18.208518000	4.949466000	5.891210000
6	-17.093352000	4.583820000	6.663064000
6	-18.283135000	4.467645000	4.575709000
6	-16.092572000	3.758266000	6.144376000
6	-17.282309000	3.644009000	4.049563000
6	-16.182670000	3.284193000	4.832232000
1	-19.915464000	6.270628000	5.684717000
1	-18.889316000	6.624487000	7.087603000
1	-19.133107000	4.743254000	3.955663000
1	-15.231825000	3.510525000	6.758888000
1	-17.358267000	3.295882000	3.021870000
1	-15.390586000	2.665609000	4.417157000
6	-12.599076000	6.280980000	9.586826000
6	-12.492354000	5.627759000	8.238507000
8	-13.406192000	5.712325000	7.368577000
8	-11.465645000	4.963739000	7.894776000
1	-11.831914000	5.901278000	10.266827000
1	-13.595366000	6.104060000	10.004113000
6	-14.042665000	0.856952000	1.927692000
6	-9.582427000	1.191589000	3.811427000
6	-8.688393000	4.909525000	0.827569000
6	-12.887746000	4.126928000	-1.456405000
6	-12.926594000	0.659791000	2.732155000
6	-12.889762000	-0.222454000	3.874469000
6	-11.617212000	-0.168123000	4.378400000
6	-10.887714000	0.771383000	3.558351000
6	-8.937935000	2.229588000	3.143456000
6	-7.625290000	2.734380000	3.483203000
6	-7.392682000	3.799933000	2.662483000
6	-8.551046000	3.937463000	1.810423000
6	-9.763365000	5.032242000	-0.042918000
6	-9.842340000	6.000495000	-1.115111000
6	-11.014573000	5.768107000	-1.772297000
6	-11.657986000	4.662086000	-1.097185000
6	-13.553738000	3.120060000	-0.763498000

6	-14.903685000	2.688887000	-1.045178000
6	-15.245667000	1.801979000	-0.062246000
6	-14.095941000	1.672471000	0.802943000
7	-11.692382000	1.246932000	2.546857000
7	-9.477043000	2.963029000	2.107736000
7	-10.881697000	4.232875000	-0.044854000
7	-13.070337000	2.471736000	0.350185000
26	-11.325616000	2.816273000	1.310164000
1	-9.065112000	0.740206000	4.653343000
1	-7.873989000	5.618239000	0.714903000
1	-13.399874000	4.572948000	-2.303870000
1	-14.952267000	0.331107000	2.204508000
8	-10.231046000	1.356385000	-0.472042000
8	-8.813931000	-0.336983000	1.115711000
1	-8.621516000	-0.047639000	2.028716000
1	-9.276530000	0.437532000	0.706845000
1	-19.971633000	5.229115000	7.113151000
1	-12.468909000	7.363005000	9.468698000
1	-15.508290000	6.637170000	3.046270000
1	-12.273476000	-0.094850000	-2.513841000
1	-8.280181000	4.003651000	-5.358351000
1	-13.739702000	-0.771762000	4.261801000
1	-11.220756000	-0.665571000	5.254876000
1	-16.195148000	1.302347000	0.087798000
1	-15.515404000	3.056220000	-1.859558000
1	-11.428085000	6.299733000	-2.620108000
1	-9.097989000	6.761120000	-1.314615000
1	-6.529147000	4.452671000	2.640487000
1	-6.995592000	2.342758000	4.272184000
1	-17.003804000	4.955878000	7.680967000
6	-7.770971000	-0.935048000	-4.903051000
6	-7.958392000	-1.303785000	-3.470922000
7	-9.042133000	-2.060162000	-3.031138000
6	-7.283797000	-0.948372000	-2.333077000
6	-9.042456000	-2.144180000	-1.693016000
7	-7.975278000	-1.477809000	-1.256022000
1	-8.588183000	-0.289581000	-5.246024000
1	-7.733765000	-1.818463000	-5.550126000
1	-9.716416000	-2.514873000	-3.636703000
1	-6.402386000	-0.340335000	-2.203138000
1	-9.768784000	-2.654261000	-1.079934000
1	-7.855577000	-1.229851000	-0.263882000
1	-6.832346000	-0.390191000	-5.028274000

^{3}C*c*P SO-Trp W191F

6	-12.856977000	-0.596970000	-1.738967000
7	-12.015333000	-0.742758000	-0.554950000
6	-12.382428000	-1.412915000	0.541625000
7	-13.598430000	-1.998827000	0.602939000
7	-11.489181000	-1.625016000	1.520739000

1	-13.677907000	0.111240000	-1.572205000
1	-13.258619000	-1.568402000	-2.043180000
1	-11.332559000	0.025141000	-0.424902000
1	-14.372613000	-1.508625000	0.170092000
1	-13.838434000	-2.489336000	1.455704000
1	-10.576815000	-1.166458000	1.437769000
1	-11.837159000	-1.652937000	2.479447000
6	-9.070032000	4.293015000	-4.654981000
6	-9.027045000	3.430422000	-3.433649000
6	-10.009877000	2.588153000	-3.038147000
6	-7.843793000	3.379825000	-2.559153000
16	-9.445919000	1.449718000	-1.795810000
6	-7.964122000	2.415299000	-1.544764000
6	-6.689008000	4.162498000	-2.606626000
6	-7.009085000	2.228128000	-0.558214000
6	-5.710649000	3.990616000	-1.619296000
6	-5.872340000	3.045977000	-0.599770000
1	-8.914766000	5.344528000	-4.386702000
1	-10.029814000	4.211499000	-5.170787000
1	-10.977769000	2.426739000	-3.494115000
1	-6.557570000	4.908351000	-3.384570000
1	-7.151336000	1.518540000	0.250206000
1	-4.819830000	4.611091000	-1.637288000
1	-5.123998000	2.955855000	0.181096000
6	-14.934959000	6.434018000	3.954983000
6	-13.789248000	5.500374000	3.724276000
7	-12.943136000	5.078356000	4.736594000
6	-13.382259000	4.901440000	2.546123000
6	-12.079242000	4.244050000	4.138989000
7	-12.290910000	4.097024000	2.808632000
1	-14.587560000	7.423414000	4.283056000
1	-15.615491000	6.055804000	4.723266000
1	-12.840537000	5.659967000	6.337601000
1	-13.799412000	4.983122000	1.550486000
1	-11.281327000	3.734319000	4.665399000
6	-19.304004000	5.818999000	6.472028000
6	-18.176769000	4.928593000	5.988342000
6	-17.060283000	4.672415000	6.801531000
6	-18.197423000	4.366453000	4.704104000
6	-15.990427000	3.896211000	6.347459000
6	-17.130173000	3.587820000	4.241658000
6	-16.022735000	3.353880000	5.058475000
1	-20.206480000	5.685485000	5.865693000
1	-19.020050000	6.878279000	6.415580000
1	-19.050389000	4.552715000	4.054158000
1	-15.118296000	3.751880000	6.979380000
1	-17.154179000	3.189331000	3.229335000
1	-15.167482000	2.798930000	4.683191000
6	-12.599037000	6.280971000	9.586953000
6	-12.862172000	5.472722000	8.318991000

8	-12.685427000	6.187247000	7.211437000
8	-13.199006000	4.295096000	8.337417000
1	-12.739820000	5.646860000	10.463680000
1	-13.282887000	7.136007000	9.630757000
6	-14.074482000	0.899934000	2.170303000
6	-9.538226000	1.196464000	3.870613000
6	-8.740470000	4.875436000	0.819012000
6	-12.952571000	3.971346000	-1.395442000
6	-12.943760000	0.735918000	2.962631000
6	-12.877808000	-0.128591000	4.122746000
6	-11.580701000	-0.104323000	4.560334000
6	-10.867493000	0.807045000	3.688695000
6	-8.900247000	2.217679000	3.166211000
6	-7.574988000	2.725370000	3.460981000
6	-7.371426000	3.785444000	2.624306000
6	-8.558217000	3.910895000	1.805692000
6	-9.841212000	4.980084000	-0.023719000
6	-9.942918000	5.917429000	-1.126775000
6	-11.115853000	5.648184000	-1.765913000
6	-11.738189000	4.551021000	-1.049118000
6	-13.609807000	2.978679000	-0.667078000
6	-14.963665000	2.534023000	-0.915370000
6	-15.298669000	1.704447000	0.121909000
6	-14.139539000	1.626187000	0.982825000
7	-11.706985000	1.286710000	2.714083000
7	-9.464995000	2.936599000	2.138849000
7	-10.952006000	4.175720000	0.014420000
7	-13.118007000	2.385019000	0.467616000
26	-11.367064000	2.806281000	1.421237000
1	-8.997682000	0.749143000	4.700381000
1	-7.935838000	5.590126000	0.673173000
1	-13.472634000	4.382517000	-2.256372000
1	-14.985694000	0.408658000	2.502424000
8	-10.276275000	1.367726000	-0.482783000
8	-8.872244000	-0.286704000	1.138631000
1	-8.681327000	0.004933000	2.054084000
1	-9.334866000	0.492503000	0.736442000
1	-19.561205000	5.606841000	7.516221000
1	-11.578998000	6.680223000	9.570753000
1	-15.509074000	6.567002000	3.031125000
1	-12.231332000	-0.204506000	-2.544098000
1	-8.272134000	4.011171000	-5.353509000
1	-13.724168000	-0.639175000	4.566934000
1	-11.157491000	-0.593993000	5.428595000
1	-16.253845000	1.231284000	0.317892000
1	-15.587867000	2.864669000	-1.736420000
1	-11.543832000	6.146408000	-2.627193000
1	-9.210542000	6.681943000	-1.355512000
1	-6.513610000	4.444845000	2.575669000
1	-6.922959000	2.347087000	4.238567000

1	-17.018901000	5.101566000	7.800748000
6	-7.770989000	-0.935034000	-4.903015000
6	-8.002196000	-1.253246000	-3.462520000
7	-9.141857000	-1.923567000	-3.023411000
6	-7.321907000	-0.920011000	-2.320922000
6	-9.166437000	-1.977054000	-1.683986000
7	-8.061265000	-1.380255000	-1.243174000
1	-8.541773000	-0.249513000	-5.274416000
1	-7.778515000	-1.836792000	-5.525337000
1	-9.848633000	-2.327016000	-3.627059000
1	-6.408680000	-0.361705000	-2.188118000
1	-9.948438000	-2.398918000	-1.072843000
1	-7.956404000	-1.113338000	-0.249267000
1	-6.799767000	-0.450011000	-5.025500000
